# Pak2 as a Novel Therapeutic Target for Cardioprotective Endoplasmic Reticulum Stress Response

**DOI:** 10.1161/CIRCRESAHA.118.312829

**Published:** 2019-02-28

**Authors:** Pablo Binder, Shunyao Wang, Maria Radu, Min Zin, Lucy Collins, Saba Khan, Yatong Li, Karolina Sekeres, Neil Humphreys, Eileithyia Swanton, Adam Reid, Fay Pu, Delvac Oceandy, Kaomei Guan, Susanne S. Hille, Norbert Frey, Oliver J. Müller, Elizabeth J. Cartwright, Jonathan Chernoff, Xin Wang, Wei Liu

**Affiliations:** 1 From the Faculty of Biology, Medicine and Health, The University of Manchester, United Kingdom (P.B., S.W., M.Z., L.C., S.K., Y.L., N.H., E.S., A.R., D.O., E.J.C., X.W., W.L.); 2 Cancer Biology Program, Fox Chase Cancer Center, Philadelphia, PA (M.R., J.C.); 3 Institute of Pharmacology and Toxicology, Faculty of Medicine Carl Gustav Carus, Technische Universitaet Dresden, Germany (K.S., K.G.); 4 Edinburgh University Medical School, United Kingdom (F.P.); 5 Department of Internal Medicine III, University of Kiel, Germany (S.S.H., N.F., O.J.M.); 6 DZHK, German Centre for Cardiovascular Research, Partner Site Hamburg/Kiel/Lübeck, Germany (O.J.M.).

**Keywords:** apoptosis, endoplasmic, reticulum, heart failure, quality, control, tauroursodeoxycholic, acid

## Abstract

**Rationale:**

Secreted and membrane-bound proteins, which account for 1/3 of all proteins, play critical roles in heart health and disease. The endoplasmic reticulum (ER) is the site for synthesis, folding, and quality control of these proteins. Loss of ER homeostasis and function underlies the pathogenesis of many forms of heart disease.

**Objective:**

To investigate mechanisms responsible for regulating cardiac ER function, and to explore therapeutic potentials of strengthening ER function to treat heart disease.

**Methods and Results:**

Screening a range of signaling molecules led to the discovery that Pak (p21-activated kinase)2 is a stress-responsive kinase localized in close proximity to the ER membrane in cardiomyocytes. We found that Pak2 cardiac deleted mice (Pak2-CKO) under tunicamycin stress or pressure overload manifested a defective ER response, cardiac dysfunction, and profound cell death. Small chemical chaperone tauroursodeoxycholic acid treatment of Pak2-CKO mice substantiated that Pak2 loss-induced cardiac damage is an ER-dependent pathology. Gene array analysis prompted a detailed mechanistic study, which revealed that Pak2 regulation of protective ER function was via the IRE (inositol-requiring enzyme)-1/XBP (X-box–binding protein)-1–dependent pathway. We further discovered that this regulation was conferred by Pak2 inhibition of PP2A (protein phosphatase 2A) activity. Moreover, IRE-1 activator, Quercetin, and adeno-associated virus serotype-9–delivered XBP-1s were able to relieve ER dysfunction in Pak2-CKO hearts. This provides functional evidence, which supports the mechanism underlying Pak2 regulation of IRE-1/XBP-1s signaling. Therapeutically, inducing Pak2 activation by genetic overexpression or adeno-associated virus serotype-9–based gene delivery was capable of strengthening ER function, improving cardiac performance, and diminishing apoptosis, thus protecting the heart from failure.

**Conclusions:**

Our findings uncover a new cardioprotective mechanism, which promotes a protective ER stress response via the modulation of Pak2. This novel therapeutic strategy may present as a promising option for treating cardiac disease and heart failure.

## Abstract 2

Supplemental Digital Content is available in the text.

Cardiovascular disease is a major health challenge and remains the leading cause of death worldwide.^1^ Hearts injured from cardiovascular disease eventually progress to heart failure (HF). Despite numerous causative factors, the overwhelming majority of HF cases are the consequence of massive cardiomyocyte loss.^2^ Undoubtedly, the high prevalence and financial burden of HF poses an enormous challenge on us to discover new cardioprotective mechanisms with the hope of developing novel life-saving therapies.

**Meet the First Author, see p 664**

Because cardiomyocytes are terminally differentiated, they hold very little potential in replication. Therefore, exquisite regulation of cellular homeostasis and integrity reliant on protein quality control is crucial for cardiomyocyte survival and function.^3^ The protein quality control carries out a rigorous surveillance process to examine newly synthesized proteins and eliminate aberrantly folded proteins.^4^ A major site of protein quality control is the endoplasmic reticulum (ER), and ≈1/3 of cellular proteins, including membrane and secretory proteins, are synthesized at the ER.^5^ There are 3 key features of the ER lumen that ensure correct folding of secretory or transmembrane proteins: (1) an oxidative environment for disulfide bond formation; (2) abundant chaperone proteins for protein folding; and (3) a high Ca^2+^ concentration favoring Ca^2+^-dependent chaperones-protein interactions.^6^ Any factor perturbing these 3 prerequisites can lead to an accumulation of unfolded/misfolded proteins in the ER, which causes ER stress and triggers the unfolded protein response (UPR).^7^ The UPR serves to relieve ER stress by halting protein translation, increasing chaperone expression for refolding, and degrading irreparably misfolded proteins through a process termed ER-associated degradation (ERAD).^8^ The initial UPR involves the chaperone protein GRP78 (glucose-regulated protein 78), which directly binds Ca^2+^ and senses unfolded or damaged proteins.^9^ The UPR is induced by 3 transmembrane stress sensors: IRE (inositol-requiring enzyme)-1, PERK (protein kinase-like ER kinase), and ATF (activating transcription factor)-6, which are activated by the accumulation of misfolded proteins in the ER.^7^ The initial phase of the UPR is prosurvival, and the 3 branches work in concert to restore ER homeostasis and function. However, prolonged or severe ER stress eventually culminates in damage of folding capacity and the ERAD process, trapping unfolded/misfolded proteins within the ER lumen and switches ER stress to the prodeath phase.^10^

Cardiomyocytes are particularly vulnerable to ER stress because of their poor regenerative capacity and dependence on transmembrane proteins, such as ion channels for contractile processes.^11^ Pressure-overloaded or ischemic-injured hearts often experience oxidative stress, energy deprivation, abnormal calcium content, and inflammation, all of which can disrupt ER folding and induce ER stress.^12^ Investigations using several genetically modified mouse models featuring overexpression of ATF-6, knockout of CHOP (C/EBP homologous protein) in hypertrophic or ischemic conditions clearly suggest that ER stress underpins the pathogenesis of these cardiac conditions.^13,14^ This paradigm is further supported by the evidence that ER lumen expansion, and increased expression of GRP78 and CHOP are present in failing human hearts.^15,16^ Although considerable progress has been made in understanding the ER stress response in the heart, advances in elucidating the mechanistic regulation of the prosurvival UPR pathway are relatively limited.^17^

Here, we report that Pak (p21-activated kinase)2, a Rac1 (Rac family small GTPase 1)/Cdc42 (cell division cycle 42)-activated signaling effector, is a stress-responsive kinase localized in close proximity to the ER membrane in cardiomyocytes. Phenotypic analysis of cardiac Pak2 knockout mice revealed that Pak2 promoted a protective ER stress response under ER stress conditions. Gene array data prompted a mechanistic study demonstrating that Pak2 regulation of the protective ER function was via the IRE-1/XBP-1–dependent pathway. We further discovered that this regulation was conferred by Pak2 inactivation of PP2A (protein phosphatase 2A). This proposed mechanism was corroborated by functional evidence that IRE-1 activator, Quercetin, and AAV (adeno-associated virus serotype)-9–delivered XBP-1s were able to relieve ER dysfunction in Pak2-CKO hearts. Therapeutically, inducing Pak2 activation was capable of promoting the protective ER stress response, thus ameliorating HF progression. Together, our findings unveil a novel cardioprotective mechanism that represents a new route for devising future strategies through modulating the protective ER stress response to treat cardiac disease and subsequent HF.

## Methods

All supporting data are available within the article. Detailed Methods are available in the Online Data Supplement.

## Results

### Pak2 Is Localized in Close Proximity to the ER Membrane

**Figure 1.**
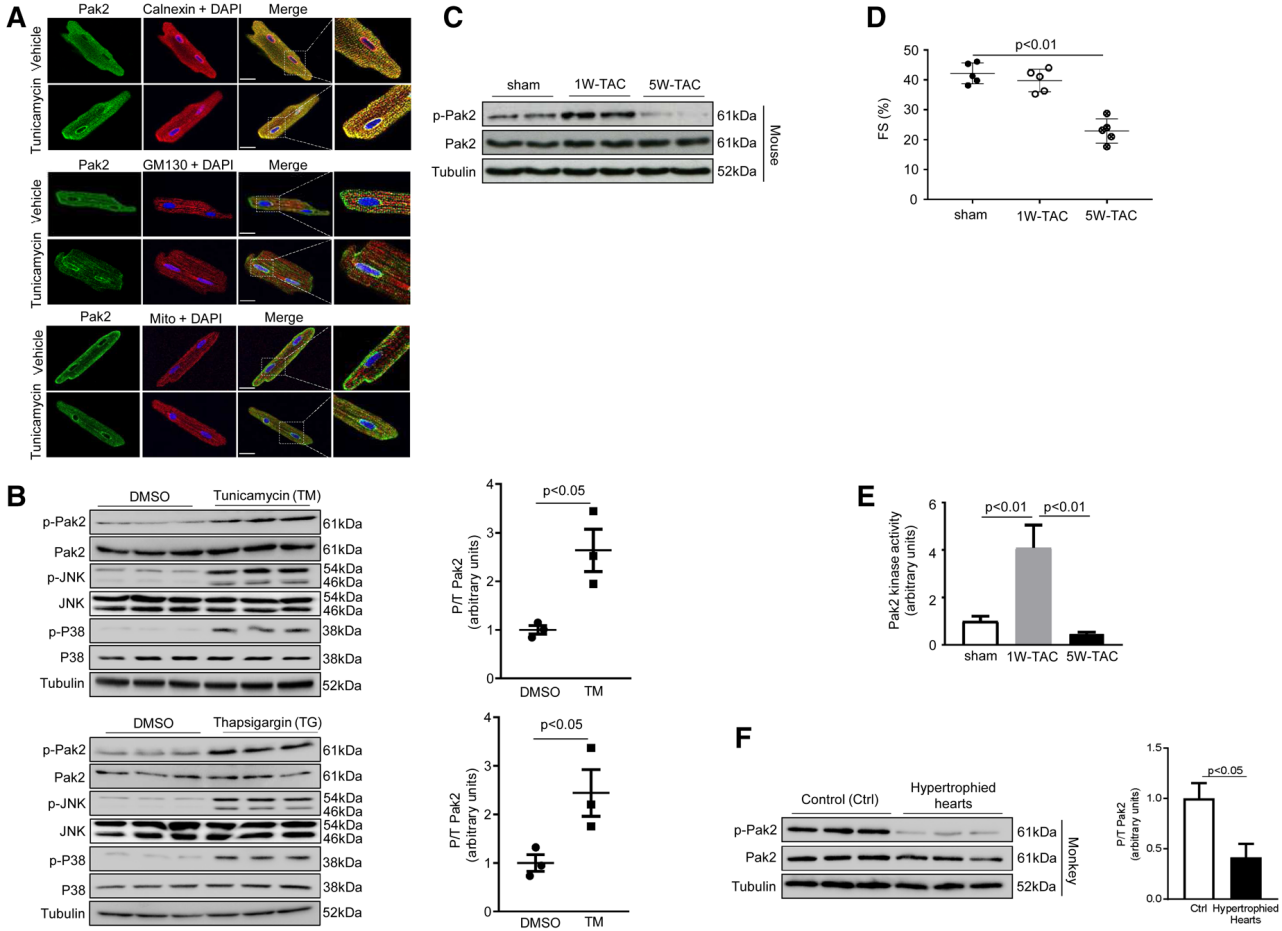
**Pak (p21-activated kinase)2 is activated in response to endoplasmic reticulum (ER) stress. A**, Immunocytochemistry showed colocalization of Pak2 (green) and calnexin (red, an ER marker) in adult rat cardiomyocytes. No colocalization of Pak2 (green) and GM130 (red, Golgi marker) or MitoTracker (red, mitochondria marker) was found. Tunicamycin (TM; 5 μg/mL)-induced prominent Pak2 expression on the ER membrane. DAPI was for nuclei staining (scale bar=20 µm). **B**, Immunoblots of neonatal rat cardiomyocytes under stimulation of TM (5 μg/mL) or thapsigargin (TG; 0.5 µmol/L) for 2 h. **C**, Immunoblots of the hearts of C57BL/6N mice subject to 1 or 5 wk transverse aortic constriction (TAC). **D**, Echocardiography showed the reduced fractional shortening (FS%) in 5W-TAC–stressed hearts. **E**, MEK1 (mitogen-activated protein kinase kinase1) phosphorylation was measured as readout of Pak2 kinase activity (n=6–7). **F**, Immunoblots of the hypertrophied hearts from nonhuman primates (n=6). Tubulin was the loading control. Student *t* test or 1-way ANOVA with Bonferroni correction for post hoc comparisons were used for analyses. Data present as mean±SEM. JNK indicates c-Jun N-terminal kinase.

To identify signal transducers promoting protective ER stress response, we examined the abundance and expression pattern of important cardiac signaling molecules (ERK [extracellular signal–regulated kinase]-1/2, ERK-5, p38, JNK [c-Jun N-terminal kinase], Pak1, Pak2, and PKB [protein kinase B]). Among those, Pak2 was most evidently expressed in close proximity to the ER membrane and in juxtaposition to the nucleus that was detected by immunohistochemistry in rat cardiomyocytes and corroborated by fractional immunoblot analysis (Figure 1A; Online Figure I). On ER stress using tunicamycin (N-glycosylation inhibitor, 5 μg/mL) or thapsigargin (calcium pump inhibitor, 0.5 µmol/L) for 2 hours, Pak2 phosphorylation was increased, and its expression became more prominent on the ER membrane (Figure 1A and 1B; Online Figure IA and IB). Phosphorylation of JNK and p38 was also increased by tunicamycin (Figure 1B). Furthermore, increased Pak2 phosphorylation and kinase activity were detected in C57BL/6N mouse hearts after 1 week of transverse aortic constriction (TAC) (Figure 1C through 1E); however, significantly reduced Pak2 phosphorylation and cardiac dysfunction were observed in the mouse hearts which had undergone prolonged TAC stress for 5 weeks (Figure 1C through 1E). A similar reduction in Pak2 phosphorylation was also found in the hypertrophied hearts of primates, which displayed cardiac dysfunction^18^ (Figure 1F). These data suggest that initial ER stress activates Pak2, whereas prolonged ER stress impairs its activation, and that Pak2 responsiveness to ER stress in cardiomyocytes is evident.

### Defective ER Stress Response, Cardiac Dysfunction, and Profound Cell Death in Pak2-CKO Hearts

**Figure 2.**
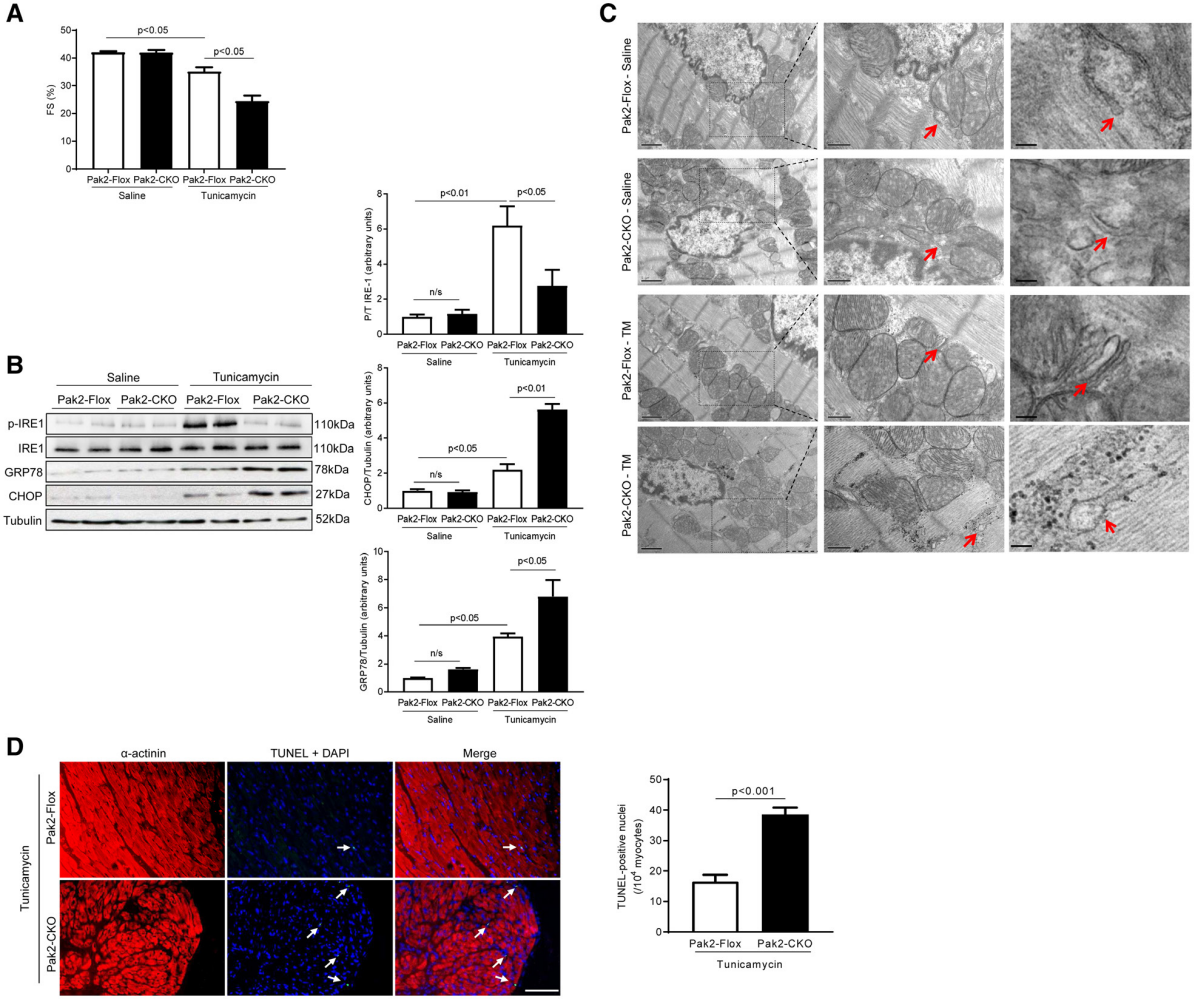
**Cardiac dysfunction and defective endoplasmic reticulum (ER) response are induced by tunicamycin (TM) in Pak (p21-activated kinase)2-CKO hearts. A**, Echocardiographic analyses after 2 d of TM (2 mg/kg) injection (n=6). **B**, Immunoblots and quantification of the hearts under TM stress. **C**, Transmission electron microscopy detected the ultrastructure of ER in left ventricular papillary muscles. Middle images (scale bar=500 nm) are the higher magnifications of boxed areas in the left images (scale bar=1 µm). Right images highlighted the ER structure pointed by arrows in the middle images (scale bar=100 nm). The arrows indicate ER. **D**, TdT-mediated dUTP nick end labeling (TUNEL) assay by triple staining with DAPI (blue), anti-α-actinin (red), and TUNEL (green) determined more apoptosis in Pak2-CKO hearts with TM (scale bar=20 µm), arrows indicate TUNEL positive nuclei. Quantification of TUNEL positive nuclei is represented (n=6). Student *t* test or 2-way ANOVA with Bonferroni correction for post hoc comparisons were used for analyses. Data present as mean±SEM. CHOP indicates C/EBP homologous protein; FS, fractional shortening; IRE, inositol-requiring enzyme; and n/s, not significant.

**Figure 3.**
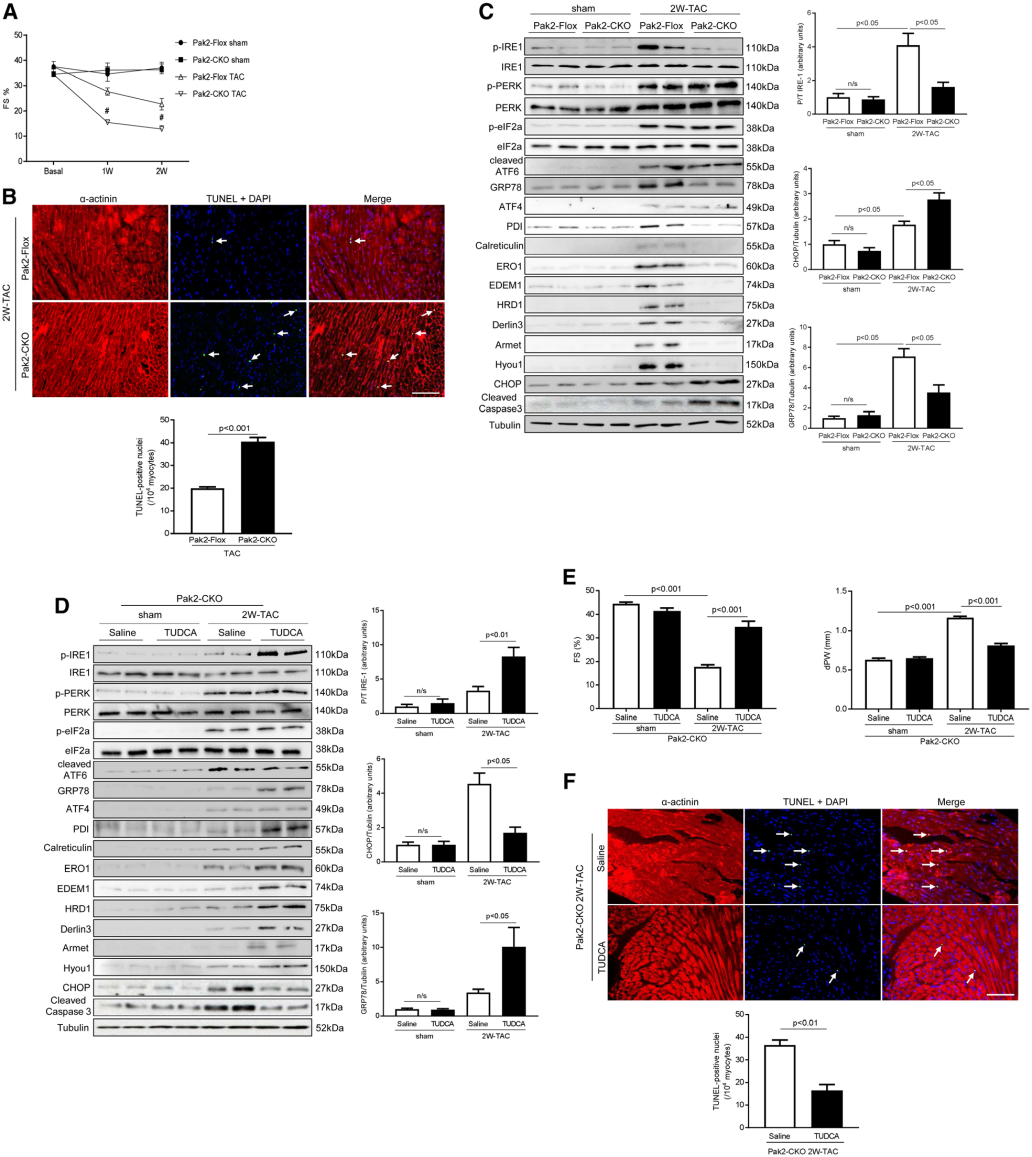
**The cardioprotective role of Pak (p21-activated kinase)2 is endoplasmic reticulum (ER) response-dependent. A**, Echocardiographic analysis (n=7–8). #*P*<0.05 Pak2-CKO vs Pak2-Flox after transverse aortic constriction (TAC). **B**, TdT-mediated dUTP nick end labeling (TUNEL) assay of the hearts after TAC (scale bar=0 µm), arrows indicate TUNEL positive nuclei (n=7–8). Immunoblots and quantification of ER stress markers in (**C**) TAC-stressed hearts or (**D**) tauroursodeoxycholic acid (TUDCA)-injected (300 mg/kg per day) Pak2-CKO hearts. **E**, Echocardiographic assessments and (**F**) TUNEL assay of Pak2-CKO hearts treated with TUDCA (scale bar=20 µm), arrows indicate TUNEL positive nuclei (n=6). Student *t* test or 2-way ANOVA with Bonferroni correction for post hoc comparisons were used for analyses. Data present as mean±SEM. Armet indicates arginine-rich, mutated in early-stage tumors; ATF, activating transcription factor; CHOP, C/EBP homologous protein; EDEM, ER degradation enhancing alpha-mannosidase–like protein; eIF2a, eukaryotic translation initiation factor 2A; ERO, ER oxidoreductin; FS, fractional shortening; HRD, HMG-CoA reductase degradation-1 homolog; Hyou, hypoxia upregulated protein; IRE, inositol-requiring enzyme; n/s, not significant; PERK, protein kinase-like ER kinase; and PDI, protein disulfide isomerase.

To mimic a scenario of dampened Pak2 activation in pathologically stressed hearts, Pak2-CKO mice were generated using Pak2-Flox mice crossed with α-MHC (α-myosin heavy chain)-Cre line. These mice were viable and developed to adulthood without obvious morphological or functional abnormalities. They were used in this study to obtain functional evidence linking Pak2 to the cardiac ER stress response (Online Figure II). Under systemic ER stress induced by tunicamycin (single dose intraperitoneal injection of 2 mg/kg) for 48 hours, Pak2-CKO mice exhibited severe cardiac dysfunction (fractional shortening: 25.86±0.76% compared with 35.08±1.63% in controls), an exacerbated ER response with blunted IRE-1 phosphorylation and significantly increased expression of GRP78 and CHOP (Figure 2A and 2B). Transmission electron microscopy examination revealed an expanded ER lumen in the myocardium of tunicamycin-injected Pak2-CKO mice (Figure 2C). Meanwhile, profound apoptosis was observed; the number of TdT-mediated dUTP nick end labeling-positive nuclei was nearly 2× more than that of controls (Figure 2D; Online Figure III). In addition, α-MHC-Cre hearts did not display distressing responses to tunicamycin (Online Figure IV). Next, we used TAC to provoke cardiac disease-mimetic ER stress and examined mouse heart function at weekly intervals. Fractional immunoblots showed an increased Pak2 in the organelle preparation in Pak2-Flox hearts subjected to TAC (Online Figure V). After 2 weeks of TAC, Pak2-CKO mice developed cardiac dysfunction and pronounced cardiomyocyte apoptosis, but less fibrosis compared with Pak2-Flox and α-MHC-Cre mice (Figure 3A and 3B; Online Figures VI through VIII; Online Table I). Consistent with these findings, augmented protein levels of CHOP and cleaved caspase-3, but decreased GRP78 expression and IRE-1 phosphorylation were detected in the Pak2-CKO heart (Figure 3C). We have also checked a wide range of ER stress molecules and discovered that expression levels of Edem (ER degradation enhancing alpha-mannosidase–like protein)-1, Derlin-3, calreticulin, PDI (protein disulfide isomerase), HRD-1 (HMG-CoA reductase degradation-1 homolog), ERO (ER oxidoreductin)-1, Armet (arginine-rich, mutated in early-stage tumors), and Hyou (hypoxia upregulated protein)-1 were less in Pak2-CKO hearts after TAC, whereas expression of p-PERK, PERK, ATF-4, p-eIF2a (eukaryotic translation initiation factor 2A), eIF2a, and cleaved ATF-6 was comparable between CKO and control mice (Figure 3C).

To corroborate these results, we tested the ability of a small chemical chaperone, tauroursodeoxycholic acid (TUDCA),^19^ to evaluate whether it could relieve Pak2 loss induced-ER dysfunction in a pressure-overloaded heart. TUDCA (intraperitoneal injection of 300 mg/kg per day) was applied on Pak2-Flox and α-MHC-Cre mice for 2 weeks, commencing on the second day after TAC. TUDCA improved cardiac function and ER stress response (Online Figure IX). TUDCA also markedly alleviated the pernicious ER stress response in Pak2-CKO heart, leading to a reduced expression of CHOP and less cleaved caspase-3, whereas IRE-1 phosphorylation was enhanced, and expression levels of Edem-1, Derlin-3, PDI, HRD-1, ERO-1, Armet, and Hyou-1 were restored (Figure 3D). Meanwhile, improved cardiac performance and less apoptosis were detected (Figure 3E and 3F; Online Figure X). The above data demonstrate that Pak2 loss-induced cardiac damage is an ER-dependent pathology.

### Altered Gene Profile of Chaperones and ERAD Components in Pak2-CKO Hearts

**Figure 4.**
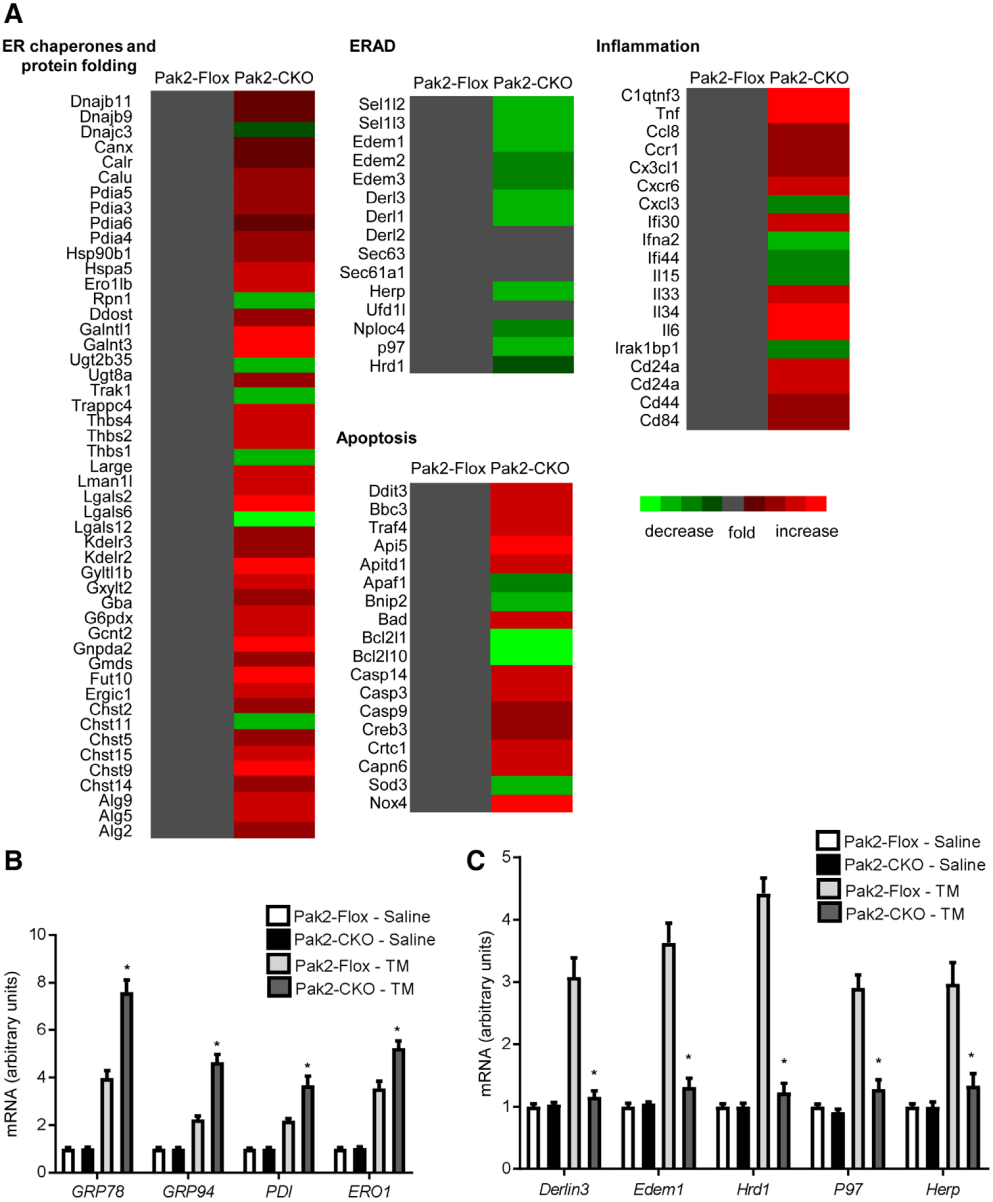
**Altered gene profile in Pak (p21-activated kinase)2-CKO hearts stressed by tunicamycin (TM). A**, The diagrams determined differentially expressed genes (in the categories of endoplasmic reticulum [ER] chaperones and protein folding, ER-associated degradation [ERAD], apoptosis, and inflammation) by Affymetrix gene array. Gene expression profiling in Pak2-Flox hearts was normalized to gray. Red represents the increased expression, whereas green represents the decreased expression (n=2). Quantitative polymerase chain reaction analyses showed (**B**) increased transcript levels of ER chaperones and (**C**) decreased transcript levels of ERAD components in Pak2-CKO hearts with TM injection (n=5). *P<0.05 Pak2-CKO-TM vs Pak2-Flox-TM. Two-way ANOVA with Bonferroni correction for post hoc comparisons was used for analyses. Data present as mean±SEM.

Next, we performed Affymetrix gene array analysis (Mouse Genome 430 2.0 arrays) to gain molecular insights into mechanisms responsible for the defective ER response and cardiac dysfunction in the Pak2-CKO heart. Interestingly, of 45 000 analyzed transcripts, 467 genes were significantly altered (*P*<0.05) in Pak2-CKO hearts in the tunicamycin-induced ER stress condition. Functional annotation by DAVID version 6.2 revealed that ER chaperones (Figure 4A) and apoptosis/inflammation were the main overrepresented clusters. Changes in *GRP78*, *GRP94*, *PDI*, *ERO1* for N-glycosylation, disulfide bond formation and quality control, *CHOP*, *TNF*, *IL-6* for execution of apoptosis were marked. Quantitative polymerase chain reaction (qPCR) analyses further validated the array data and confirmed that protein folding, assembly, and quality control chaperones were noticeably enhanced in the Pak2-CKO heart treated by tunicamycin (Figure 4B). Intriguingly, molecules involved in ERAD, such as *Derlin3*, *Edem1*, *p97*, *Herp*, and *Hrd1*, failed to be upregulated (Figure 4C), which was consistent with what we observed in 2 weeks TAC stressed-Pak2 CKO hearts (Figure 3C). These ERAD components are known to be transcriptional targets of XBP-1.^20^ XBP-1 is able to work on both ER stress element and UPR element, which enables it to regulate not only chaperones, but also ERAD components.^20^

### Pak2 Potentiates Protective ER Stress Response Through IRE-1/XBP-1 Signaling

**Figure 5.**
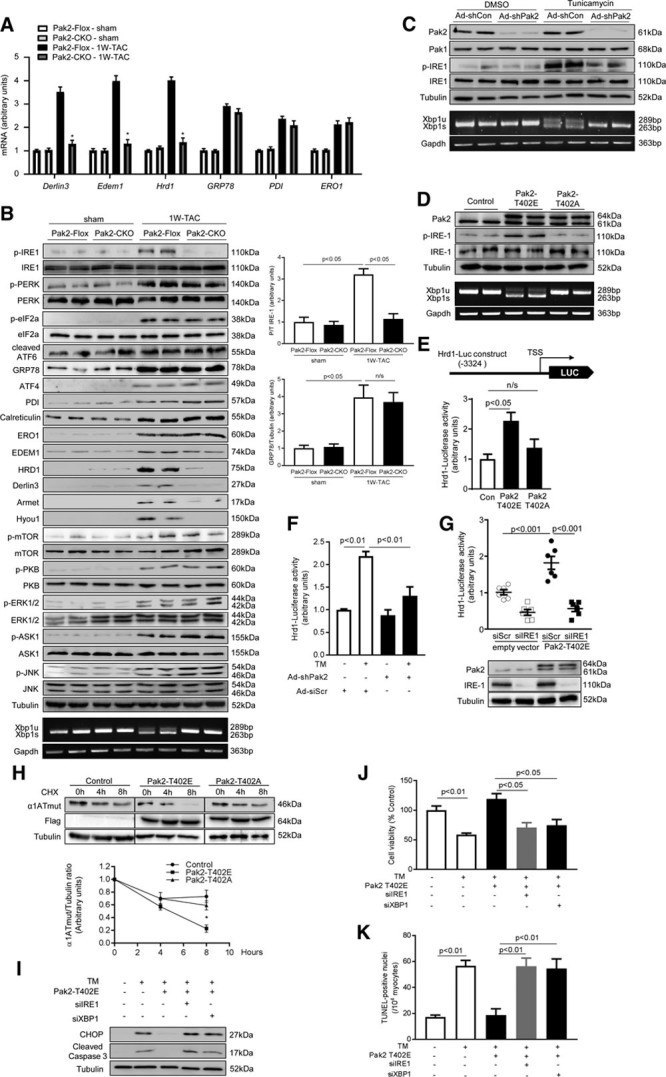
**Pak (p21-activated kinase)2 potentiates protective endoplasmic reticulum (ER) stress response through IRE (inositol-requiring enzyme)-1/XBP (X-box–binding protein)-1 signaling. A**, Quantitative polymerase chain reaction (qPCR) analyses, (**B**) immunoblot analyses and quantification of multiple ER stress markers on the hearts in response to 1W-transverse aortic constriction (TAC). *P<0.05 Pak2-CKO vs Pak2-Flox after TAC. **B**, **Lower**, PCR detected both unspliced XBP-1 (XBP-1u) and spliced XBP-1 (XBP-1s) in Pak2-Flox after TAC; however, XBP-1s was barely detected in Pak2-CKO hearts. **C**, Immunoblots of Pak2-knockdown neonatal rat cardiomyocytes (NRCMs; Ad-shPak2) under tunicamycin (TM) treatment for 6 h. **D**, Immunoblots detected endogenous Pak2 (61 kDa) and Flag-tagged ectopic Pak2 (64 kDa) after overexpression of Pak2-T402E or Pak2-T402A. **E**, Increased Hrd1 reporter luciferase activity was detected in Pak2-T402E overexpressed H9C2 cells (n=5–6). **F**, TM-induced luciferase activity was blunted by Pak2 knockdown (n=4–6). **G**, Pak2-T402E overexpression-induced Hrd1 activity was reduced by IRE-1 knockdown in H9C2 cells. **H**, Immunoblots showed that Pak2-T402E fastened the clearance of mutant protein of α1ATmut (α1-antitrypsin) in H9C2 cells, whereas Pak2-T402A retarded to clear α1ATmut in the presence of 100 μg/mL of cycloheximide (CHX; n=5–7). *P<0.05 Pak2-T402E vs control. **I**, Decreased CHOP (C/EBP homologous protein) and cleaved caspase-3 in Pak2-overexpressed NRCMs were blunted by knockdown IRE-1 or XBP-1. **J**, MTT assay and (**K**) TdT-mediated dUTP nick end labeling (TUNEL) assay showed that Pak2 overexpression inhibited TM-induced apoptosis (n=5–6). One- or 2-way ANOVA with Bonferroni correction for post hoc comparisons were used for analyses. Data present as mean±SEM. Armet indicates arginine-rich, mutated in early-stage tumors; ATF, activating transcription factor; CHOP, C/EBP homologous protein; EDEM, ER degradation enhancing alpha-mannosidase–like protein; eIF2a, eukaryotic translation initiation factor 2A; ERK, extracellular signal–regulated kinase; ERO, ER oxidoreductin; FS, fractional shortening; HRD, HMG-CoA reductase degradation-1 homolog; Hyou, hypoxia upregulated protein; IRE, inositol-requiring enzyme; JNK, c-Jun N-terminal kinase; mTOR, mammalian target of rapamycin; n/s, not significant; PDI, protein disulfide isomerase; PERK, protein kinase-like ER kinase; and PKB, protein kinase B.

Prompted by the gene array data that XBP-1–dependent gene regulation was impaired in tunicamycin stressed Pak2-CKO hearts, we then examined whether this impairment occurred in TAC-stressed Pak2-CKO hearts. qPCR analysis demonstrated that ERAD genes, but not chaperones, were not upregulated in response to 1-week TAC when Pak2 was absent (Figure 5A). Meanwhile, we then sought evidence on whether Pak2 regulates the ER stress response through the IRE-1/XBP-1 branch. We indeed detected blunted IRE-1 phosphorylation and a reduced level of active spliced XBP-1 (XBP-1s) in the Pak2-CKO heart 1 week after TAC, which was considered as a time point to observe changes in the signaling cascade (Figure 5B). We also discerned that phosphorylation of ASK-1 (apoptosis signal-regulating kinase 1) and JNK-1/2 was enhanced in the Pak2-CKO hearts, but PERK phosphorylation and ATF-6 activation were comparable to control mice (Figure 5B). In addition, we found reduced expression of Derlin-3, HRD-1, Armet, and Hyou-1 in the Pak2-CKO hearts (Figure 5B). Hypertrophic responses of Pak2-CKO mice were enhanced after TAC. Herein, expression and phosphorylation of prohypertrophic molecules PKB, ERK-1/2, and mTOR (mammalian target of rapamycin) were checked, but no significant change was found between Pak2-CKO and control mice (Figure 5B).

A similar phenomenon was observed in neonatal rat cardiomyocytes (NRCMs); Pak2-knockdown–inhibited IRE-1 phosphorylation and XBP-1 splicing induced by tunicamycin (Figure 5C). Conversely, forced expression of constitutively active Pak2 (T402E) in NRCMs was sufficient to increase IRE-1 phosphorylation at Ser724 and XBP-1 splicing without additional stressors. Interestingly, overexpression of Pak2 kinase-dead form (T402A) did not induce such increases (Figure 5D). Furthermore, to ascertain whether Pak2 is an ER-associated molecule, we constructed pEF/myc/ER-Pak2 (T402A)-KDEL and pEF/myc/ER-Pak2 (T402E)-KDEL vectors, which delivered Pak2-T402A and Pak2-T402E into the ER lumen. ER lumen retention of the kinase-dead form and kinase-active form did not affect the activation of IRE-1/XBP-1 signaling (Online Figure XIA and XIB). However, we used a vector having Ribophorin I (an ER transmembrane glycoproteins) N-terminal transmembrane domain fused with Pak2-T402A, which directed Pak2-T402A expression anchoring at the ER membrane, and Pak2-T402A was found to impair the activation of IRE-1/XBP-1s signaling (Online Figure XIC and XID). These data clearly indicate that Pak2 is not an ER lumen-resident protein, but is localized in close proximity to the ER membrane for activating IRE-1/XBP-1s in the cytosol.

Next, we investigated whether Pak2 impacted on XBP-1 transcriptional activity through IRE-1. A luciferase reporter gene carrying human Hrd1 promoter region (−1 to −3324 containing XBP-1–specific UPR element sites) was used in NRCMs. Intensified Hrd1 luciferase activity was induced by overexpression of Pak2 (T402E) but not its kinase-dead form (T402A; (Figure 5E). Tunicamycin-induced Hrd1 promoter activity was reduced by Pak2 knockdown (Figure 5F). In addition, Pak2 overexpression-induced Hrd1 activity was reduced by IRE-1 knockdown (Figure 5G). In addition, we evaluated whether Pak2 promoted XBP-1 activity on ERAD function. A mutant protein of α1ATmut (α1-antitrypsin), an ER luminal misfolded protein, was chosen as an ERAD substrate. Coinfection of Ad-α1ATmut and Ad-Pak2-T402E in NRCMs followed by cycloheximide (100 μg/mL) chase experiments was found to enhance the clearance of α1ATmut within a time window of 4 to 8 hours, whereas this clearance was retarded by XBP-1 knockdown (Figure 5H). Furthermore, tunicamycin-triggered aggregation of ubiquitinated proteins was abolished in Pak2-T402E overexpressed cardiomyocytes (Online Figure XII). Finally, we discovered that Pak2 overexpression protected cardiomyocytes from tunicamycin-induced apoptosis, however, this protective effect was blunted by knockdown IRE-1 or XBP-1 (Figure 5I through 5K). The data above suggest that cardioprotection by Pak2 regulation of ER function is through the IRE-1/XBP-1–dependent pathway.

### Pak2 Stimulates IRE-1/XBP-1 Signaling Through Inactivation of PP2A Activity

**Figure 6.**
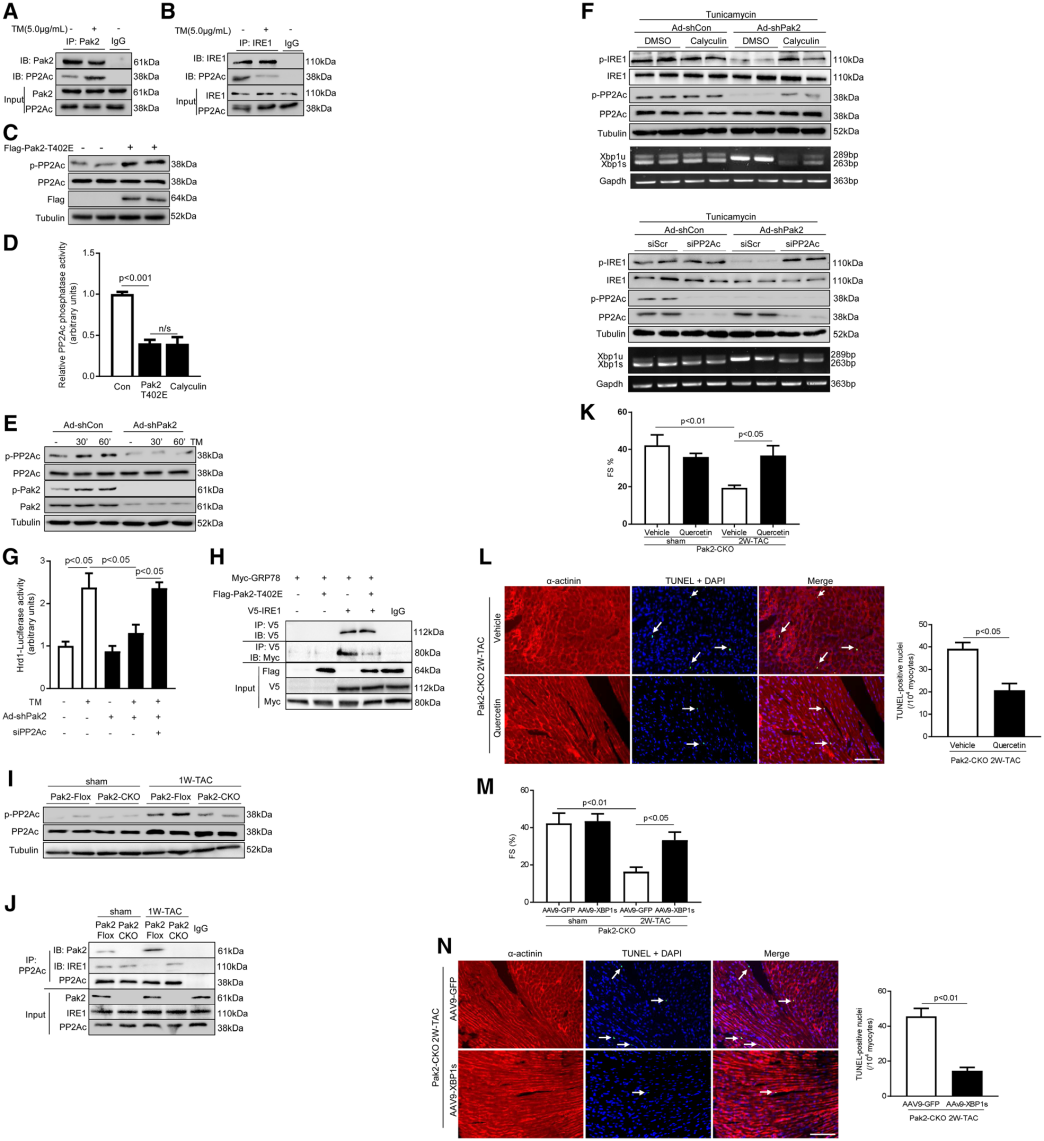
**Pak (p21-activated kinase)2 stimulates IRE (inositol-requiring enzyme)-1/XBP (X-box–binding protein)-1 signaling through inactivation of PP2A (protein phosphatase 2A) activity. A**, Association of endogenous PP2Ac with Pak2 or (**B**) IRE-1 and PP2Ac was observed by immunoprecipitation in neonatal rat cardiomyocytes (NRCMs), whereas tunicamycin (TM) disrupted the interactions. IgG was controlled for nonspecific interaction. **C**, Immunoblots showed that PP2Ac phosphorylation was increased by overexpression of Pak2-T402E in NRCMs. **D**, PP2Ac phosphatase activity assay in H9C2 detected that PP2Ac activity was decreased by Pak2-T402E and calyculin (10 nmol/L, pan-PP2A pharmacological inhibitor; n=5–7). **E**, Immunoblots showed that PP2Ac phosphorylation induced by TM was blocked by Pak2 knockdown in NRCMs. **F**, Immunoblots displayed that under TM, blunted IRE-1 phosphorylation in Pak2-knockdown NRCMs was restored by calyculin (**upper**) or PP2Ac knockdown (siPP2Ac, **lower**). Polymerase chain reaction (PCR) determined that XBP-1 splicing in Pak2-knockdown NRCMs was reconciled by calyculin or siPP2Ac. **G**, Hrd1 reporter luciferase activity induced by TM was blocked by Pak2 knockdown; however, PP2Ac knockdown restored this activity in H9C2 cells (n=5–6). **H**, Immunoprecipitation showed that Pak2-T402E disrupted association of GRP78 (glucose-regulated protein 78; Myc-tagged) with IRE-1 (V5-tagged) in H9C2 cells. **I**, Immunoblots showed increased PP2Ac phosphorylation in Pak2-Flox-transverse aortic constriction (TAC) hearts, but not in Pak2-CKO hearts. **J**, Immunoprecipitation detected association of PP2Ac and IRE-1 in Pak2-CKO myocardium after TAC. **K**, Echocardiographic assessment and (**L**) TdT-mediated dUTP nick end labeling (TUNEL) assay of Pak2-CKO hearts treated with Quercetin (10 mg/kg; scale bar=20 µm, n=6). **M**, Echocardiographic assessment and (**N**) TUNEL assay of adeno-associated virus serotype-9 (AAV-9)-XBP-1s injected Pak2-CKO hearts (n=6). One- or 2-way ANOVA with Bonferroni correction for post hoc comparisons were used for analyses. Data present as mean±SEM. FS indicates fractional shortening.

Next, we attempted to dissect the molecular basis by which Pak2 elicited IRE-1/XBP-1 signaling. Given that Pak2 increased IRE-1 phosphorylation at Ser724, which is a key site for its autophosphorylation, we first probed whether Pak2 acted on IRE-1 phosphorylation through direct association of IRE-1, causing its conformation change, and then inducing its autophosphorylation. However, immunoprecipitation assays in various experimental conditions failed to detect either endogenous or exogenous Pak2 interaction with IRE-1 (Online Figure XIIIA and XIIIB). We then investigated whether Pak2 instigated IRE-1 phosphorylation through negative regulation of PP2A, a protein phosphatase able to dephosphorylate IRE-1.^21^ Based on this assumption, we performed a series of immunoprecipitation assays and observed that Pak2 interacted with the catalytic subunit of PP2A (PP2Ac) endogenously and exogenously (Figure 6A; Online Figure XIIIB). IRE-1 association with PP2Ac was also detected, but this interaction was disrupted by tunicamycin stress (Figure 6B). Because PP2Ac phosphorylation at Tyr307 inhibits its phosphatase activity,^22^ we then detected PP2Ac phosphorylation at Tyr307 and its activity. Indeed, we discovered that Pak2-T402E overexpression in NRCMs increased PP2Ac phosphorylation at Tyr307, but decreased its phosphatase activity (Figure 6C and 6D). Conversely, Pak2-knockdown–prevented PP2Ac phosphorylation at Tyr307 in tunicamycin-treated NRCMs (Figure 6E). In addition, we detected that Pak2-T402E was able to directly phosphorylate recombinant PP2Ac using in vitro kinase assay (Online Figure XIV). Furthermore, reductions in IRE-1 phosphorylation and XBP-1 splicing caused by Pak2 knockdown were restored by PP2Ac knockdown or the treatment of the PP2A inhibitor, Calyculin (Figure 6F). Consistent with these findings, reduced Hrd1 promoter activity caused by Pak2 knockdown was recovered by PP2Ac knockdown (Figure 6G). Also, we noticed that similar to the tunicamycin effect on promoting GRP78 dissociation from IRE-1, Pak2-T402E overexpression was able to cause dissociation of IRE-1 and GRP78 without additional stressors (Figure 6H). Moreover, PP2Ac phosphorylation at Tyr307 was increased in pressure-overloaded Pak2-Flox hearts, but not in Pak2-CKO hearts (Figure 6I). Consistent with this, association of PP2Ac and IRE-1 was detected in Pak2-CKO myocardium after TAC, but not in Flox hearts (Figure 6J). We have also checked PP2Ce, another IRE-1–specific phosphatase,^23^ but did not find an association of Pak2 or IRE-1 with PP2Ce (Online Figure XVA). Meanwhile, PP2Ce phosphatase activity was unchanged in Pak2-overexpressed cardiomyocytes (Online Figure XVB).

In a further attempt, we performed rescue experiments in Pak2-CKO hearts, to obtain functional evidence for supporting the proposed mechanism responsible for Pak2 regulation of IRE-1/XBP-1 signaling. To do so, we administrated an IRE-1 activator, Quercetin, to Pak2-CKO mice by gavage on the second day after TAC (10 mg/kg per day). After 2 weeks, compared with vehicle-TAC group and sham groups, CKO hearts receiving Quercetin displayed improved cardiac performance and less cardiomyocyte death (Figure 6K and 6L; Online Figure XVIA). Meanwhile, hypertrophic response was reduced; increased IRE-1 phosphorylation and augmented XBP-1s expression were observed (Online Figure XVIB and XVIC). Second, AAV-9–packed XBP-1s under a TnT (troponin T) promoter was injected on the same day as TAC commencing. After 2 weeks of TAC, forced expression of XBP-1s relieved distressing ER stress responses in Pak2-CKO hearts, as well as showing reduced cardiac hypertrophy, improved cardiac performance, and less apoptosis (Figure 6M and 6N; Online Figure XVID through XVIF).

Collectively, these data illustrate that the signaling mechanism for the IRE-1/XBP-1–dependent protective ER stress responses in the heart is likely through Pak2 inactivation of PP2A.

### Pak2-Dependent Protective ER Stress Response in Human Cardiomyocytes

**Figure 7.**
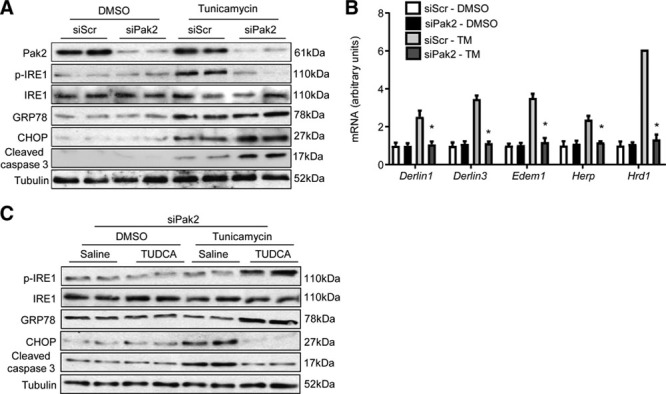
**Pak (p21-activated kinase)2-dependent protective endoplasmic reticulum (ER) stress response in human-induced pluripotent stem cell-derived cardiomyocytes (iPSC-CMs). A**, Immunoblotting and (**B**) quantitative polymerase chain reaction (qPCR) analyses of tunicamycin (TM; 2 µg/mL) stressed iPSC-CMs (n=5–6, *P<0.05 siPak2 vs siScr [scramble siRNA] with TM stimulation for 6 h). Two-way ANOVA with Bonferroni correction for post hoc comparisons was used for analyses. Data present as mean±SEM. **C**, The diminished IRE (inositol-requiring enzyme)-1 phosphorylation in Pak2-knockdown iPSC-CMs was restored by tauroursodeoxycholic acid (TUDCA) treatment (200 µg/mL), whereas CHOP (C/EBP homologous protein) and cleaved caspase-3 were reduced by TUDCA.

To seek human-relevant data for this cardioprotective action, we evaluated the Pak2-entailed ER stress response and beneficial effects of TUDCA in human-induced pluripotent stem cell-derived cardiomyocytes. Knockdown of Pak2 in induced pluripotent stem cell-derived cardiomyocytes markedly reduced tunicamycin-induced IRE-1 phosphorylation, consequently, increased expression of CHOP and cleaved caspase-3 was detected (Figure 7A). qPCR analyses showed that mRNA expression level of molecules involved in ERAD (*Derlin1*, *Derlin3, Edem1, Herp*, and *Hrd1*) failed to respond to tunicamycin stimulation in Pak2-knockdown–induced pluripotent stem cell-derived cardiomyocytes (Figure 7B). However, TUDCA treatment considerably increased IRE-1 phosphorylation and GRP78 expression, while reduced expression of CHOP and cleaved caspase-3 (Figure 7C; Online Figure XVII).

### Therapeutic Potential of Pak2 Activation by Promoting Protective ER Function

**Figure 8.**
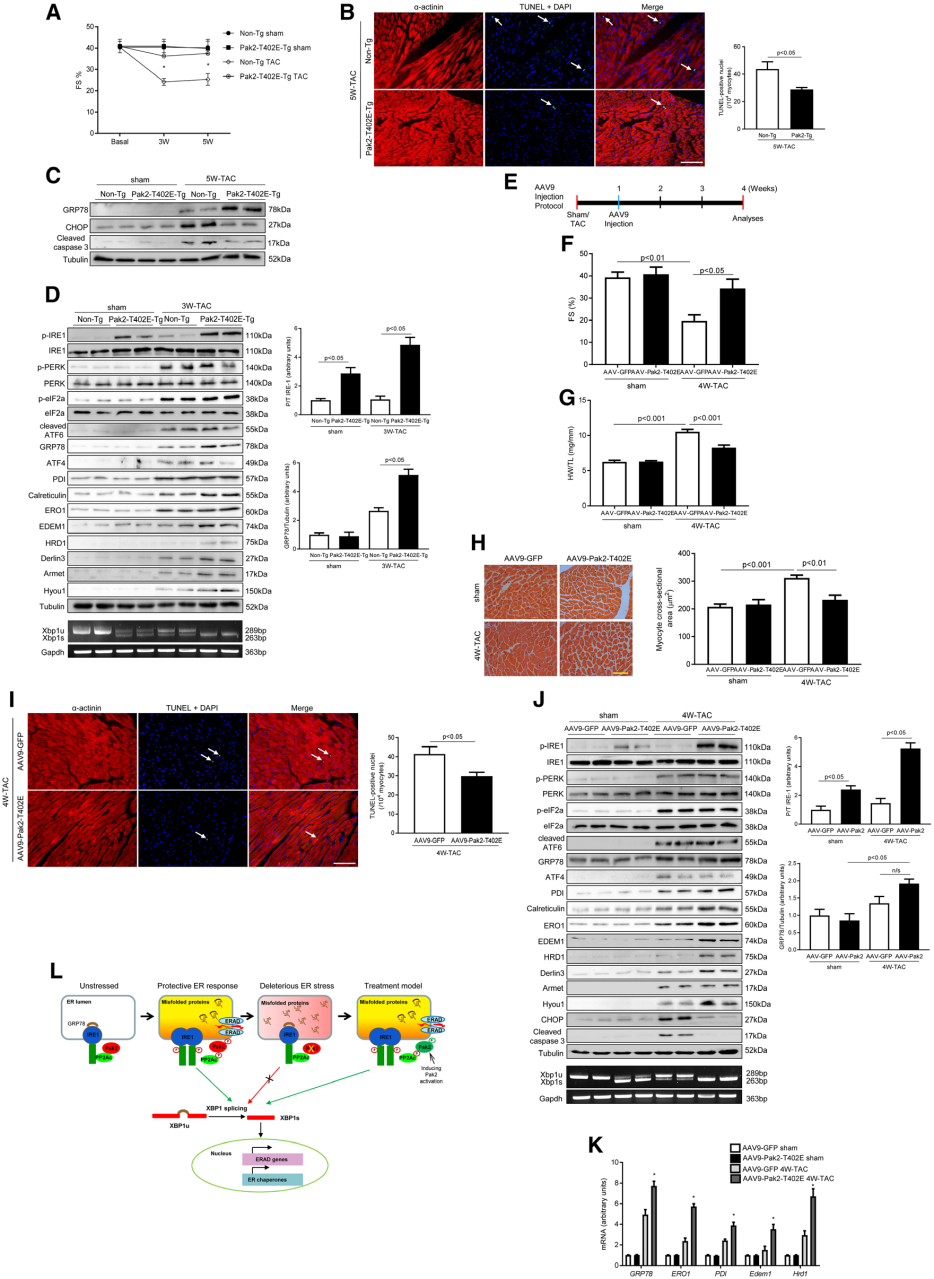
**Therapeutic potential of Pak (p21-activated kinase)2 activation is to protect endoplasmic reticulum (ER) function. A**, Echocardiographic analyses of Pak2 cardiac overexpression mice (Pak2-T402E-Tg; n=6–7, *P<0.05 non-Tg vs Pak2-T402E-Tg under transverse aortic constriction [TAC]). **B**, TdT-mediated dUTP nick end labeling (TUNEL) assay of TAC-stressed hearts (scale bar=20 µm, n=6–7). **C**, Immunoblots and quantification showed augmented GRP78 (glucose-regulated protein 78) expression but reduced CHOP (C/EBP homologous protein) and cleaved caspase-3 in Pak2-T402E-Tg mice after 5 wk of TAC. **D**, Detection of multiple ER stress markers in the hearts after 3 wk TAC. **E**, Schematic timeline of adeno-associated virus serotype-9 (AAV-9)-Pak2-T402E injection. **F**, Echocardiography of AAV-9-Pak2-T402E-injected mice (n=7–8, #*P*<0.05 AAV-9-Pak2-T402E vs AAV-9-GFP under TAC). **G**, Heart weight/tibia length ratio (HW/TL) and (**H**) myocyte cross-sectional areas (scale bar=20 µm, n=7) determined less cardiac hypertrophy in AAV-9-Pak2-T402E-injected mice subject to TAC. **I**, Decreased apoptosis in AAV-9-Pak2-T402E-injected hearts was detected by TUNEL assay (scale bar=20 µm, n=7–8). **J**, Immunoblots and quantification of multiple ER stress markers in AAV-9-Pak2-T402E-injected mice. Polymerase chain reaction (PCR) was to show XBP (X-box–binding protein)-1 splicing level. **K**, Transcript levels of ER chaperones and ER-associated degradation (ERAD) components were examined by quantitative PCR (n=7–8, *P<0.05 AAV-9-Pak2-T402E vs AAV-9-GFP under TAC). Student *t* test or 2-way ANOVA with Bonferroni correction for post hoc comparisons were used for analyses. Data present as mean±SEM. **L**, Schematic diagram of Pak2 cardioprotection against deleterious ER stress. Armet indicates arginine-rich, mutated in early-stage tumors; ATF, activating transcription factor; CHOP, C/EBP homologous protein; EDEM, ER degradation enhancing alpha-mannosidase–like protein; eIF2a, eukaryotic translation initiation factor 2A; ERK, extracellular signal–regulated kinase; ERO, ER oxidoreductin; FS, fractional shortening; HRD, HMG-CoA reductase degradation-1 homolog; Hyou, hypoxia upregulated protein; IRE, inositol-requiring enzyme; JNK, c-Jun N-terminal kinase; mTOR, mammalian target of rapamycin; n/s, not significant; PDI, protein disulfide isomerase; PERK, protein kinase-like ER kinase; PKB, protein kinase B; and PP2A, protein phosphatase 2A.

Moving forward, we endeavored to translate the above-described signaling mechanism to derive therapeutic options for the alleviation of ER dysfunction in cardiac disease. First, we generated Pak2 cardiac overexpression mice under an MHC-promoter (Pak2-T402E-Tg) to investigate whether Pak2 activation, constituted from developmental stage, could render the mice resistant to TAC-induced ER stress. A few Pak2-T402E-Tg mouse lines were analyzed and line 3.22 was chosen for this study. Pak2 expression level in this line at 8 weeks of age was ≈4× of that in non-Tg mice (Online Figure XVIIIA). Fractional immunoblots demonstrated a greater amount of Pak2 expression in the cytosol and organelle preparation (Online Figure XVIIIB). The Pak2-T402E-Tg mice developed to term and appeared normal, which suggests that Pak2 overexpression and activation in the hearts did not affect their development (Online Figure XVIIIC and XVIIID), therefore line 3.22 was a viable model for studying the role of Pak2 in ER stress. Pressure overload by TAC was applied to Pak2-T402E-Tg mice and we analyzed their cardiac function on a weekly basis. Strikingly, Pak2-T402E-Tg mice were resistant to rigorous TAC stress up to 5 weeks, displaying preserved cardiac performance and less cardiomyocyte death (Figure 8A and 8B; Online Table II; Online Figure XVIIIE). It is worthy of note that cardiac fibrosis content was less in Pak2-T402E-Tg than non-Tg mice (Online Figure XVIIIF). CHOP and cleaved caspase-3 were found to be at a lower expression level while GRP78 level was increased compared with non-Tg mice (Figure 8C). Consistent with these findings, increased IRE-1 phosphorylation and augmented XBP-1s expression were observed in the Pak2-T402E-Tg heart following 3 weeks of TAC (Figure 8D). Also, increased expression of GRP78, HRD-1, Derlin-3, Calreticulin, EDEM-1, Armet, and Hyou-1 was detected (Figure 8D). In addition, qPCR analyses showed that mRNA expression of key ERAD components (*Edem1* and *Hrd1*) was upregulated in Pak2-T402E-Tg heart after 3 weeks of TAC (Online Figure XIXA). However, the increased IRE-1 phosphorylation did not further enhance regulated IRE-1–dependent decay (RIDD) activity. qPCR analyses showed that the mRNA expression levels of 6 RIDD-specific substrates (*Blos1*, *Hgnat*, *Scara3*, *Pmp22*, and *Col6*) were comparable between Pak2-T402E-Tg and non-Tg mice (Online Figure XIXA). Moreover, expression and phosphorylation of PKB, ERK-1/2, and mTOR were comparable between Pak2-T402E-Tg and non-Tg mice (Online Figure XIXB). Finally, we tested whether Pak2 activation, via AAV-9–mediated gene delivery, could provide therapeutic benefits by promoting the protective ER stress response to prevent HF progression. C57BL/6 N mice undergoing TAC for 1 week were injected with TnT promoter-driven AAV-9-Pak2-T402E (1×10^11^ genomic particles) or AAV-9-GFP (as the control) under continuing TAC, and cardiac function was monitored on a weekly basis (Figure 8E; Online Figure XX). After 4 weeks of TAC, in the meantime as 3 weeks after injection, cardiac contractility of AAV-9-GFP injected mice became deteriorated (Figure 8F; Online Table III); therefore, subsequent analyses were taken at this time point. Excitingly, we found that AAV-9-Pak2-T402E injected hearts had significantly improved cardiac performance with reduced ventricular mass and apoptosis occurrence (Figure 8F through 8I; Online Figure XXI). Most noticeably, AAV-9-Pak2-T402E hearts demonstrated salutary ER stress responses, showing that IRE-1 phosphorylation and XBP-1 splicing levels were higher (Figure 8J). Also, protein expression of HRD-1, Derlin-3, Calreticulin, EDEM-1, and Hyou-1 was increased (Figure 8J). Compared with AAV-9-GFP hearts, which experienced ER dysfunction, transcript levels of key chaperones (*GRP78*, *ERO1*, and *PDI*) and ERAD components (*Edem1* and *Hrd1*) were enhanced (Figure 8K), whereas apoptotic molecules, CHOP and cleaved caspase-3 were lowered (Figure 8J). Taken together, data obtained from pressure overload models reveal the beneficial effects of Pak2 activation in promoting the protective ER stress response that prevents HF progression.

## Discussion

Secreted and membrane-bound proteins, representing ≈35% of total proteins, play critical roles in myocardial health and disease. These proteins undergo a coordinated process of synthesis, folding, secretion, and degradation in the ER.^24^ We discovered that Pak2 is abundantly expressed in close proximity to the ER membrane in cardiomyocyte. Directed by this clue, we have made novel discoveries in this study: (1) mice deficient of cardiac Pak2 exhibit a defective response to ER stress, contractile impairment and profound apoptosis; (2) Pak2 inactivation of PP2A is the molecular basis for stimulation of IRE-1/XBP-1–dependent UPR activation; (3) IRE-1 activator, Quercetin, and AAV-9–delivered XBP-1s relieve ER dysfunction in Pak2-CKO hearts; (4) Pak2 activation achieved by genetic overexpression or AAV-9–based approach is able to facilitate ER function, alleviate deleterious ER stress response, and therefore ameliorate HF progression (Figure 8L).

The serine/threonine kinase Pak2 is a member of the group I Pak family. Previous studies described a functional dichotomy of Pak2 in cell survival.^25,26^ Activation of full-length Pak2-stimulated cell survival, whereas the caspase-3–cleaved short form, Pak2-p34, promoted cell death.^27^ Since the studies in mouse fibroblasts and HEK293T used forced expression of Pak2-p34 or Pak2-D212N (caspase resistant form), it is not entirely clear whether Pak2-p34 has physiological functions. Instead, it may exist as a transient form before its degradation by the 26S proteasome. Functional evidence of a prosurvival role for Pak2 was first obtained in zebrafish, where removal of Pak2 led to hemorrhage because of autonomous endothelial cell defects.^28^ Further studies in mice reported that global deletion of Pak2 culminated in embryonic demise.^29^ Moreover, endothelial-specific deletion of Pak2 led to flawed blood vessel formation, which suggests the indispensable role of Pak2 in endothelial cell survival and angiogenesis.^30^ Akin to the previous study reporting Pak2 localization on the ER membrane of 293T and COS-7 cells,^31^ we discovered that a significant amount of Pak2 is expressed in close proximity to the ER membrane in cardiomyocytes. Moving forward, we unveiled an important protective role of Pak2 in pathologically stressed hearts for promoting UPR of the ER stress response. Greater hypertrophy in Pak2-CKO was likely a consequence of impaired UPR, because perturbation in ER homeostasis could cause accelerated ER-to-cytosolic Ca^2+^ efflux through the InsP3R (InsP3 receptor).^32^ Increased cytosolic Ca^2+^ concentration is apparently a master regulator in promoting hypertrophic remodeling.^33^ Worth mentioning, a previous study by Vettel et al^34^ reported that Pak2 was an integral component of Giβγ/PI3K (Gi protein beta gamma complex/phosphoinositide 3 kinase)-dependent prohypertrophic signaling. However, without functional evidence, their description of this signaling model in NRCMs using pharmacological inhibitors could not duly support Pak2 as a prohypertrophic factor in physiology and disease, because of the immature nature of NRCMs and lack of specificity in pharmacological agents.

Analogous to many stress-responsive signaling molecules, Pak2 loss under resting conditions did not cause any abnormality, implying that Pak2 may be dispensable for maintaining basal ER homeostasis in the heart. However, in the presence of superimposed stress, like tunicamycin or hemodynamic stress, Pak2 became activated to promote protective ER stress response and to accelerate protein folding to ensure protein quality control, resulting in relief of severe ER stress. This paradigm could explain why mice without cardiac Pak2 behaved normally under nonstressed conditions but failed to handle stress challenges.

On a relevant note to our previous studies, Pak1, a close family member of Pak2, was reported to protect the heart from pressure overload through positive regulation of JNK-NFAT (c-Jun N-terminal kinase-nuclear factor of activated T cells) pathway.^35^ Pak1 expression was also examined in this study, and it was abundantly expressed in the cytosol of cardiomyocytes, which is distinct from the expression pattern of Pak2. Although Pak2 expression is enriched at ER membranes, a small portion of cytosolic expression was observed, which indicates that Pak2 may have other functions apart from its role in promoting ER function. With regards of this assumption, future investigation is needed.

GRP78 is a key chaperone because it holds 3 UPR sensors at bay in the ER. Its dissociation from the UPR sensors is a hallmark of ER stress response. It is interesting to mention in our data that increased GRP78 by tunicamycin and decreased GRP78 by TAC was observed in Pak2-CKO hearts. This discrepancy in GRP78 expression is likely because of timing difference in ER stress response. Tunicamycin stimulation for 48 hours caused an acute ER stress response, where ER chaperones, including GRP78, were upregulated to ease the accumulation of aberrantly folded proteins. This upregulation was also discerned by the gene array in this study. The increase of GRP78 expression was augmented in the condition of Pak2 loss, which could be explained by the effect of ATF-6 for compensating the impaired action of XBP-1s. GRP78 is known to be subject to transcriptional regulation by both XBP-1s and ATF-6.^36,37^ On the contrary, 2 weeks of TAC caused a chronic ER stress response. In this sustained predicament, ATF-6 might be unable to compensate for faulty XBP-1s action. As a consequence, GRP78 was decreased and that indicated severe ER stress in Pak2-CKO hearts.

The highlight of this study is the dissection of the molecular basis for Pak2 facilitation of UPR during the protective ER stress response. Followed by extrapolation of gene array data, we postulated that Pak2 participation in ER protective action was via the IRE-1/XBP-1 branch of the ER stress response. IRE-1 activator, Quercetin, and AAV-9–delivered XBP-1s were able to relieve ER dysfunction in Pak2-CKO hearts, which provides functional evidence that establishes the Pak2-IRE-1/XBP-1 signaling cascade responsible for cardioprotection.

IRE-1 is the most fundamental ER stress sensor containing both a serine/threonine kinase domain and an endoribonuclease domain that regulates UPR conserved from yeast to humans.^7^ The unphosphorylated, inactive IRE-1 is a monomer and is bound to the chaperone GRP78.^38^ On the accumulation of unfolded/misfolded proteins in the ER, IRE-1 is triggered to dissociate from GRP78 and undergoes dimerization of its cytosolic kinase domain. This dimerization brings about the positioning of the cytoplasmic kinase domains in close proximity, which allows the trans-autophosphorylation of the kinase activation loop.^38^ IRE-1 phosphorylation is thus an important and necessary step for its RNase splicing activity, which leads to XBP-1 splicing, RIDD process, and activation of the UPR.^39^ Our data demonstrated that Pak2-stimulated IRE-1 phosphorylation, and the resulting activation of transcriptional factor XBP-1s upregulated expression of chaperones and ERAD components to relieve ER stress. As a consequence, clearance of α1-antitrypsin from the ER lumen was expedited. Conversely, Pak2 loss or its kinase-dead form failed to activate XBP-1 transcriptional activity. These findings further established that regulation of IRE-1/XBP-1 was dependent on Pak2 kinase activity. Although we did not obtain evidence that Pak2 directly interacted and then phosphorylated IRE-1, we discerned an action through PP2A inactivation to preserve IRE-1 phosphorylation at Ser724, a key event responsible for IRE-1 RNase activity. Further, we observed that Pak2 physically interacted with PP2Ac and affected its phosphorylation at tyrosine 307 (Tyr307) in response to ER stress, thereby inhibiting its phosphatase activity. PP2Ac knockdown or calyculin treatment was able to restore Pak2-knockdown–induced reductions in IRE-1 Ser724 phosphorylation and XBP-1 splicing. Of note, PP2Ac in vitro kinase assay showed Pak2 direct phosphorylation of PP2Ac. PP2Ac was known to contain 15 Ser sites and 18 Thr sites, however, which Ser/Thr site is subject to Pak2 catalysis is required for future investigations by proteomics analysis and site mutagenesis approach. In aggregate, the proposed action model is likely that Pak2 interacts and phosphorylates PP2Ac at Ser/Thr site(s), resulting in PP2Ac conformational shift that gives rise to permitting Tyr307 phosphorylation by receptor-associated tyrosine kinases or inhibiting its auto-dephosphorylation.^40^ Tyr307 phosphorylation leads to a reduction in PP2Ac phosphatase activity, consequently causing increased IRE-1 activation. In addition, PP2Ac binding with IRE-1 was observed in the unstressed condition, and this association was present under stress situation when Pak2 was lost. From multiple lines of evidence, it is reasonable to propose that initiation of IRE-1 autophosphorylation requires not only its dissociation from GRP78 but also inactivation of PP2A to prevent IRE-1 dephosphorylation. Pak2 appears as a negative regulator of PP2A for stimulating IRE-1 phosphorylation and subsequent XBP-1 splicing. In Pak2-CKO hearts under TAC or tunicamycin stimulation, Pak2 deficiency resulted in a loss of its inhibitory effect on PP2A. Consequently, IRE-1 failed to reach its optimal phosphorylation state to elicit UPR of the protective ER stress response. Thereafter, the ER stress response swiftly turned into a malicious process causing cardiomyocyte death, contractility impediment, and eventually, cardiac pathology progression to HF became inevitable. In accordance with this proposed action model, a previous study also reported that PP2A inactivation was required for IRE-1 activation in pancreatic cells through a mechanism involving RACK-1 (receptor of activated protein C kinase-1)–dependent scaffolding function.^21^

Another intriguing observation unveiled from this study was that both Pak2 loss and overexpression caused less fibrosis in TAC condition. This finding suggests that cardiac Pak2 may have a dichotomous action on ECM (extracellular matrix) remodeling. Because many ECM components are secreted proteins that are reliant on proper ER function, it would be very tempting to speculate that Pak2 regulation of ER function could be involved in ECM homeostasis in the heart. In addition, whether Pak2 activation could antagonize profibrotic pathways in the heart is currently unknown. Those assumptions require future investigations.

With regard to cardiomyocyte death in Pak2-CKO mice, distorted UPR and reduced ERAD were the apparent causality. Aberrantly folded proteins trapped in the ER lumen or in the cytosol cause proteotoxicity, which is featured as lumen damage and protein trafficking jam.^8^ Meanwhile, mounting CHOP level and leaky Ca^2+^ in the cytosol instigate multiple and inextricably linked prodeath cascades, ultimately triggering apoptosis and necroptosis.^10,41,42^ Of note, this Pak2-regulated cardioprotective effect which preserved ER function was verified in human cardiomyocytes. The human relevance of this regulatory mechanism forms a translational grounding to propose a therapeutic role for Pak2 in relieving ER dysfunction.

To test this possibility, we used 2 mouse models of Pak2 activation. Cardiac overexpression of active Pak2 allowed the mice to withstand offensive ER stress induced by 5 weeks of TAC. This beneficial effect of Pak2 prompted us to explore whether Pak2 activation has therapeutic value. In the therapeutic model, TAC-stressed C57BL/6N mice were injected with AAV-9–packed active Pak2. AAV-9-Pak2-T402E-injected mice had significantly improved cardiac performance and reduced apoptosis. Relief of ER stress in these mice significantly halted pathological progression from hypertrophy to HF. More interestingly, Pak2 activation did not cause adverse IRE-1–dependent RIDD activity. According to the structural analysis, IRE-1 contains multiple phosphorylation sites positioned at the linker region, kinase activation loop and RNase domain.^39^ Among these, Ser724, Ser726, and Ser729 at the kinase activation loop are responsible for IRE-1 autophosphorylation and subsequent RNase activity.^39^ Based on literature and our data, it is tempting to speculate that Pak2 stimulation of IRE-1 phosphorylation at its kinase activation loop via inhibition of PP2A activity is protective by virtue of XBP-1 splicing. Onerous ER stress could dampen Pak2 activation and induce other phosphorylation events at the linker region or RNase domain by heterokinases rather than autophosphorylation that may act as recruitment sites for RIDD substrates and TRAF (tumor necrosis factor receptor-associated factor) binding for ASK/JNK signaling, thereby gearing IRE-1 toward prodeath action. Indeed, enhanced phosphorylation of ASK and JNK was detected in TAC-stressed Pak2-CKO hearts in this study, suggesting that the selective action of IRE-1 for ASK and JNK phosphorylation is independent from Pak2-induced IRE-1 activation for XBP-1 signaling. To prove this hypothesis, several issues need to be investigated, including whether individual phosphorylation events influence dimer/oligomer formation and conversion, and how dimer/oligomer controls survival signal or death signal. Despite a dichotomy of IRE-1 activation in cell fate, IRE-1 global knockout was embryonically lethal and hepatic IRE-1 deletion caused liver stenosis,^43^ indicating a primary protective function of IRE-1 in physiology and disease. Similarly, a plethora of IRE-1 RNase inhibitors (eg, 4μ8C and STF-083010) are used for anticancer therapy because of their ability to induce apoptosis.^44^ Although functional evidence of IRE-1 in the heart is not available, a previous study in cardiac XBP-1s overexpression mice ascertained a protective role of XBP-1s in combating ischemia and reperfusion injury,^45^ which supports our data.

In conclusion, this study uncovers a new cardioprotective mechanism by Pak2 stimulation of the IRE-1/XBP-1 branch of UPR under ER stress conditions. The virtues of Pak2 activation provide the rationale for developing novel therapeutic strategies to treat ER dysfunction, a major pathological determinant underlying many forms of heart disease and HF in humans.

## Acknowledgments

We thank Andy Hayes and Leo Zeef (University of Manchester) for technical support on Affymetrix gene array; Aleksandr Mironov (University of Manchester) for technical support on transmission electron microscope; Rui-Ping Xiao (Peking University) for providing monkey heart tissue; and Gina Galli (University of Manchester) for proofreading.

## Sources of Funding

This study was supported by the British Heart Foundation (PG/12/76/29852, PG/14/71/31063, and PG/14/70/31039 to X. Wang and FS/15/16/31477 to W. Liu) and the National Institutes of Health (R01CA142928 and R01CA148805 to J. Chernoff).

## Disclosures

None.

## Supplementary Material

## Novelty and Significance

### What Is Known?

- Perturbation of endoplasmic reticulum (ER) homeostasis plays an important role in the development and progression of heart disease.
- Unfolded protein response activation is an adaptive mechanism to restore ER homeostasis in cardiomyocytes.
- Pak (p21-activated kinase)2 is a serine/threonine kinase, activated by Rac1 (Rac family small GTPase 1)/Cdc42 (cell division cycle 42), belonging to the group I Pak family members.
