# Supplementary material for: Pak2 as a Novel Therapeutic Target for Cardioprotective Endoplasmic Reticulum Stress Response
Source: Circ Res. 2019 Feb 28;124(5):696–711. doi: 10.1161/CIRCRESAHA.118.312829 (PMC6407830; doi:10.1161/CIRCRESAHA.118.312829)
Supplement: Supplementary file 1 [file res-124-696-s001.pdf]

# Supplemental Material

## Methods

All supporting data are available within the article.

### Animal models

All laboratory mice and rats in this study were maintained in a pathogen-free facility at the University of Manchester. Animal studies (mice and rats) were performed in accordance with the United Kingdom Animals (Scientific Procedures) Act 1986 and were approved by the University of Manchester Ethics Committee. Left ventricular tissue from rhesus monkeys with cardiac hypotrophy were provided by Prof. Rui-Ping Xiao (Peking University, China)[1]. Mice with the same genotype were randomly assigned to 1:1 allocation ratio to two groups subject to Sham or TAC surgery. We used online program (RANDOM.ORG) for randomization. We requested 1 set with 15 unique random animal ID, taken from the [1, 30] range. All *in vivo* studies were blinded for both genotype and surgical procedure/treatment during the measurement and analysis stages. Of note, only male mice aged between 10-12 weeks old were included in all *in vivo* experiments in this study.

### Generation of Pak2-Flox and Pak2-CKO mice

The Pak2-Flox mouse line was generated by the Cre-Loxp system with two LoxP elements flanking exon 2 of *Pak2*. Briefly, the two LoxP elements were inserted into intronic regions of the *Pak2* gene by homologous recombination in 129Sv-derived embryonic stem cells[2]. To generate cardiomyocyte-specific Pak2 knockout mice (Pak2-CKO), Pak2-Flox mice were mated with mice expressing Cre under α myosin heavy chain (αMHC) promoter[3]. Specific deletion of the *Pak2* gene in cardiomyocytes was verified by PCR of genomic DNA using (5'-ATCTTCCCAGGCTCCTGACT-3') and (5'-TGAAGCTGCATCAATCTATTCTG-3') primers. Thereafter, Pak2-Flox and Pak2-CKO mice were backcrossed into a C57BL/6J background for 8 generations. Pak2-CKO mice, along with Pak2-Flox littermates as controls, were used to investigate the specific impacts of Pak2 deletion in the heart.

### Genetic manipulation of Pak2 cDNA

Human Pak2 cDNA (Source Biosciendce, NCBI Accession # BC069613) was sub-cloned into p3xFlag-CMV-7.1 vector (Sigma). Single amino acid changes were achieved by using QuickChange Site-Directed Mutagenesis Kit (Stratagene, 200518). The auto-phosphorylation site Thr402 of Pak2 was mutated to glutamic acid (T402E) to achieve constitutively active Pak2 using the primers 5'-AGAGCAAAGGCAGTGAGATAATCGGAACGCC-3' and 5'-TCTCGTTTGCGTCACTCTATTAGCCTTGCGG-5'), whereas Thr402 was mutated to alanine (T402A) to obtain kinase dead Pak2 using the primers 5'-GCAGAGCAAACGCAGTGCCATGGTCGGAACGCCAT-3' and 5'-ATGGCGTTCCGACCATGGCACTGCGTTTGCTCTGC-5' following the manufacturer's instructions. Both Pak2 (T402E) and Pak2 (T402A) were used for generating transgenic mouse model, adeno-associated virus or adenovirus.

### Generation of Pak2 cardiac overexpression mice (Pak2-T402E-Tg)

To generate the Pak2 cardiac overexpression mouse model, Flag-tagged constitutively active Pak2 (T402E) was sub-cloned into αMHC promoter-driven vector. The transgenic mice were achieved by embryo microinjection of the plasmid into FVB mice. The ratio of Pak2 transgene copy number was determined by real-time PCR using the primers 5'-ATGACAGACAGATCCCTCCT-3' (sense) and 5'-GCTAAAGATGGTGCTGCTCA-3' (antisense). qPCR was used to determine copy

numbers of transgenen, and the line 3.22 was chosen because of its low transgene copy numbers. The line 3.22 was set up for breeding with C57BL/6J mice, and the ectopic Pak2 expression in the hearts of its offspring was examined by Western blotting, which showed approximately 4 times of that in non-Tg mice. Thereafter, offspring of line 3.22 were crossed with C57BL/6J mice for 6 generations and used as a gain-of-function model in this study for study of Pak2 function in the heart.

**Construction of adeno-associated virus-Pak2 (AAV9-Pak2-T402E)**

The pSSV9-TnT-eGFP plasmid was modified by replacing GFP with Flag-tagged constitutively active Pak2 (T402E) cDNA to construct the pSSV9-TnT-Pak2 plasmid. To obtain the recombinant AAV9-Pak2 virus and the control AAV9-eGFP virus, HEK293T cells were co-transfected with an adenoviral helper plasmid (pDGΔVP), pAAV2-9 Rep-Cap plasmid (p5E18-VD2/9) and the adeno-associated virus genome plasmids pSSV9-TnT-Pak2 and pSSV9-TnT-eGFP, respectively. Afterwards, recombinant AAV9-Pak2 was purified by discontinuous iodixanol gradient and titrated by quantitative polymerase chain reaction (qPCR) relative to a standard curve[4]. To overexpress Pak2 in cardiomyocytes, the mice were anesthetized with 2% isoflurane one week after TAC and $1 \times 10^{11}$ genomic particles were administered by intravenous injection via jugular vein. AAV9-GFP was injected as the control. Three weeks after injection, cardiac function was analyzed by echocardiography and hearts were collected for histological and biochemical analyses.

**Construction and administration of AAV9-XBP1s**

To obtain functional evidence for supporting the underlying mechanism that Pak2 regulates cardiac ER response, we used a genetic approach to determine whether overexpression of XBP1s is able to rescue cardiac dysfunction in Pak2-CKO heart after TAC. The pSSV9-TnT-eGFP plasmid was modified by replacing GFP with human Flag-tagged spliced XBP1 (XBP1s) cDNA (ID 63680, Addgene) to construct the pSSV9-TnT-XBP1s plasmid. To obtain the recombinant AAV9-XBP1s virus, HEK293T cells were co-transfected with an adenoviral helper plasmid (pDGΔVP), a pAAV2-9 Rep-Cap plasmid (p5E18-VD2/9) and an adeno-associated virus genome plasmid (pSSV9-TnT-XBP1s). Next, the AAV9-XBP1s virus was purified by a discontinuous iodixanol gradient and titrated by qPCR. Pak2-CKO mice were subject to TAC followed by intravenous injection of $1 \times 10^{11}$ viral particles. AAV9-GFP was injected as the control. After two weeks, cardiac function was analyzed by echocardiography and the hearts were collected for histological and biochemical analyses.

**Transverse aortic constriction (TAC)**

Male (8-10 weeks old) mice were anesthetized with Ketamine (100 mg/kg) and Xylazine (5 mg/kg) by intraperitoneal injection and ventilated for TAC surgery as previously described[5]. Briefly, the transverse aorta between right innominate and left common carotid arteries was subjected to a 27-gauge constriction by a 7-0 Prolene suture to produce an approximate pressure difference of 30-40 mmHg between the two common carotid arteries. Buprenorphine (0.1 mg/kg) was subsequently administered for post-operative analgesia. Cardiac function was detected by echocardiography and the hearts were collected for further analyses at different time points after TAC. Based on our surgical procedure (approved by the UK home office), 20% mortality occurs in TAC procedure despite genetic modification background. All survival mice were included in the study. When Pak2-CKO mice were subjected to pressure overload, their cardiac function became very poor after 2 weeks TAC, therefore they were terminated for functional and histological analysis,

and cell death detection. Signaling changes occurred prior to functional impairment, therefore we chose 1 week TAC as the time point to evaluate ER stress signaling pathways. For Pak2-T402E-Tg, we challenged the mice aggressively to 5 weeks TAC to evaluate beneficial effects of Pak2 on cardioprotection, during this time point Pak2-T402E-Tg mice were still resilient to pressure overload. To assess changes in ER signaling, 3 weeks TAC was an appropriate time point to distinguish differences between Pak2-T402E-Tg and non-Tg mice.

**Tunicamycin administration**

To examine the effect of Pak2 on ER stress responses in the heart, a single dose (2 mg/kg) of tunicamycin (Sigma, T7765) or saline was applied to Pak2-Flox mice or Pak2-CKO mice by intraperitoneal injection. 48 hours after the injection, cardiac function was examined and the hearts were collected for further analyses.

**Tauroursodeoxycholic acid (TUDCA) administration**

To determine the ability of a small chemical chaperone TUDCA to relieve Pak2 loss induced-ER dysfunction in a pressure-overloaded heart, on the second day after operation, TAC- or sham-operated Pak2-CKO mice were subjected to intraperitoneal injection of TUDCA (300 mg/kg/day, Sigma, T0266) daily for 2 weeks. Cardiac function was detected and the hearts were collected for further analyses.

**Quercetin administration**

In order to determine whether pharmacological activation of IRE1 is able to relieve ER dysfunction induced Pak2 loss and pressure overload stress, flavonoid Quercetin[6] (IRE1 activator, 15mg/kg/day, in 0.5% methylcellulose, Q4951, Sigma) was administered via oral gavage daily for 2 weeks to Pak2-CKO mice on the second day after TAC. Cardiac function was examined and the hearts were collected for further analyses.

**Echocardiography**

Mice were terminally anesthetized with 2,2,2-Tribromethanol (Avertin, 200mg/kg, Sigma) and echocardiographic assessment of cardiac function was carried out. Transthoracic M-mode echocardiographic recordings were performed using an Acuson Sequoia C256 system (Siemens) ultrasound machine[5]. For each mouse, measurements of the left ventricle end-systolic (LVESD) and end-diastolic (LVEDD) dimensions, end-diastolic posterior wall thickness (dPW), and fractional shortening (FS%) were measured or calculated.

**Histology and TUNEL Assay**

5µm-thick paraffin embedded heart sections were stained with hematoxylin & eosin or TdT-mediated dUTP nick end labeling (TUNEL) assay as described[5]. Cardiomyocytes were marked with anti-α-actinin (Sigma, A7811) and anti-Connexin43 antibody (Sigma, C6219). Hematoxylin & eosin staining was performed to measure cross-sectional areas by randomly selecting 200 cardiomyocytes. TUNEL assay was performed to detect apoptotic cardiomyocytes using the in situ Cell Death Detection kit (Roche). A total of 10,000 cardiomyocytes from random fields per heart were analyzed. The images were obtained by Zeiss Axioplan2 microscope and then analyzed by Image J software.

**Transmission electron microscopy**

Heart samples were fixed overnight in 0.1 M HEPES buffer (pH 7.2) containing 4% formaldehyde and 2.5% glutaraldehyde, and then post-fixed in 0.1 M cacodylate buffer (pH 7.2) with 1% osmium tetroxide and 1.5% potassium ferrocyanide for 1 h, followed by 1% tannic acid in 0.1 M cacodylate buffer (pH 7.2) for 1 h, finally in 1% uranyl acetate for 1 h. Subsequently, the samples were dehydrated in ethanol, embedded in TAAB 812 resin and polymerized for 24 h at 60 °C. Sections were cut

with Reichert Ultracut ultramicrotome and examined with FEI Tecnai 12 Biotwin microscope at 100 kV accelerating voltage. Images were taken with Gatan Orius SC1000 CCD camera.

**Neonatal rat cardiomyocytes (NRCMs) isolation and culture**

Neonatal rat cardiomyocytes (NRCMs) were isolated from 2 days old Sprague Dawley rats using a standard enzyme solution (30 U/100 ml collagenase A, 100 mg/ml pancreatin, NaCl 116 mM, HEPES 20 mM, $NaH_2PO_4$ 1 mM, glucose 6 mM, KCl 5 mM, $MgSO_4$ 0.8 mM, pH 7.4) as previously described[5]. NRCMs were cultured in cardiomyocyte maintenance medium (79.5% DMEM, 19.5% M199, 1% FBS, 1% penicillin-streptomycin, 2.5 µg/ml amphotericin-B and 1 µm bromodeoxyuridine). Rat H9C2 cardiomyoblasts and HEK293T cells were maintained in Dulbecco's modified Eagle medium (DMEM) supplemented with 10% fetal bovine serum (FBS) along with penicillin (100 U/mL) and streptomycin (100 µg/mL). Tunicamycin (5 µg/mL) or thapsigargin (0.5 µM, Sigma) were applied to induce ER stress.

**Immunofluorescent staining of NRCMs**

To examine the cellular localization of molecules in cardiomyocytes, NRCMs were seeded into sterile coverslips pre-coated with laminin (1µg/cm$^2$). Afterwards, cells were fixed with 4% paraformaldehyde, followed by blocking and permeabilization with 3% BSA and 0.1% Triton-X solution. Fixed cells were incubated with various primary antibodies (1:200 dilution) against PKB (Cell Signaling, 9272), ERK5 (Cell Signaling, 3372), ERK1/2 (Cell Signaling, 9102), JNK (Cell Signaling, 9252) or p38 (Cell Signaling, 9212), and Calnexin (ER marker, Santa Cruz, sc-6465), respectively. Secondary anti-rabbit antibody (Invitrogen, 1:500) conjugated to Alexa Fluor®594 and anti-goat antibody (Invitrogen, 1:500) conjugated to Alexa Fluor®488 were used to detect the expression of the respective proteins, whereas DAPI was used for nuclear visualization. Images were collected on an Olympus BX51 upright microscope and captured using a Coolsnap ES camera (Photometrics) through MetaVue Software (Molecular Devices). To exclude the possibility of false positive by the primary antibodies, IgG (Cell Signaling, 2729) was used as the control antibody. IgG did not detect any positive staining, which was not represented.

**Adult rat cardiomyocytes (ARCMs) isolation and culture**

Six weeks old, male, Wistar rats (150g) were sacrificed by intraperitoneal injection of 750µl Pentobarbitpone Sodium 20% w/v (Pentoject) and 250µl Heparin Sodium (1000 U/ml, Wockhardt UK). The heart with 2mm aorta were excised and placed in an ice-cold perfusion buffer containing heparin (Hank's balanced salt solution, HBSS, Sigma), 10mM Butandione monoxime, 11mM glucose, 1mM $MgSO_4$; 10% heparin). Next, the heart was cannulated and mounted on a Langendorff perfusion apparatus and perfused with calcium-free digestion buffer containing Collagenase type II (65 U/ml, Worthington Biochemical) and protease from Streptomyces Griseus (0.28 U/ml, Sigma) for 20 minutes. After perfusion, the heart was carefully transferred into trypsin solution (4.4 U/ml Trypsin, Worthington; 70 U/ml DNase I, Worthington) and sliced into small pieces. 15ml of digestion buffer (115U/ml Collagenase type II, Worthington; 0.14 U/ml Protease from Streptomyces Griseus (Sigma), 4.4 U/ml Trypsin, Worthington and 70 U/ml DNase I, Worthington in perfusion buffer) was added and incubated in a 37$^o$C water bath for 20 minutes. In order to remove remaining non dissolved tissue, the cell suspension was passed through a 100µm filter (Corning). The filtered cell suspensions were centrifuged at 1200rpm for 3 minutes and the cell pellet was washed twice with wash buffer (1:1 perfusion buffer and ACCT media consisting in low glucose, GlutaMAX™,

pyruvate, no HEPES DMEM, 1g BSA, 5mM L-carnitine, 2mM Creatine, 5mM Taurine and 100U/ml penicillin/1mg/ml streptomycin). Afterwards, the cell suspension was slowly pipetted on top of a BSA solution (1mM BSA in ACCT media) and left to pass through the gradient for 40 minutes to remove dead cells and non-cardiomyocytes. The resultant cells were resuspended in ACCT media containing blebbistatin (20μM, Abcam) and plated on plates coated with Geltrex (Gibco, LDEV-Free, hESC-qualified Reduced Growth Factor Basement Membrane).

**Immunofluorescent staining of ARCMs**

In order to examine the cellular localization of Pak1 and Pak2 in cardiomyocytes, ARCMs were seeded onto sterile coverslips pre-coated with Geltrex. After treatment with tunicamycin (5μg/ml) for 2 hours, ARCMs were fixed in 4% PFA. Next, cells were permeabilized in 0.2% triton X-solution and blocked with 10% normal donkey serum (Stratech). Fixed ARCMs were incubated with primary antibodies (1:200 dilution) against Pak2 (Cell Signaling, 2608) or Pak1 (Cell Signaling, 2602) combined with Calnexin (ER membrane marker, Santa Cruz, sc-6465), PDI (ER lumen marker, Abcam, ab2792), GM130 (Golgi marker, Abcam, ab169276), Ryanodine receptor (SR marker, Sigma, R128) or CoxIV (mitochondrial marker, Abcam, ab33985). Alternatively, for mitochondria detection, cells were incubated with MitoTracker Red (M22425, ThermoFisher Scientific) for 30 minutes. Secondary anti-rabbit antibody (Stratech, 1:1000) conjugated to Alexa Fluor®488, anti-goat antibody (Stratech, 1:1000) conjugated to Alexa Fluor®594 or anti-mouse antibody (Stratech, 1:1000) conjugated to Alexa Fluor®594 were used to detect the aforementioned proteins, whereas DAPI was used for nuclear visualization. Images were collected on a Leica TCS SP8 AOBS upright confocal microscope. To exclude the possibility of false positive by the primary antibodies, IgG (Cell Signaling, 2729) was used as the control antibody. IgG did not detect any positive staining, which was not represented.

**Transfection of H9C2 and NRCMs**

2 μg cDNA plasmids Pak2-T402E, Pak2-T402A, Myc-GRP78 (Addgene, gene ID #3309) or V5-IRE1 (Source Bioscience, gene ID #2081)) were transfected in H9C2 or NRCMs using Lipofectamine 2000 reagent (Invitrogen) to investigate luciferase reporter activity or protein association, while siRNAs (100 nM) were transfected in NRCMs using Lipofectamine Plus reagent (Invitrogen) according to the manufacturers' instructions. Rat IRE1α siRNA (siIRE1, gene ID #498013, si genome SMART pool) and rat XBP1 siRNA (siXBP1, gene ID #289754, si genome SMART pool) were purchased from Dharmacon; rat PP2Ac siRNA (siPP2Ac, gene ID #24672) was purchased from Thermo Fisher Scientific. Negative control siRNA (siScr, AGGUAGUGUAAUCGCCUUG) was obtained from Sigma.

**Adenoviral production and infection**

SureSilencing shRNA plasmids were purchase from Qiagen, the negative control rat shRNA (GGAATCTCATTCGATGCATAC) and rat shPak2 (GGAGTTAAAGAATCCCAACAT) plasmids were generated and packed into adenovirus (Ad-shCon and Ad-shPak2) using the BLOCK-iT adenoviral RNAi expression system (Invitrogen). To aid virus uptake, replication and packaging in cells, the DNA plasmids were digested with PacI restriction endonuclease to expose two inverted terminal repeats (ITRs) to aid virus uptake, replication and packaging in cells. Human Embryonic Kidney 293T (HEK293T) cells were transfected with the purified plasmids. Cells were harvested once 80% cytopathological effect (CPE) was observed. Three cycles of freeze and thawing

were carried out to release the virus particles. Adenovirus was collected by centrifugation in gradient caesium chloride (CsCl) (1.33g/ml and 1.45g/ml). Afterwards, the band containing the adenovirus was removed and purified using a dialysis tubing (pore size 24 Angstrom, Medicell). Adenovirus infection was applied at 25 multiplicity of infection (MOI).

**Luciferase reporter assay**

To determine whether Pak2 regulates the activity of Hrd1 promoter, NRCMs were co-transfected with Pak2 (T402E) or Pak2 (T402A), and human Hrd1 promoter luciferase reporter[7] (-3324 to -1 relative to transcription start site, containing XBP1s specific UPRE sites, kindly provided by Professor Kazutoshi Mori, Kyoto University, Japan) for 48 h. To determine Pak2 regulation of Hrd1 activity through IRE1, IRE1 cDNA and Hrd1 promoter luciferase reporter was co-transfected in the Ad-shPak2-infected NRCMs with or without tunicamycin (5µg/mL, Sigma) treatment for 2 h. Thereafter, the luciferase activity was analyzed using Dual-Luciferase Reporter Assay System (Promega, E1910) according to the manufacturer's instructions.

**Cycloheximide chases**

NRCMs were co-transfected with plasmids expressing a mutant protein of α1-antitrypsin[8] (α1ATmut, an ER luminal misfolded protein, kindly provided by Professor Kazutoshi Mori, Kyoto University, Japan) and Pak2 (T402E), respectively. 48 hours after the transfection, 100 µg/mL of cycloheximide was added, and chase experiments were performed in a time window of 4-8 hours after addition of cycloheximide to examine the effect of active Pak2 in the degradation rate of α1-ATmutant[9]. Changes of α1-AT levels were assessed by immunoblot.

**Immunoprecipitation**

To investigate protein association, immunoprecipitations were performed with Protein G sepharose (Sigma) following the manufacturer's instruction. Briefly, NRCMs or H9C2 cells were lysed with immunoprecipitation buffer (Tris 50 mM, NaCl 250 mM, 0.25% v/v TritonX- 100, and 10% Glycerol; pH 7.4). 2 mg of the protein extract was immunoprecipitated with antibodies against Pak2 (Cell Signaling, 2608), Flag (Cell Signaling, 2368), V5 (Cell Signaling, 13202), Myc (Cell Signaling, 2272), or IgG (Cell Signaling, 2729), respectively. Immune complexes were eluted in Laemmli sample buffer (65.8 mM Tris-HCl, pH 6.8, 2.1% SDS, 26.3% glycerol, 0.01% bromophenol blue). Precipitated and input proteins were subjected to SDS-PAGE and immunoblotted using the respective antibodies.

**PP2Ac and PP2Ce phosphatase activity assay**

PP2Ac and PP2Ce activity was determined using the immunoprecipitation phosphatase assay kit (Millipore, 17-313). H9C2 cells were lysed with a buffer containing 20 mM imidazole-HCl, 2 mM EDTA, 2 mM EGTA, pH 7.0, with 10 µg/mL each of aprotinin, leupeptin, 1 mM benzamidine, and 1 mM PMSF. Following sonication at an amplitude of 10 microns for 10 seconds using a Soniprep 150 (MSE, London, UK), the resulting fractions were incubated with 4 µg of either the antibody against PP2Ac (Millipore, 05-421) or PP2Ce (PPM1L, Thermo Fisher, MA5-24320) and protein A-agarose at 4 °C for 2 hours. Afterwards, the immunecomplexes were mixed with 750µM threonine phosphopeptide (Millipore, 12-219) for phosphatase reaction. Free phosphate concentration was detected by incubating with Malachite Green Solution and the absorbance was read at 630 nm in a plate reader (Synergy HT). As an internal control, the reaction's specificity was assessed by inhibition of PP2Ac activity using 10 nM Calyculin (a PP2A inhibitor) before titration. Alternatively, samples were incubated with an antibody against human IgG (Cell Signaling, 2729)

as a negative control for assessing PP2Ce activity. The phosphatase activity was expressed relative to the untreated control free phosphate in each reaction by equal amount of proteins.

**Cell Viability**

Cardiomyocytes cell viability was determined by MTT [3-(4,5-dimethylthiazol-2-yl)-2,5-diphenyltetrazolium bromide] reduction assay. After treatment, cells were incubated in culture media containing MTT solution (0.5 mg/mL) for 3 hours at 37°C. The purple formazan product in the cells was solubilized by addition of acidic isopropanol (0.04 M HCl) and the absorbance was measured at 570 nm with background subtraction at 630nm. Cell viability was expressed as the percentage of untreated controls.

**Human induced pluripotent stem cell (iPSC)-derived cardiomyocytes**

Human iPSCs were derived from peripheral blood cells[10], and were maintained in feeder-free culture conditions with E8 medium (Thermo Fisher Scientific) on Geltrex-coated plates. Standard directed cardiomyocyte differentiation of iPSCs was initiated at confluence of 90-100% via Wnt signaling modulation using cardiac differentiation medium (RPMI1640 HEPES Glutamax, 500 mg/L human recombinant albumin, 200 mg/L Lascorbic acid 2-phosphate) and progressive treatment was applied with 4 µM CHIR99021 (inhibitor of Glycogen synthase kinase 3) for 48h and 5 µM IWP2 (Wnt Inhibitor) for further 48h. Medium was changed to cardiac culture medium (RPMI1640 HEPES Glutamax, B27) at day 10. Cardiomyocytes were enriched using cardiac selection medium (RPMI 1640 minus Glucose, 4 mM Lactate, 500 mg/L human recombinant albumin, 200 mg/L L-ascorbic acid 2-phosphate) for 5 days. iPSC-derived cardiomyocytes (iPSC-CMs) were cultured in cardiac culture medium up to 120 days for maturation. To examine ER stress pathway, iPSC-CMs were transfected with human Pak2 siRNA (gene ID #5062, si genome SMART pool, 100 nM, Dharmacon) using Lipofectamine Plus reagent (Invitrogen) according to the manufacturers' instructions, followed by 6 hours treatment of tunicamycin (2 µg/mL) and TUDCA (200 µg/mL) as indicated in Results.

**Lysate preparation and immunoblotting**

Total protein from tissues or cells was obtained with Triton lysis buffer (Tris 20 mM, NaCl 137 mM, EDTA 2 mM, 1% Triton X-100, β-glycerophosphate 25 mM, $Na_3VO_4$ 1 mM, phenylmethanesulfonylfluoride 1 mM, aprotinin 1.54 µM, leupeptin 21.6 µM, 10% glycerol; pH 7.4). Protein concentration was determined by Bio-Rad protein assay. Protein extracts (30 µg) were subject to immunoblot analyses with antibodies (all antibodies applied in this study were used as 1:1000 dilution) against Pak2 (Cell Signaling, 2608), Pak1 (Cell Signaling, 2602), Pak3 (Cell Signaling, 2609), CHOP (Cell Signaling, 2895), cleaved caspase-3 (Cell Signaling, 9661), PP2Ac (Cell Signaling, 2038), phospho-Pak2 (Cell signaling, 2601), phospho-PERK (Cell Signaling, 3179), Flag (Cell Signaling, 2368), Myc (Cell Signaling, 2272), IRE1 (Abcam, ab37073), GRP78 (Abcam, ab21685), ATF6 (Abcam, ab37149), phospho-IRE1 (Abcam, ab48187), XBP1s (Cell Signaling, 12782), phospho-PP2Ac (Santa Cruz, 271903), Alpha-1-Antitrypsin (Bethyl, A80-122A), ATF4 (Cell Signaling, 11815), PKB (Cell Signaling, 9272), phosphor-PKB (Cell Signaling, 9271), ERK1/2 (Cell Signaling, 9102), phosphor-ERK1/2 (Cell Signaling, 9101), JNK (Cell Signaling, 9252) phosho-JNK (Cell Signaling, 9251), Armet (Abcam, ab67271), p38 (Cell Signaling, 9212), phosphor-p38 (Cell Signaling, 4631), mTOR (Cell Signaling, 2983), phosphor-mTOR (Cell Signaling, 2971), HRD1 (Novus, NB100-2526), Ero1 (Cell Signaling, 3264), Calreticulin (Thermo Fisher Scientific, PA3-900), Derlin3 (Sigma, D2194), eIF2α (Cell

Signaling, 9722), phosphor-eIF2α (Cell Signaling, 9721), PDI (Abcam, ab2792), ASK1 (Cell Signaling, 3762), phosphor-ASK1(Cell Signaling, 3765), EDEM1 (Abcam, ab55920), HYOU1 (Cell Signaling, 13452), PP2Ce (Thermo Fisher Scientific, MA5-24320), ubiquitin (Cell Signaling, 3936) and Tubulin (Sigma, T6199). Immune-complexes were detected by enhanced chemiluminescence with anti-mouse, anti-rabbit or anti-goat immunoglobulin-G coupled with horseradish peroxidase.

**Fractional protein extraction**

Fractional proteins were extracted with gradient centrifugation from ARCMs and heart tissue samples. Briefly, tissue samples were lysed in cytoplasmic buffer (Tris 20 mM, NaCl 10 mM, $MgCl_2$ 2 mM, EDTA 2 mM, β-glycerophosphate 25 mM, $Na_3VO_4$ 1 mM, PMSF 1 mM, Aprotinin 1.54 μM, and Leupeptin 21.6 μM; pH 7.4). The lysates were spun at 2,000g for 10 minutes in order to remove cytoplasmic proteins. The pellets were re-suspended in the nuclear buffer (Tris 50 mM, NaCl 500 mM, EDTA 2 mM, 10% Glycerol, 1% TritonX-100, β-glycerophosphate 25 mM, $Na_3VO_4$ 1 mM, PMSF 1 mM, Aprotinin 1.54 μM, and Leupeptin 21.6 μM; pH 7.4), followed by centrifugation at 10,000g for 10 minutes, leaving the nuclear proteins suspended in the supernatant. The cytoplasmic portion was spun at 100,000g for 2 hours. The supernatant was collected as cytosolic protein portion, whereas the pellets were re-suspended using Triton lysis buffer. Followed by further centrifugation at 10,000g for 20 minutes, the supernatant was collected as organelle fraction, including proteins from mitochondria, ER, and Golgi. Protein concentration was determined by Bradford assay (Bio-Rad). The antibody against CoxIV (a mitochondria marker, Abcam, ab16056), PDI (an ER marker, Abcam, ab2792), GM130 (a Golgi marker, Abcam, ab169276), GAPDH (cytosolic protein, Abcam, 9482), and H3 (nuclear protein, Cell Signalling, 9715) were used for immunoblotting analyses.

**Measurement of Pak2 kinase activity**

Ventricular samples were homogenised in Triton lysis buffer. Protein extracts were precipitated with a polyclonal antibody against to Pak2 (Santa Cruz, sc-373740) and protein A agarose beads (Cell Signalling) overnight. The precipitation was washed in kinase buffer (25 mM HEPES, pH 7.4, 25 mM ß-glycerophosphate, 25 mM MgCl2, 2 mM dithiothreitol, 0.1% orthovanadate). Afterwards, the kinase reactions were performed by incubation with glutathione S-transferase (GST)-MEK1 (the substrate for Pak2, Merck, 475702) at 30°C for 30 minutes in a kinase buffer containing 50μM ATP. The reactions were terminated by the addition of Laemmli sample buffer. Pak2 kinase activity was measured by the detection of phosphorylated MEK1 using an antibody against phospho-MEK1(S298) (Cell Signalling, 9128) with SDS-PAGE immunoblotting.

***In vitro* kinase assay**

To study whether Pak2 directly phosphorylates PP2Ac, *in vitro* Pak2 kinase assay was conducted using recombinant PP2Ac as the substrate. H9C2 cells overexpressing Pak2-T402E were lysed in Triton lysis buffer. Protein extracts were precipitated with anti-Flag antibody (Cell Signaling, 2368) followed by incubation with protein A agarose beads overnight. The precipitation was washed in kinase buffer (25 mM Tris, 25 mM β-glycerophosphate, 10 mM $MgCl_2$, 2 mM dithiothreitol, 0.1% orthovanadate, aprotinin 1.54 μM, leupeptin 21.6 μM, pH 7.5). Next, the kinase reaction was carried on by incubation of the precipitation with glutathione S-transferase-PP2Ac (Sigma, SRP5336) at 30°C for 30 minutes in kinase buffer containing 200 μM ATP. The amount of phosphorylated PP2Ac was determined

using the universal fluorometric kinase assay kit (Abcam, ab138879). In detail, 20 µl of the kinase reaction mixture was then added with 20 µl of ADP sensor buffer and 10 µl of ADP sensor for a further incubation for 15 min. The amount of phosphorylated PP2Ac was measured by monitoring the fluorescence intensity with a fluorescence plate reader (Synergy HT) at Ex/Em = 530 /590 nm. An ADP standard curve was also performed and used as a reference to determine ADP production from samples. The amount of phosphorylated PP2Ac was finally represented with ADP concentration produced by kinase reaction.

**Construction of ER-Pak2-KDEL and Ribophorin-Pak2 plasmids**

To evaluate whether Pak2 is localized in the ER lumen, Pak2-T402E (kinase active form) and Pak2-T402A (kinase dead form) were constructed into pEF/Myc/ER-Crimson vector (Addgene, 38770) containing an N-terminal ER signal (MGWSCIILFLVATATGAHS) and the lumen target peptide sequence KDEL at its C-terminal region. The SalI restriction site within Pak2-T402E and Pak2-T402A cDNA was mutated using QuickChange Site-Directed Mutagenesis Kit (Stratagene, 200518) and the primers 5'-CTTATGGCCCTAAAGTTGACATATGGTCTCTGG-3' and 5'-CCAGAGACCATATGTCAACTTTAGGGCCATAAG-3, which did not alter the respective amino acid encoded. Afterwards, the PCR products amplified using the primers 5'-ACGCGTCGACATGTCTGATAACGGAGAACT-3' and 5'-ATTTGCGGCCGCACGGTTACTCTTC-3' was digested by SalI and NotI followed by subcloning into pEF/Myc/ER-Crimson vector to generate pEF/Myc/ER-Pak2 (T402E)-KDEL and pEF/Myc/ER-Pak2 (T402A)-KDEL.

To evaluate whether Pak2 is localized in the close proximity to the ER membrane, human Ribophorin I ER transmembrane domain was fused to the N-terminus of Flag-Pak2-T402A[11]. Ribophorin I-Pak2-T402A was constructed from p3xFlag-CMV-7.1-Pak2-T402A and pcDNA5FRT-Ribophorin1V5 using overlap extension PCR technique. In detail, nucleotides 1 to 1395 of Ribophorin I (encoding Ribophorin I ER transmembrane domain) was amplified using the primers 5'-CGGGGTACCGCCACCATGGAGGCGCCAGCCGCCGGC-3' and 5'-GGTCTTTGTAGTCCATCTTGGTGATGGAGAAG-3'. Meanwhile, Pak2-T402A was amplified using the primers 5'-CTTCTCCATCACCAAGATGGACTACAAAGACC-3' and 5'-CGGGATATCTTAACGGTTACTCT-3'. Subsequently, the two PCR products were fused in a second round of PCR using the primers 5'-CGGGGTACCGCCACCATGGAGGCGCCAGCCGCCGGC-3' and 5'-CGGGATATCTTAACGGTTACTCT-3'. The final PCR product was digested by KpnI and EcoRV and inserted into pcDNA5 to gain Ribophorin I-Pak2-T402A.

**Affymetrix Gene Expression Array**

Total RNA was extracted from heart tissue using Trizol reagent (Invitrogen). RNA quality was checked using an Agilent 2100 Bioanalyser (Agilent Technologies). Hybridization cocktail was hybridized to Mouse Genome 430 2.0 arrays (Affymetrix) according to manufacturer's instructions. Arrays were read using Agilent GeneArray scanner 3000 7G using Affymetrix GCOS (V1.4) software. Technical quality control and outlier analysis was performed with dChip (V2005) using the default settings[12]. Background correction, normalization and gene expression analysis were performed using RMA in Bioconductor[13]. An expression diagram of selected genes was generated by normalizing to Pak2-Flox group treated with tunicamycin (in log scale the mean of Pak2-Flox group was subtracted from each expression value).

**Detection of XBP1 mRNA splicing**

To examine the activation of IRE1/XBP1 pathway, total RNA was obtained from left ventricular tissues or cells using Trizol (Invitrogen) followed by conversion to cDNA. PCR were performed using the primers 5′-AAACAGAGTAGCAGCTCAGACTGC-3’ (sense) and 5′-TCCTTCTGGGTAGACCTCTGGGA-3 (antisense) to detect both un-spliced and spliced isoforms of XBP1. Subsequently, electrophoresis in 2% Agarose gels revealed the inactive XBP1 as the unspliced form with 289 bp and the active XBP1 as a spliced form with 263 bp.

**Quantitative real-time polymerase chain reaction**

Total RNA was extracted from ventricular tissues or cells using Trizol (Invitrogen) followed by conversion to cDNA. All primers were purchased from Qiagen. Real-time quantitative PCRs were performed using SYBR Select PCR Master Mix according to the manufacturer’s instruction in the Step One Plus PCR System (Applied Biosystems). The fold change was analyzed using the $2^{-\Delta\Delta}$CT method[14]. The level of expression was normalized to *Gapdh*.

**Data Analysis**

Sample sizes were calculated based on available comparable data to achieve 90% power at a 5% significance level. For all *in vivo* and *in vitro* analyses, normal distribution (Gaussian distribution) was first determined by Shapiro–Wilk test. Data were then analyzed using Student’s t-test for comparisons between two experimental groups or one- or two-way ANOVA with Bonferroni post-hoc tests for comparisons among multiple experimental groups. Data are expressed as mean ± S.E.M. P values $p<0.05$ were considered statistically significant.

**References**

1. Zhang X, Zhang R, Raab S, Zheng W, Wang J, Liu N, Zhu T, Xue L, Song Z, Mao J, Li K, Zhang H, Zhang Y, Han C, Ding Y, Wang H, Hou N, Liu Y, Shang S, Li C, Sebokova E, Cheng H, Huang PL. Rhesus macaques develop metabolic syndrome with reversible vascular dysfunction responsive to pioglitazone. *Circulation*. 2011;124:77–86.
2. Radu M, Lyle K, Hoeflich KP, Villamar-Cruz O, Koeppen H, Chernoff J. p21-Activated Kinase 2 Regulates Endothelial Development and Function through the Bmk1/Erk5 Pathway. *Mol Cell Biol*. 2015;35:3990–4005.
3. Agah R, Frenkel PA, French BA, Michael LH, Overbeek PA, Schneider MD. Gene recombination in postmitotic cells: Targeted expression of Cre recombinase provokes cardiac-restricted, site-specific rearrangement in adult ventricular muscle in vivo. *J Clin Invest*. 1997;100:169–179.
4. Werfel S, Jungmann A, Lehmann L, Ksienzyk J, Bekeredjian R, Kaya Z, Leuchs B, Nordheim A, Backs J, Engelhardt S, Katus HA, Müller OJ. Rapid and highly efficient inducible cardiac gene knockout in adult mice using AAV-mediated expression of Cre recombinase. *Cardiovasc Res*. 2014;104:15–23.
5. Liu W, Zi M, Jin J, Prehar S, Oceandy D, Kimura TE, Lei M, Neyses L, Weston AH, Cartwright EJ, Wang X. Cardiac-Specific Deletion of Mkk4 Reveals Its Role in Pathological Hypertrophic Remodeling but Not in Physiological Cardiac Growth. *Circ Res*. 2009;104:905–914.
6. Wiseman RL, Zhang Y, Lee KPK, Harding HP, Haynes CM, Price J, Ron D. Flavonol activation defines an unanticipated ligand binding site in the kinase-RNase domain of IRE1. *Molecular Cell*. 2010;38, 291–304.
7. Yamamoto K, Suzuki N, Wada T, Okada T, Yoshida H, Kaufman RJ, and Mori K. Human HRD1 Promoter Carries a Functional Unfolded Protein Response

Element to Which XBP1 but not ATF6 Directly Binds. *J Biochem*. 2008;144: 477–486.
8. Oda Y, Okada T, Yoshida H, Kaufman RJ, Nagata K, and Mori K. Derlin-2 and Derlin-3 are regulated by the mammalian unfolded protein response and are required for ER-associated degradation. *J Cell Biol*. 2006;172:383–393.
9. Wang H, Li Q, Shen Y, Sun A, Zhu X, Fang S, Shen Y. The ubiquitin ligase Hrd1 promotes degradation of the Z variant alpha 1-antitrypsin and increases its solubility. *Mol Cell Biochem*. 2011;346:137–145.
10. Streckfuss-Bömeke K, Wolf F, Azizian A, Stauske M, Tiburcy M, Wagner S, Hübscher D, Dressel R, Chen S, Jende J, Wulf G, Lorenz V, Schön MP, Maier LS, Zimmermann WH, Hasenfuss G, Guan K. Comparative study of human-induced pluripotent stem cells derived from bone marrow cells, hair keratinocytes, and skin fibroblasts. *Eur Heart J*. 2013;34:2618–2629.
11. Kelleher DJ, Kreibich G and Gilmore R. Oligosaccharyltransferase activity is associated with a protein complex composed of ribophorins I and II and a 48 kd protein. Cell. 1992;69:55-65.
12. Li C, Wong WH. Model-based analysis of oligonucleotide arrays: Expression index computation and outlier detection. *Proc Natl Acad Sci USA*. 2001;98:31–36.
13. Bolstad BM, Irizarry RA, Astrand M, Speed TP. A comparison of normalization methods for high density oligonucleotide aray data based on variance and bias. *Bioinformatics*. 2003;119:185–193.
14. Livak KJ, Schmittgen TD. Analysis of Relative Gene Expression Data Using Real-Time Quantitative PCR and the 2−ΔΔCT Method. *Methods*. 2001;25:402–408.

Supplemental Figure I

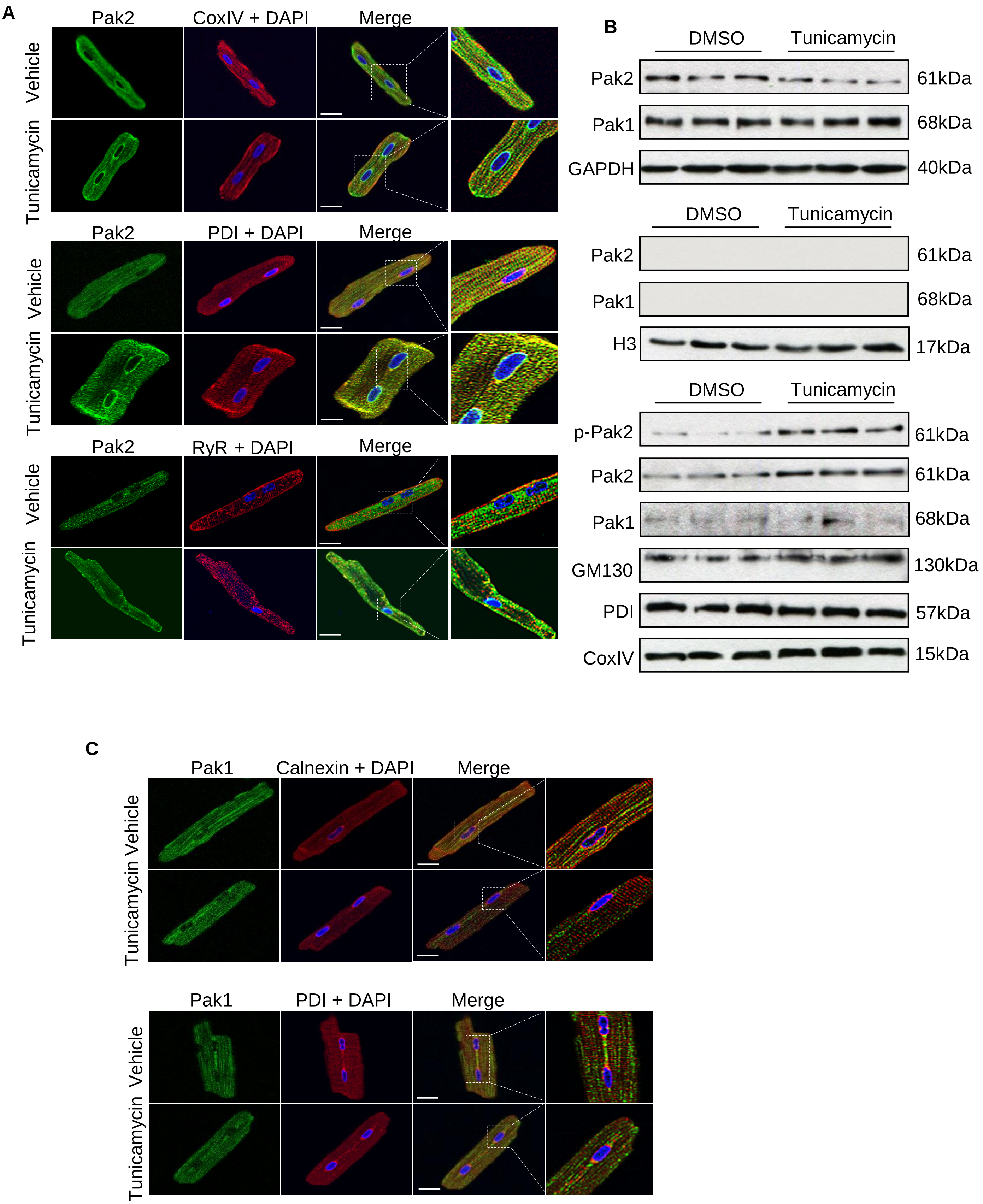

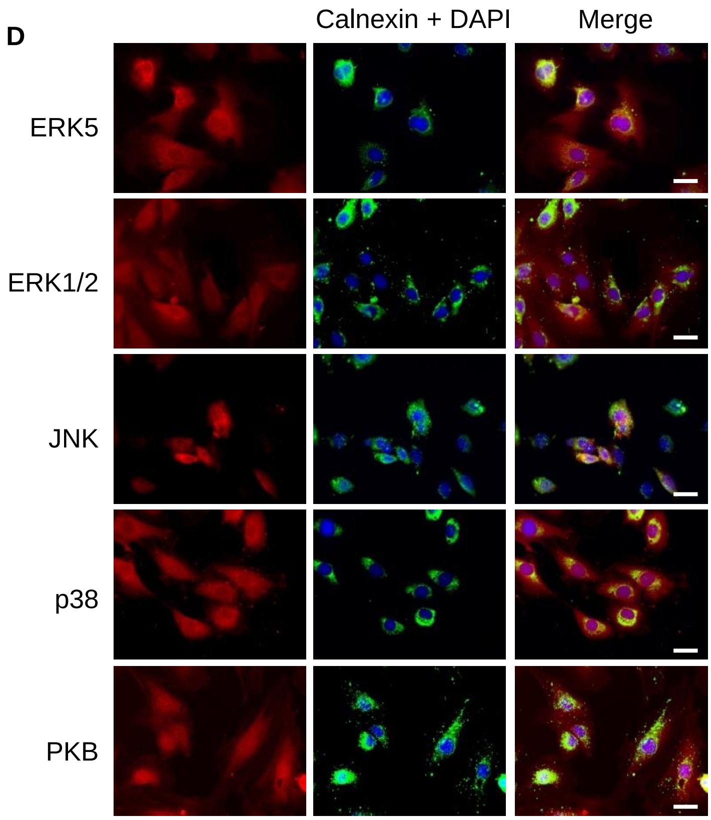


**Supplemental Figure I. Cellular localization of signaling molecules in cardiomyocytes.** (A) Immunofluorescent staining of ARCMs showed co-localization of Pak2 (green) and PDI (red, a soluble ER marker). However, no co-localization of Pak2 (green) and CoxIV (red, a mitochondria marker) or RyR (red, a SR marker) was observed in the absence and presence of Tunicamycin (5μg/ml, 2 hours). Scale bar: 20μm. (B) Immunoblotting analyses on fractional protein preparation demonstrated that Pak2 was increased in the organelle extracts of ARCMs (GM130 as a Golgi marker, PDI as an ER marker, and CoxIV as a mitochondria marker) by Tunicamycin treatment for 2 hours. Pak1 is dominantly expressed in the cytosol (GAPDH as a cytosol protein marker). No expression of Pak1 and Pak2 was detected in the nucleus (histone H3 as nuclear marker). (C) Immunofluorescent signal did not detect Pak1 present on ER in NRCMs (scale bar: 20μm). (D) Immunofluorescent staining of NRCMs showed a diffusing cytosolic expression pattern of ERK5, ERK1/2, JNK, p38, and PKB (red). Calnexin (green) indicated the ER membrane, and DAPI was for nuclei staining (scale bar: 20μm).

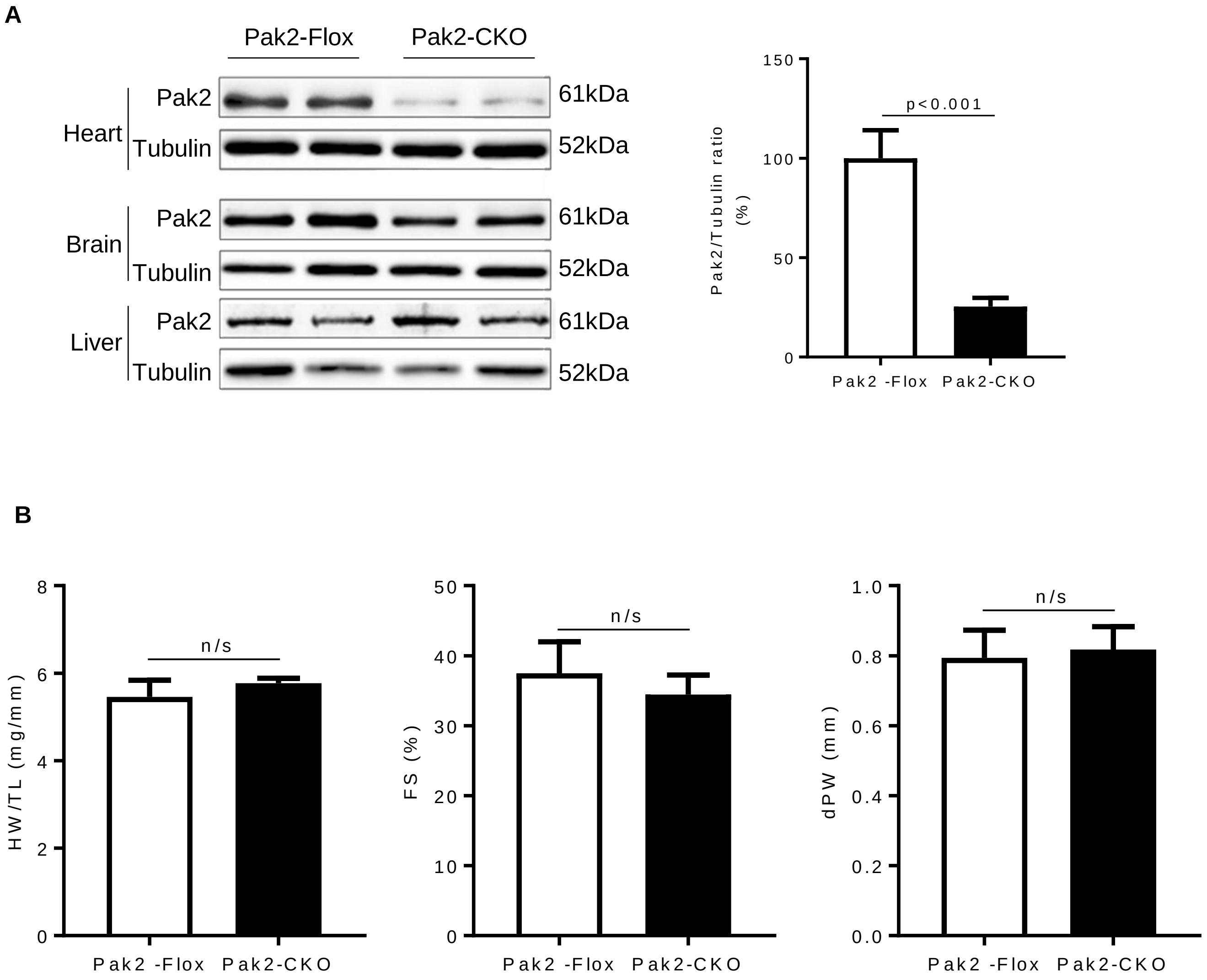


**Supplemental Figure II. Characterization of Pak2-CKO mice.** (A) Immunoblots showed approximately 75% decrease in Pak2 expression in ventricles of Pak2-CKO mice (quantitative data was shown in the right panel, n=6 mice per group). (B) Heart weight/tibial length ratio (HW/TL), fractional shortening (FS%) and end-diastolic left ventricular posterior wall thickness (dPW) were comparable between 8 weeks old Pak2-Flox and Pak2-CKO mice (n=7-8 mice per group). n/s not significant. Student's t-test were used for analyses. Data present as mean ± S.E.M.

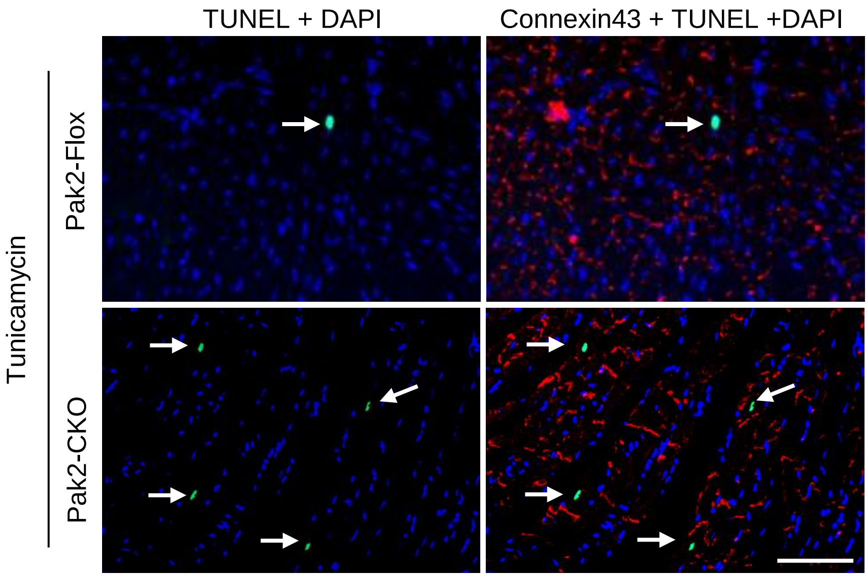


**Supplemental Figure III. Tunicamycin induces more apoptosis in Pak2-CKO hearts.** TUNEL assay by triple staining with DAPI (blue), anti-Connexin43 antibody (red) and TUNEL (green) determined more apoptosis in Pak2-CKO hearts with tunicamycin injection (scale bar: 20µm), arrows indicate TUNEL positive nuclei.

Supplemental Figure IV

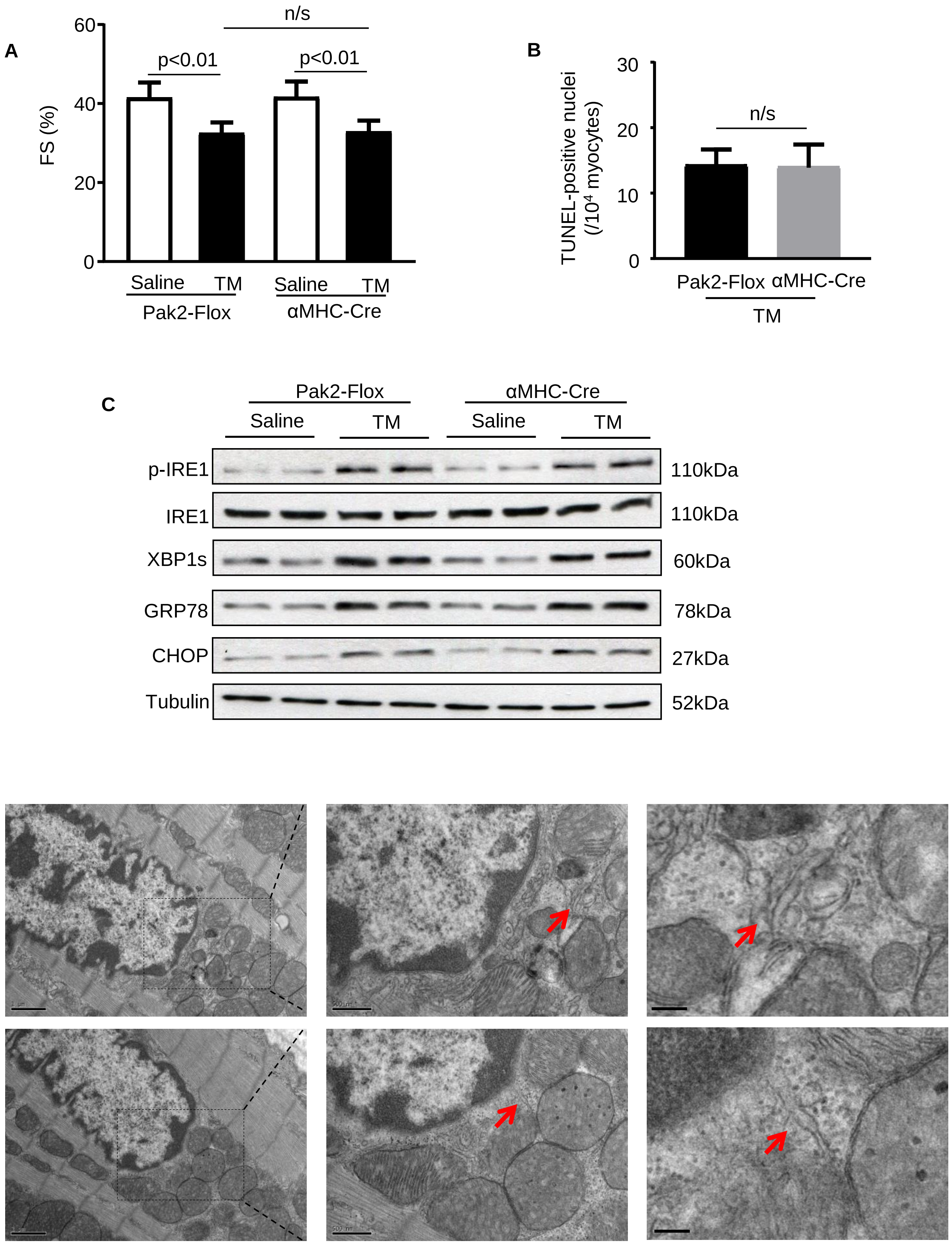

**Supplemental Figure IV. Comparison of Pak2-Flox and αMHC-Cre mice under tunicamycin treatment.** (A) Echocardiographic analyses showed a comparable reduction in fractional shortening (FS%) in Pak2-Flox and αMHC-Cre mice after 2 days of tunicamycin (TM, 2mg/kg) injection (n=5-6 mice per group). (B) TUNEL assay showed similar level of apoptosis in both mice (n=5-6 mice per group). (C) Immunoblots showed comparable IRE1 phosphorylation, and expression of XBP1s, GRP78 and CHOP in Pak2-Flox hearts compared to αMHC-Cre hearts with tunicamycin stress. Tubulin was the protein loading control. (D) Transmission electron microscopy detected the ultrastructure of ER in left ventricular papillary muscles. Middle images (scale bar: 500nm) are the higher magnifications of boxed areas in the left images (scale bar: 1μm). Right images highlighted the ER structure pointed by arrows in the middle images (scale bar: 100nm). Similar ER lumen was observed in Pak2-Flox and αMHC-Cre hearts treated with tunicamycin. The arrows indicate ER. n/s not significant. Student's t-test or two-way ANOVA with Bonferroni correction for post-hoc comparisons were used for analyses. Data present as mean ± S.E.M.

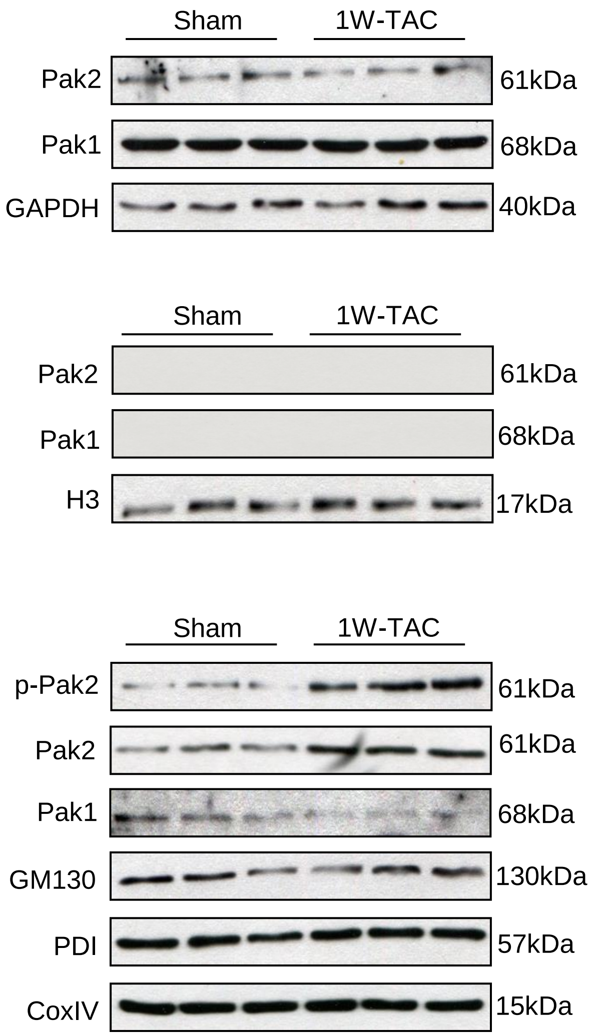


**Supplemental Figure V. Cellular localization of Pak2 in myocardium.** Immunoblotting analyses using fractional protein preparation demonstrated that Pak2 was increased in the organelle extracts (GM130 as a Golgi marker, PDI as an ER marker, CoxIV as a mitochondria marker) in Pak2-Flox hearts after 1 week TAC treatment. Pak1 is dominantly expressed in the cytosolic preparation (GAPDH as a cytosol protein marker). No expression of Pak1 and Pak2 was detected in the nuclear fraction (histone H3 as a nuclear marker).

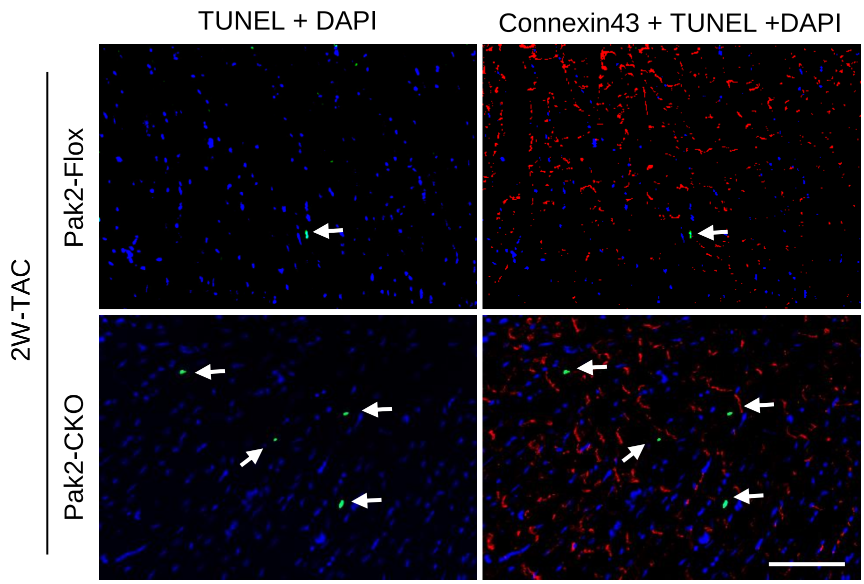


**Supplemental Figure VI. 2 weeks TAC induces more apoptosis in Pak2-CKO hearts.** TUNEL assay by triple staining with DAPI (blue), anti-Connexin43 antibody (red) and TUNEL (green) determined more apoptosis in Pak2-CKO hearts subject to 2weeks TAC (scale bar: 20µm), arrows indicate TUNEL positive nuclei.

# Supplemental Figure VII

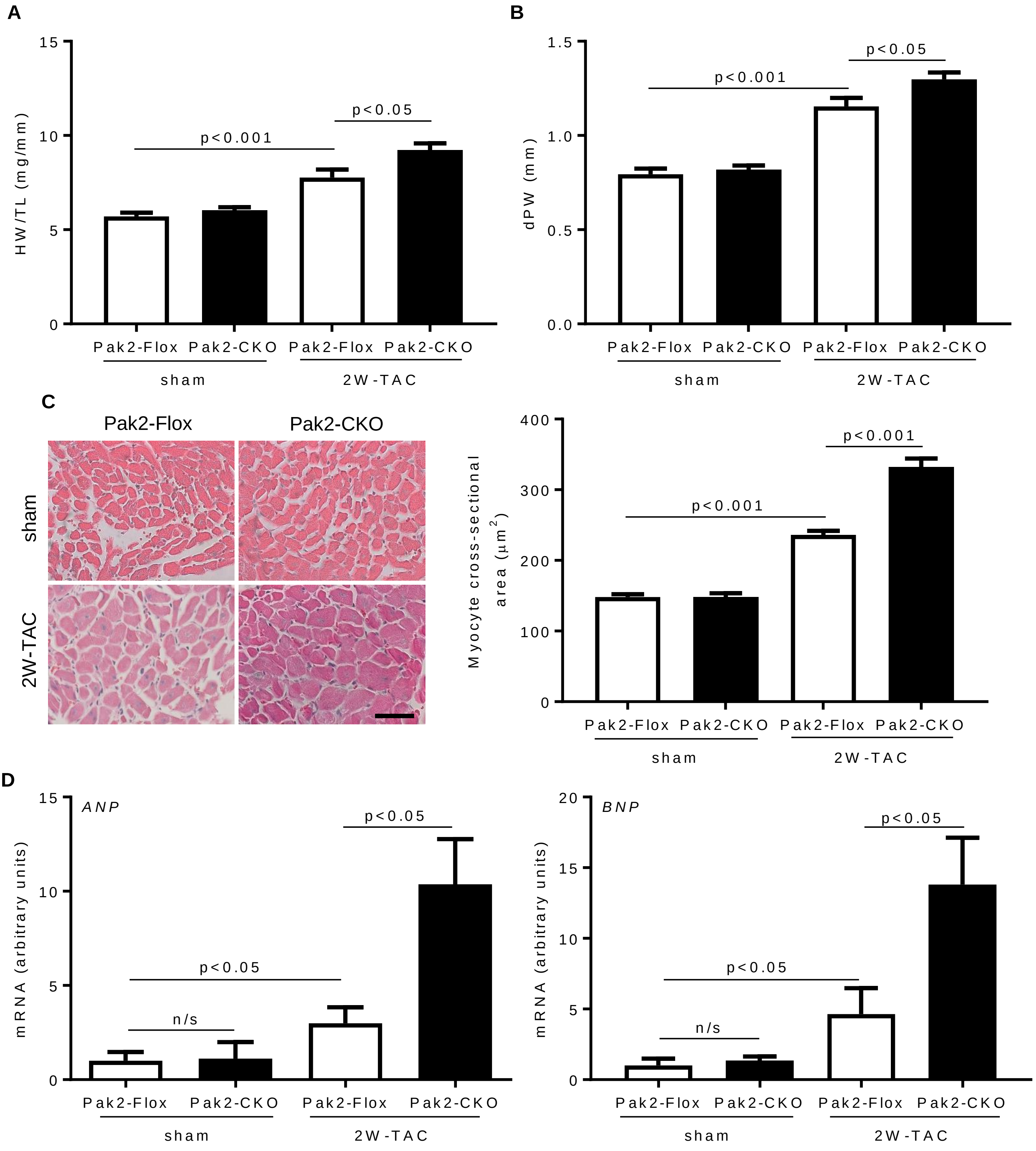

**E**

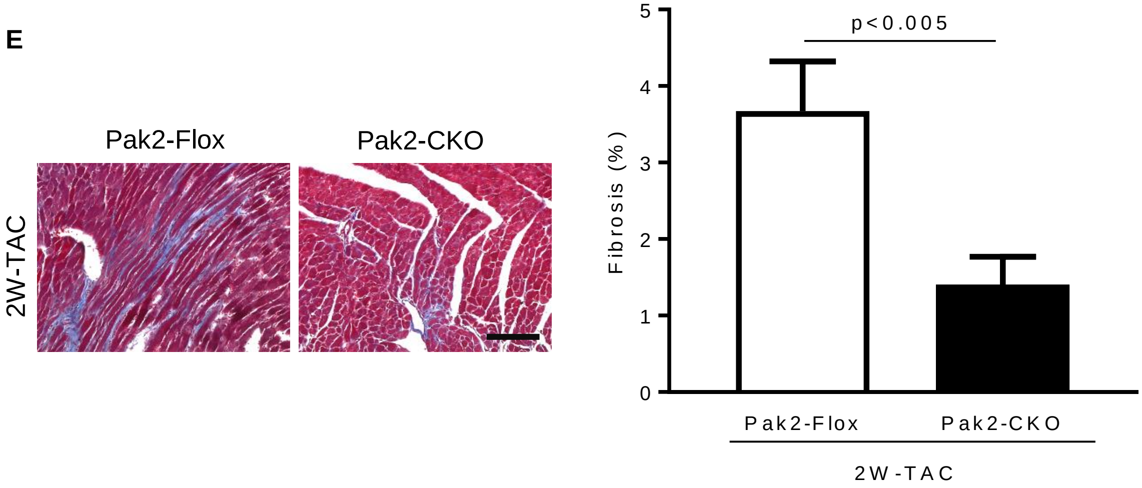


**Supplemental Figure VII. Pak2-CKO mice show augmented cardiac hypertrophy after 2 weeks TAC.** (A) heart weight/tibia length (HW/TL), (B) dPW determined by echocardiographic measurement and (C) Myocyte cross-sectional area (scale bar: 20µm) showed greater cardiac hypertrophy in Pak2-CKO compared to Pak2-Flox mice following 2 weeks TAC. (D) qPCR analyses of *ANP* and *BNP*. (E) Fibrosis was less in Pak2-CKO following 2 weeks TAC (scale bar: 20µm) (n=6-8 mice per group). Student's t-test or two-way ANOVA with Bonferroni correction for post-hoc comparisons were used for analyses. Data present as mean ± S.E.M.

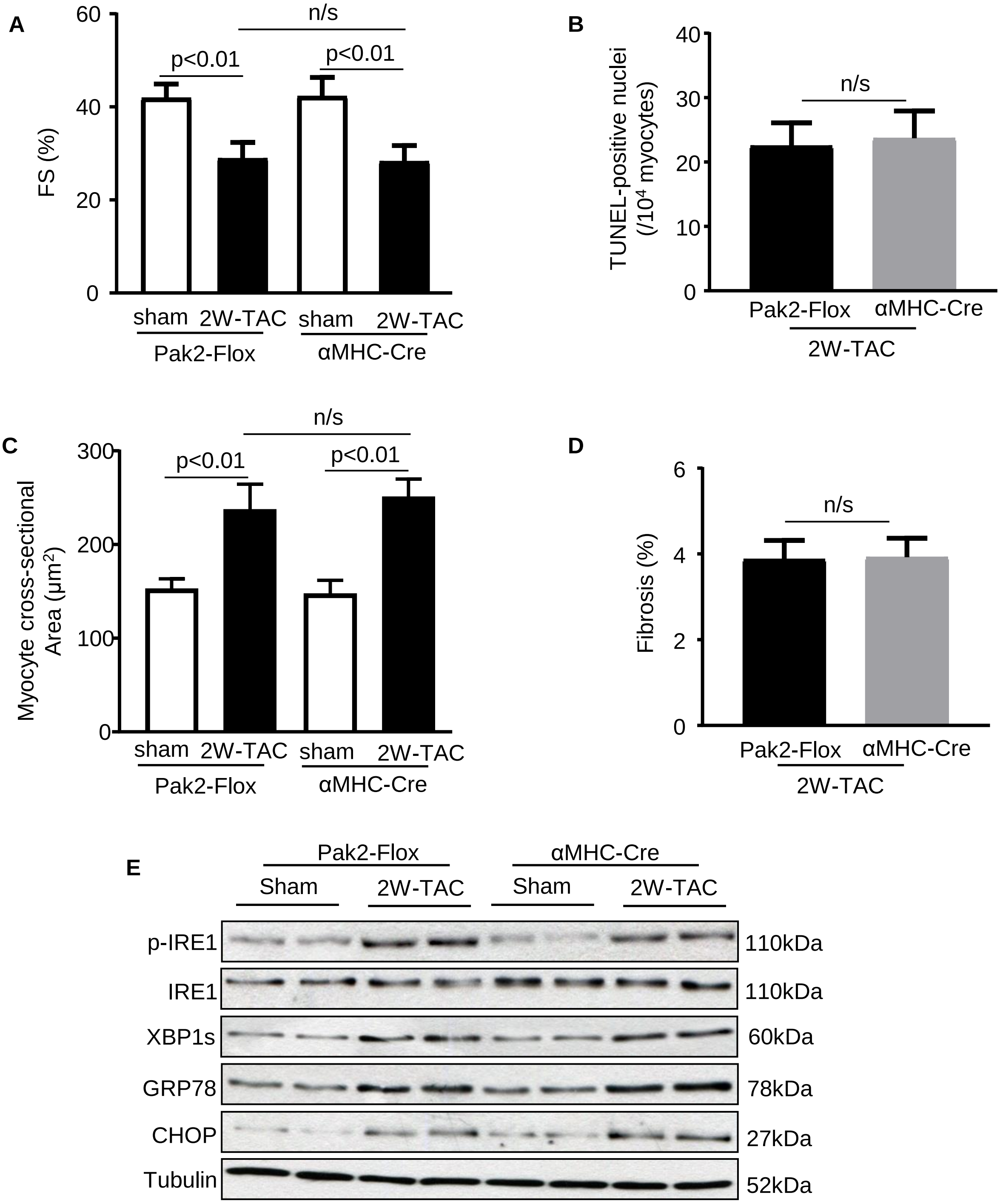


**Supplemental Figure VIII. Comparison of Pak2-Flox and αMHC-Cre mice under TAC stress.** (A) Echocardiographic analyses showed a comparable reduction in fractional shortening (FS%) in Pak2-Flox and αMHC-Cre mice after 2 weeks TAC (n=5 mice per group). Similar level of apoptosis (B), cross-sectional area (C), and fibrosis (D) were detected in both genotypes after 2 weeks TAC (n=5 mice per group). (E) Immunoblots showed comparable IRE1 phosphorylation, and expression of XBP1s, GRP78 and CHOP in Pak2-Flox hearts compared to αMHC-Cre hearts with tunicamycin stress. Tubulin was the protein loading control. n/s not significant. Student's t-test or two-way ANOVA with Bonferroni correction for post-hoc comparisons were used for analyses. Data present as mean ± S.E.M.

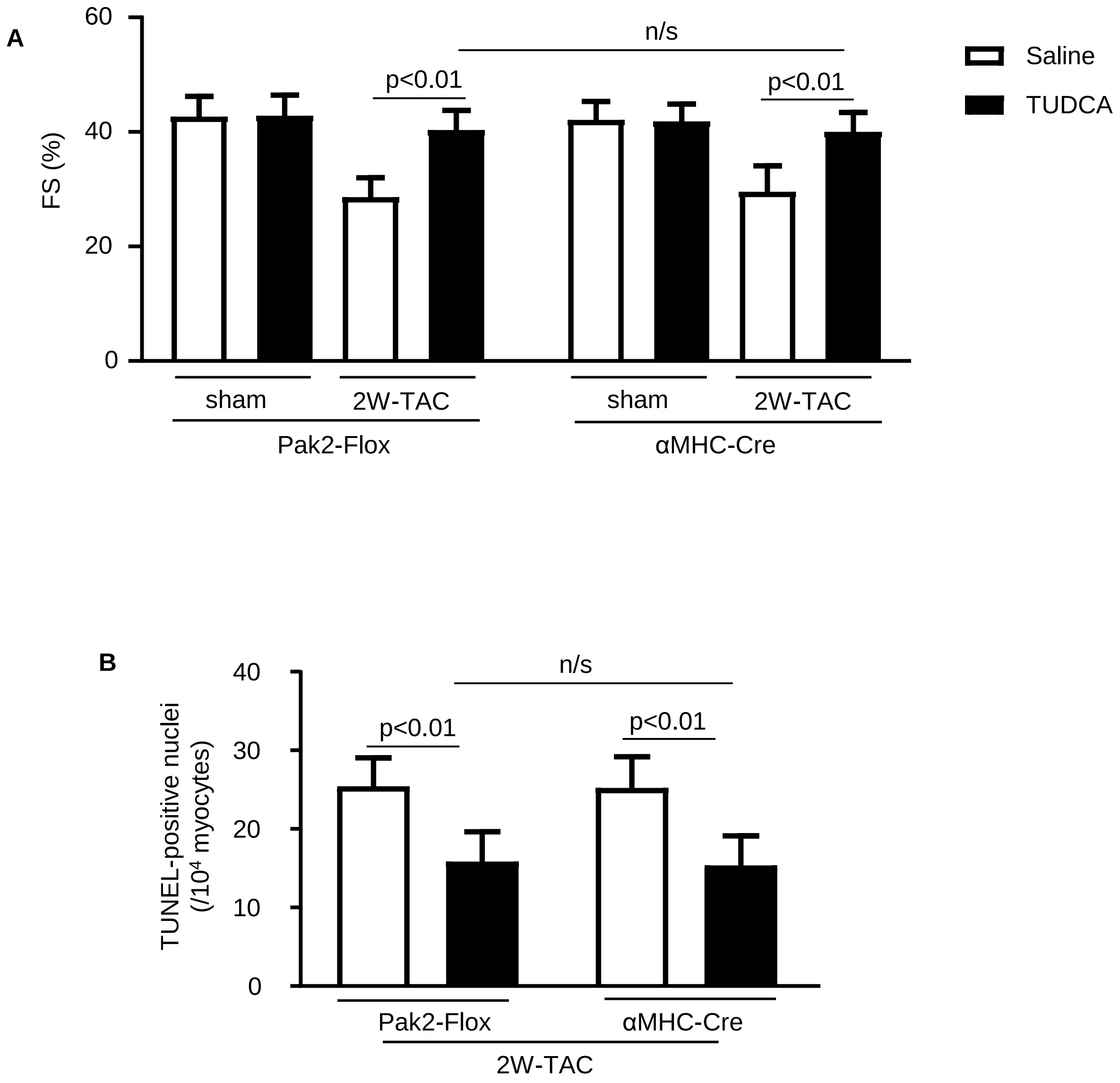
A
60
40
20
0
FS (%)
n/s
p<0.01
p<0.01
Saline
TUDCA
sham
2W-TAC
sham
2W-TAC
Pak2-Flox
αMHC-Cre
B
40
30
20
10
0
TUNEL-positive nuclei
(/10^4 myocytes)
n/s
p<0.01
p<0.01
Pak2-Flox
αMHC-Cre
2W-TAC

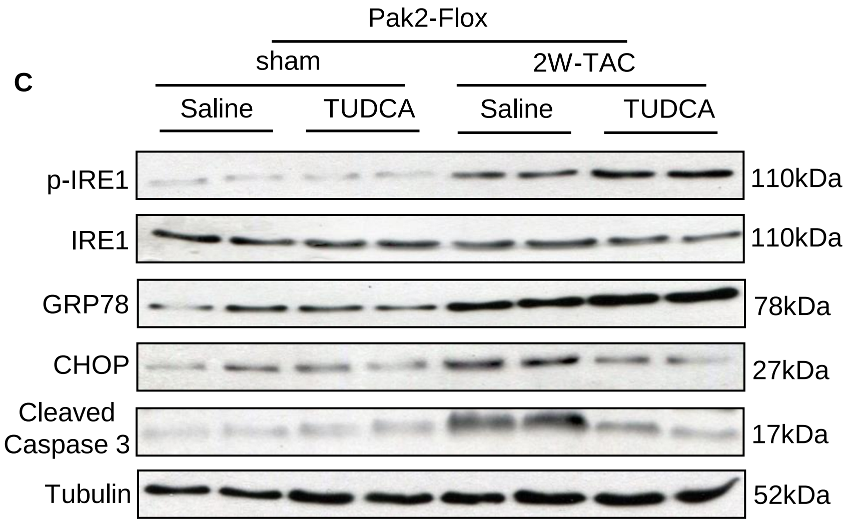


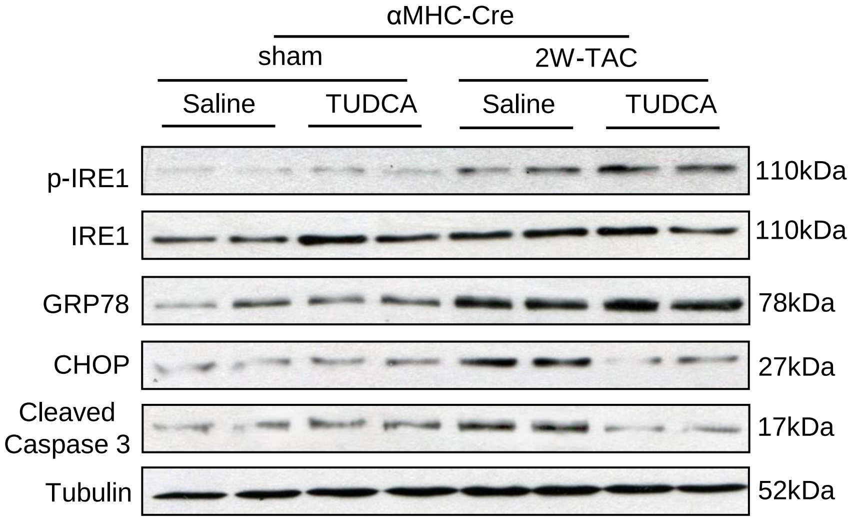


**Supplemental Figure IX. Comparison of Pak2-Flox and αMHC-Cre mice in response to TUDCA treatment.** (A) Comparable improvement in fractional shortening (FS%) and (B) similar apoptosis ratio in Pak2-Flox and αMHC-Cre mice with TUDCA treatment (300mg/kg/day) (n=5 mice per group). (C) Immunoblots showed similar changes of IRE1 phosphorylation, and expression of XBP1s, GRP78 and CHOP in Pak2-Flox hearts compared to αMHC-Cre hearts in response to TUDCA treatment. Tubulin was the protein loading control. n/s not significant. Two-way ANOVA with Bonferroni correction for post-hoc comparisons was used for analyses. Data present as mean ± S.E.M.

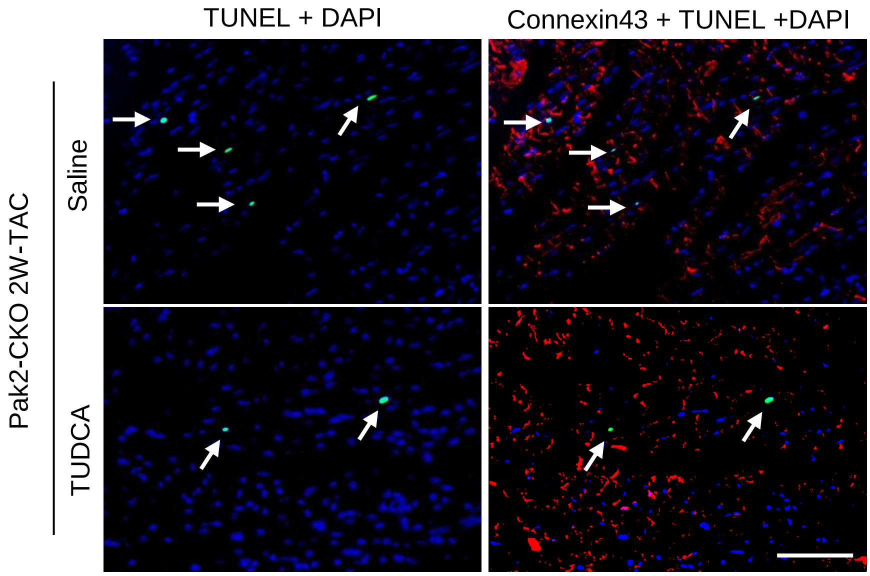


**Supplemental Figure X. TUDCA reduces TAC-induced apoptosis in Pak2-CKO heart.** TUNEL assay by triple staining with DAPI (blue), anti-Connexin43 antibody (red) and TUNEL (green) determined that TUDCA treatment reduced apoptosis in Pak2-CKO hearts stressed by 2 weeks TAC(scale bar: 20μm), arrows indicate TUNEL positive nuclei.

# Supplemental Figure XI

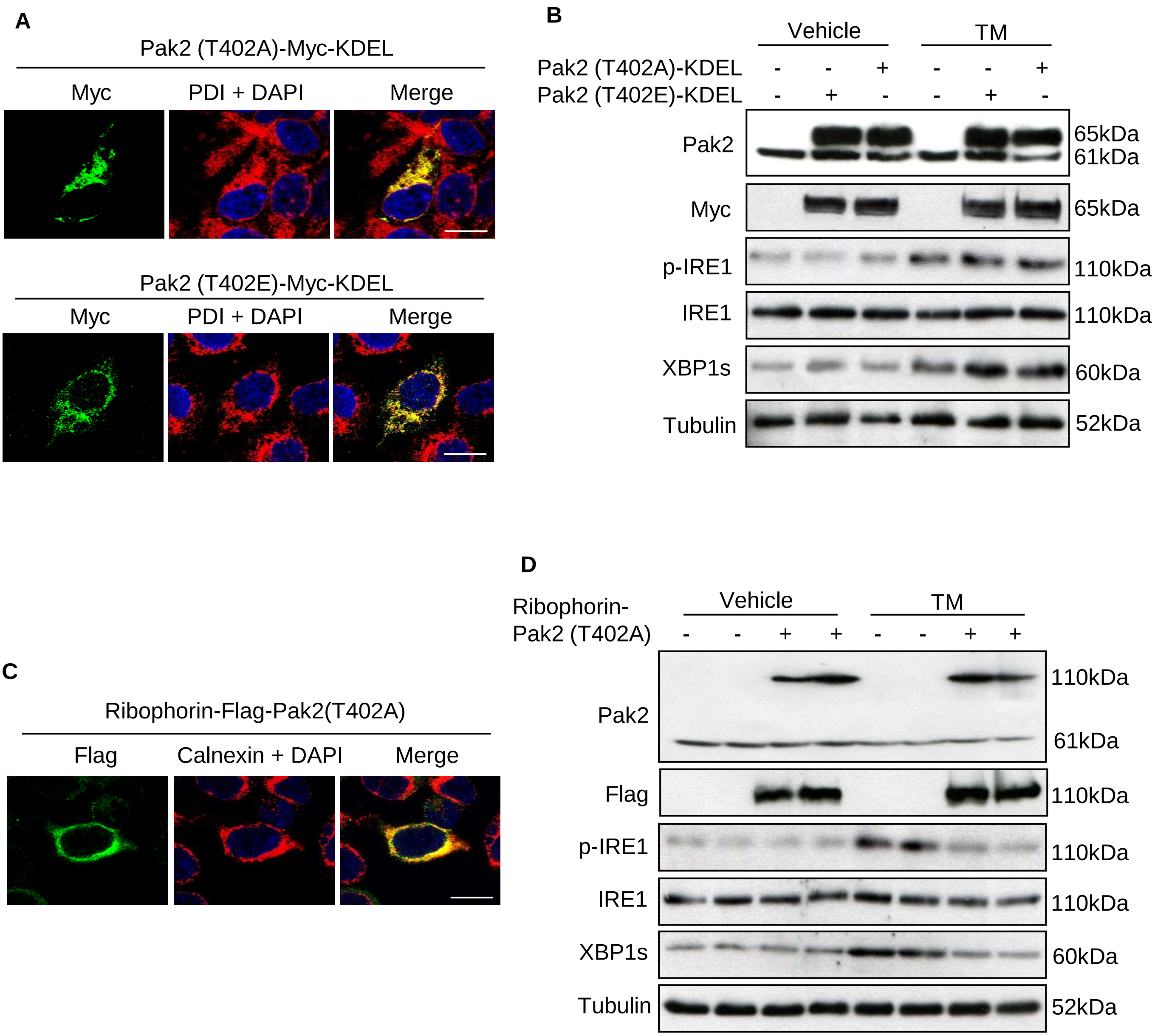


**Supplemental Figure XI. Pak2 action on IRE1-XBP1s is in close proximity to the ER membrane.** (A) pEF/Myc/ER-Pak2 (T402A)-KDEL and pEF/Myc/ER-Pak2 (T402E)-KDEL were transfected in H9C2 cardiomyocytes. Immunocytochemistry showed co-localization of Myc-tagged Pak2 (green) and PDI (red, a soluble ER marker). Scale bar: 20µm. (B) Immunoblots detected endogenous Pak2 (61kDa) and Myc-tagged ectopic Pak2 (65kDa). Tunicamycin (TM, increased the levels of IRE1 phosphorylation and XBP1s, which was not affected by ER lumen retention of Pak2-T402A and Pak2-T402E. (C) Ribophorin I (an ER transmembrane glycoproteins) N-terminal transmembrane domain fused with Flag-tagged Pak2-T402A was transfected in H9C2 cardiomyocytes. Immunocytochemistry showed co-localization of Flag-tagged kinase-dead Pak2 (green) and Calnexin (red, an ER membrane marker). Scale bar: 20µm. (D) Immunoblots detected endogenous Pak2 (61kDa) and Flag-tagged ectopic Pak2 (110kDa). ER membrane anchored Pak2-T402A abolished the levels of IRE1 phosphorylation and XBP1s induced by tunicamycin (TM, 5µg/ml, 2 hours). Tubulin was the proteinloading control.

Supplemental Figure XII

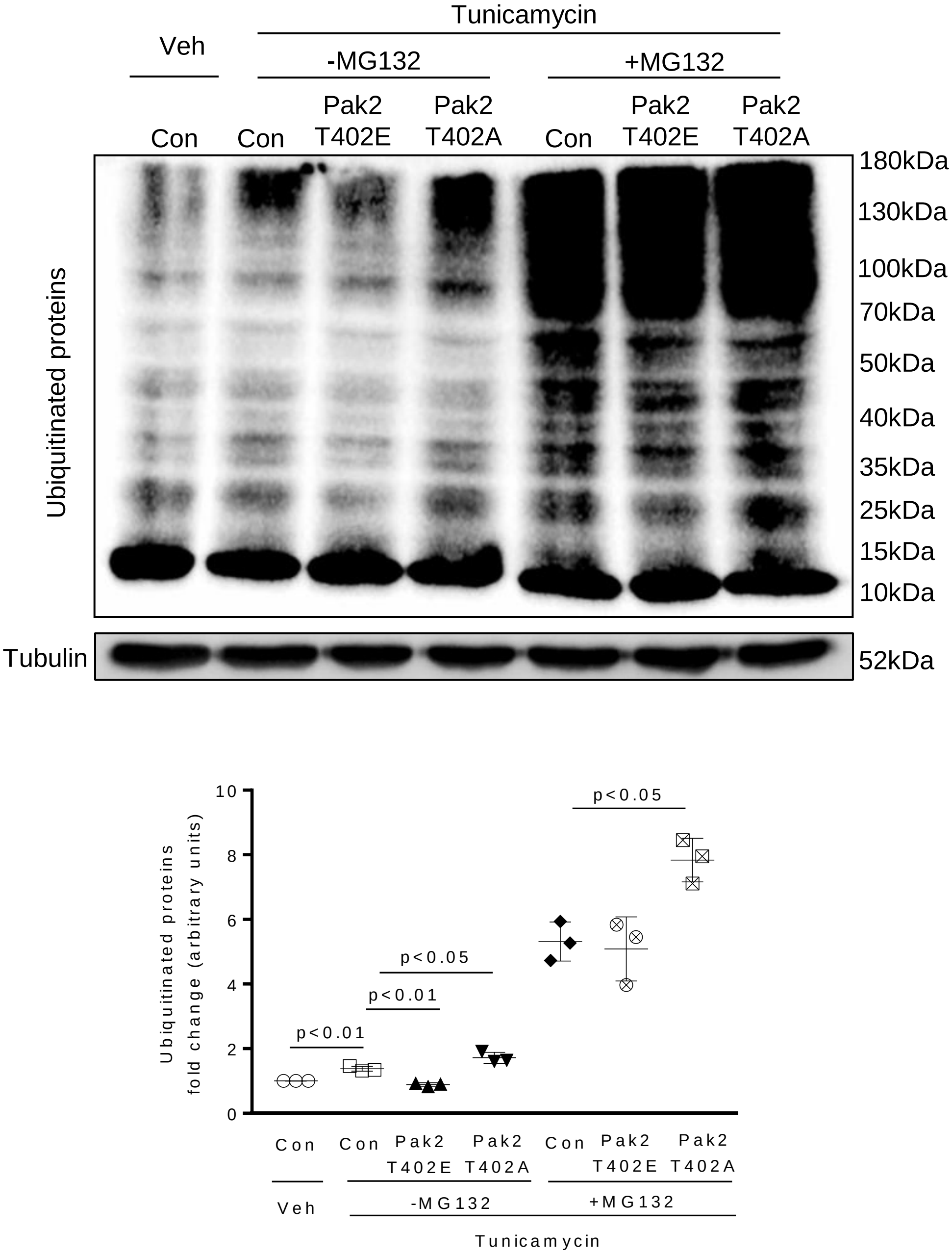


**Supplemental Figure XII. Pak2 inhibits proteins aggregation.** Immunoblots and quantification showed that tunicamycin (TM, 5μg/ml for 2 hours)-triggered aggregation of ubiquitin-conjugated proteins were abolished in Pak2-T402E overexpressed (constitutively active) H9C2 cardiomyocytes, whereas Pak2-T402A (kinase dead) overexpressed cardiomyocytes had increased ubiquitinated proteins under TM stress. In addition, TM-induced proteins aggregation was more enhanced in Pak2-T402A overexpressed cardiomyocytes in the presence of MG132 (2.5μM, a proteasome inhibitor). Tubulin was the protein loading control. Two-way ANOVA with Bonferroni correction for post-hoc comparisons was used for analyses. Data are presented as means ± SEM,

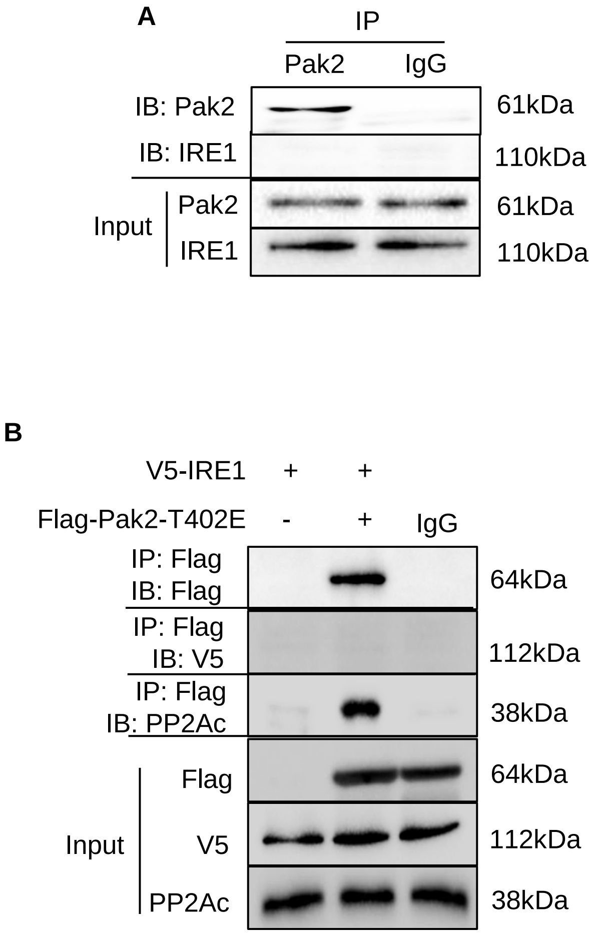


**Supplemental Figure XIII. Association of Pak2-PP2A and IRE1-PP2A.** (A) Immunoprecipitation failed to show endogenous association of Pak2 with IRE1 in H9C2 cells. (B) Immunoprecipitation in H9C2 cells with co-transfection of Flag-Pak2 and V5-IRE1 detected an association of Pak2 with endogenous PP2Ac; however, no interaction between Pak2 and IRE1 at basal condition was observed. IgG was control for nonspecific interaction.

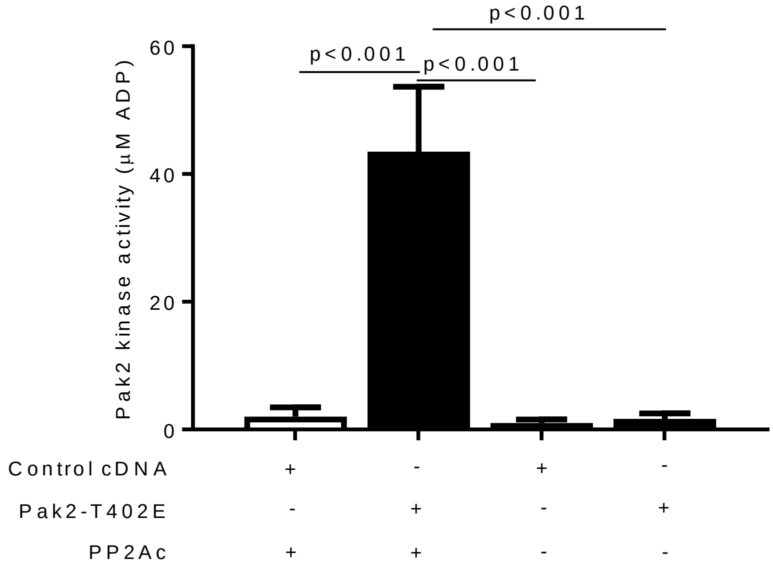


**Supplemental Figure XIV. Pak2 phosphorylates PP2Ac.** *In vitro* kinase assay detected that Pak2-T402E directly phosphorylated recombinant PP2Ac in H9C2 cells. ADP concentration was measured as readout of Pak2 kinase activity (n=6). One-way ANOVA with Bonferroni correction for post-hoc comparisons was used for analyses. Data present as mean ± S.E.M.

**A**

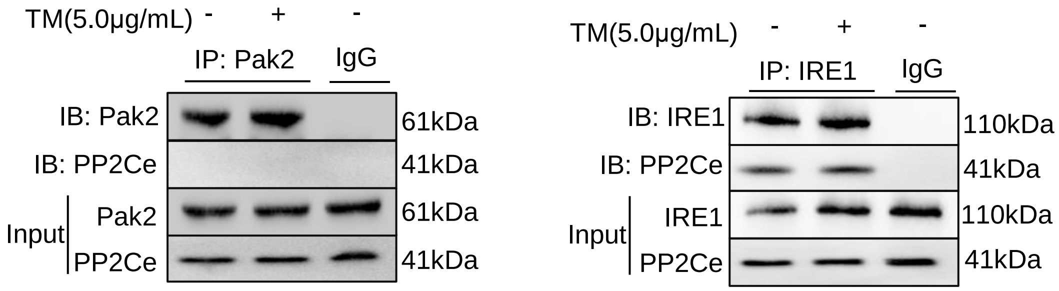


**B**

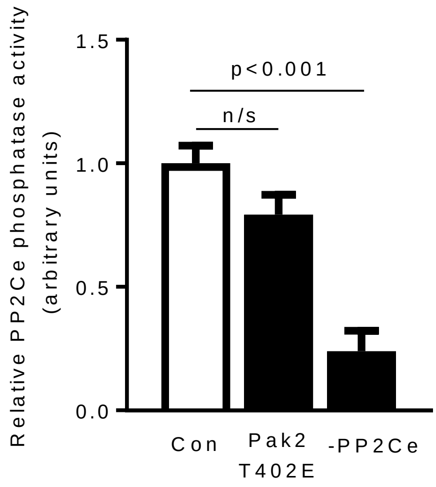


**Supplemental Figure XV. PP2Ce is not involved in Pak2 regulation of IRE1.** No-association of endogenous PP2Ce with Pak2 (left panel) or IRE1 (right panel) was observed by immunoprecipitation in NRCMs in the absence and presence of tunicamycin (TM) treatment. IgG was used as control for nonspecific interaction. (B) PP2Ce phosphatase activity assay in H9C2 cells detected that PP2Ce activity was not decreased by Pak2-T402E (n=6). No existence of PP2Ce enzyme is the negative control. n/s not significant. One-way ANOVA with Bonferroni correction for post-hoc comparisons was used for analyses. Data present as mean ± S.E.M.

A

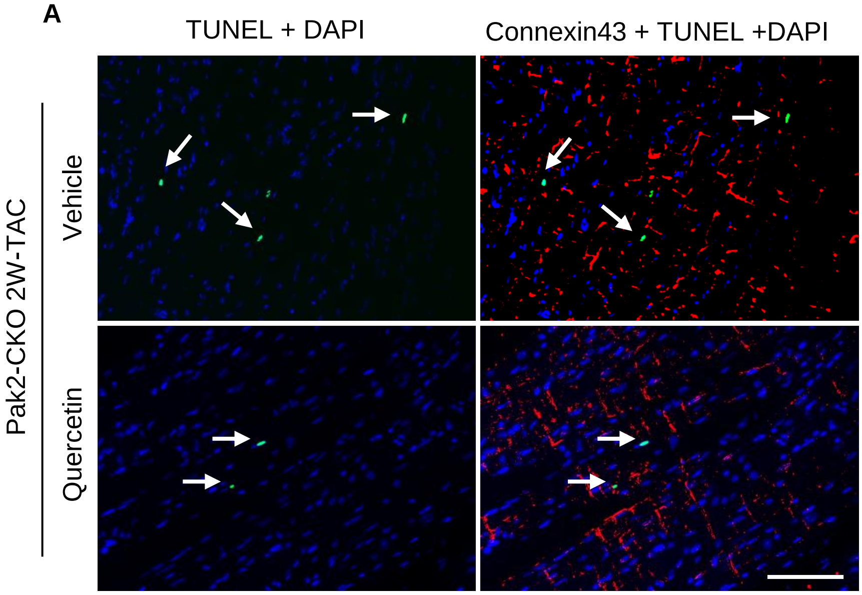
TUNEL + DAPI
Connexin43 + TUNEL +DAPI
Pak2-CKO 2W-TAC
Vehicle
Quercetin

B

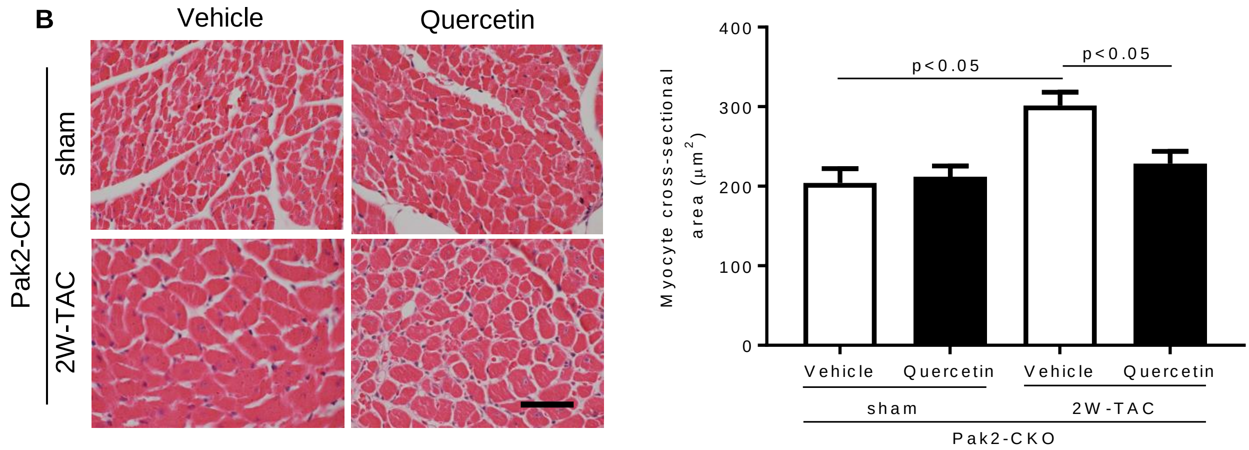
Vehicle
Quercetin
Pak2-CKO
sham
2W-TAC
Myocyte cross-sectional area ($\mu m^2$)
400
300
200
100
0
p<0.05
p<0.05
Vehicle
Quercetin
Vehicle
Quercetin
sham
2W-TAC
Pak2-CKO

C

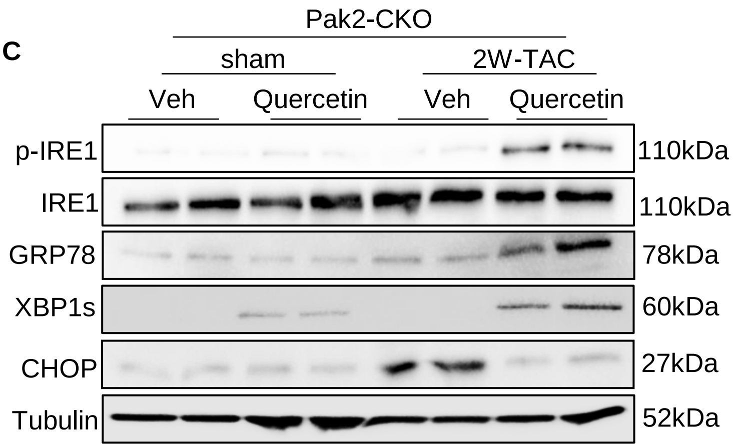
Pak2-CKO
sham
2W-TAC
Veh
Quercetin
Veh
Quercetin
p-IRE1
IRE1
GRP78
XBP1s
CHOP
Tubulin
110kDa
110kDa
78kDa
60kDa
27kDa
52kDa

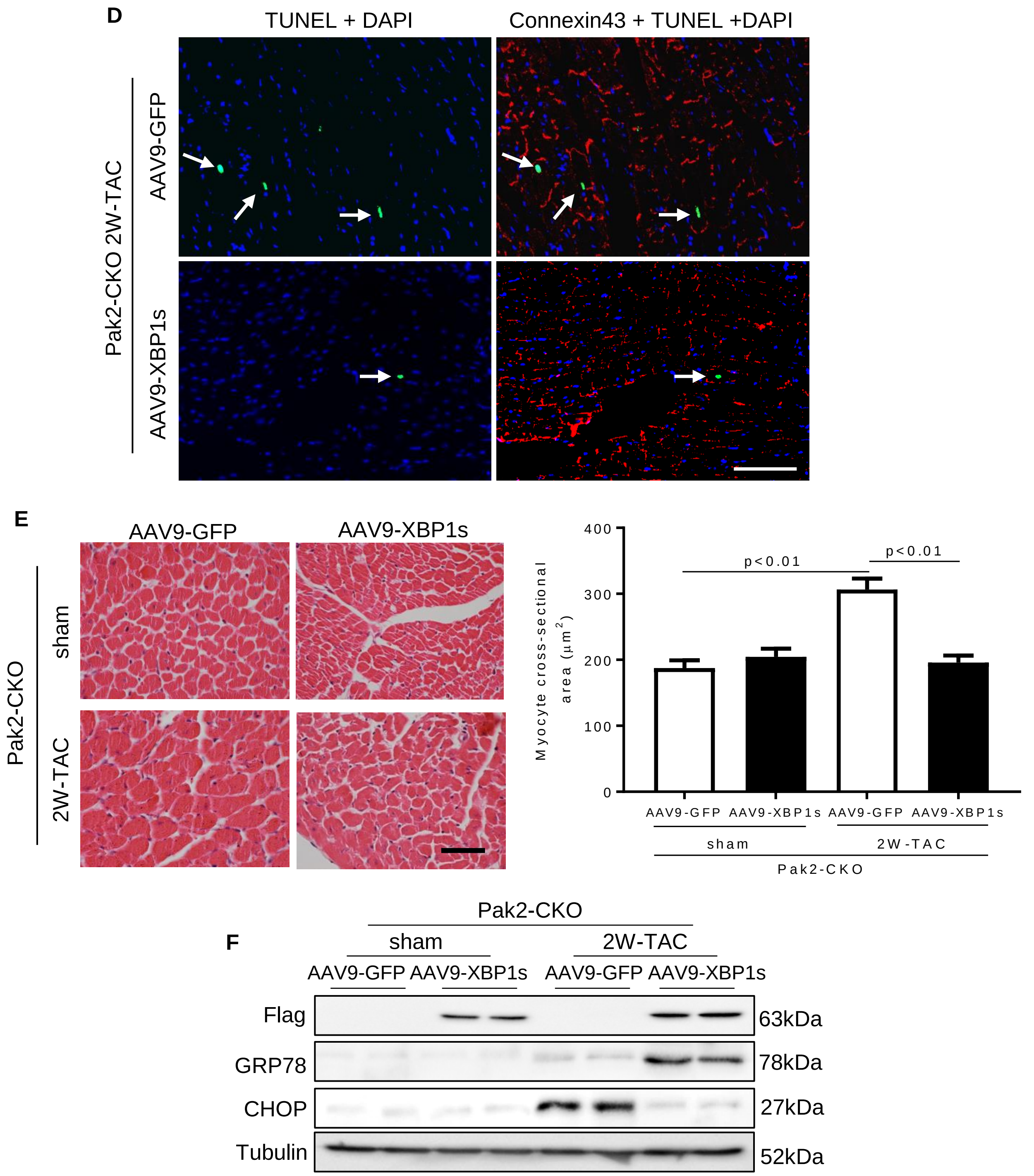


**Supplemental Figure XVI. Activation of IRE1-XBP1s signalling rescued ER stress response in Pak2-CKO hearts.** (A) TUNEL assay by triple staining with DAPI (blue), anti-Connexin43 antibody (red) and TUNEL (green) determined less apoptosis in Pak2-CKO hearts with Queretin treatment (10mg/kg) (scale bar: 20µm), arrows indicate TUNEL positive nuclei. (B) Queretin decreased cross-sectional area in Pak2-CKO hearts after 2 weeks TAC (scale bar: 20µm). (C) Queretin increased the levels of IRE1 phosphorylation, XBP1s and GRP78, but decreased CHOP expression. (D) TUNEL assay determined less apoptosis in Pak2-CKO hearts with AAV9-XBP1s injection (scale bar: 20µm. (E) AAV9-XBP1s decreased cross-sectional area in Pak2-CKO hearts after 2 weeks TAC (scale bar: 20µm). (F) AAV9-XBP1s attenuated CHOP expression. Tubulin was the protein loading control. Two-way ANOVA with Bonferroni correction for post-hoc comparisons was used for analyses. Data present as mean ± S.E.M.

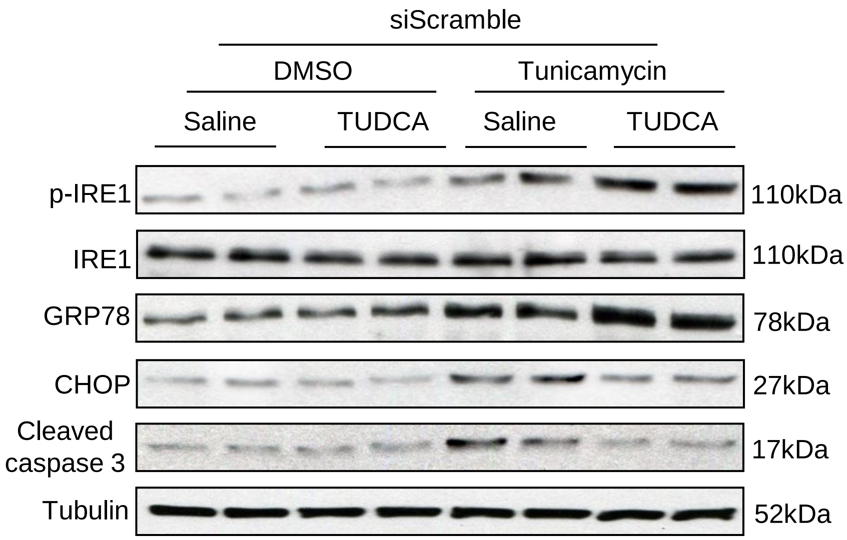


**Supplemental Figure XVII: TUDCA enhances ER stress response in human iPSC-derived cardiomyocytes (iPSC-CMs).** Immunoblots exhibited that IRE1 phosphorylation and GRP78 expression induced by tunicamycin (2µg/mL) in iPSC-CMs was enhanced by TUDCA treatment (200 µg/mL). However, CHOP and cleaved caspase 3 was attenuated by TUDCA treatment. Tubulin was the protein loading control.

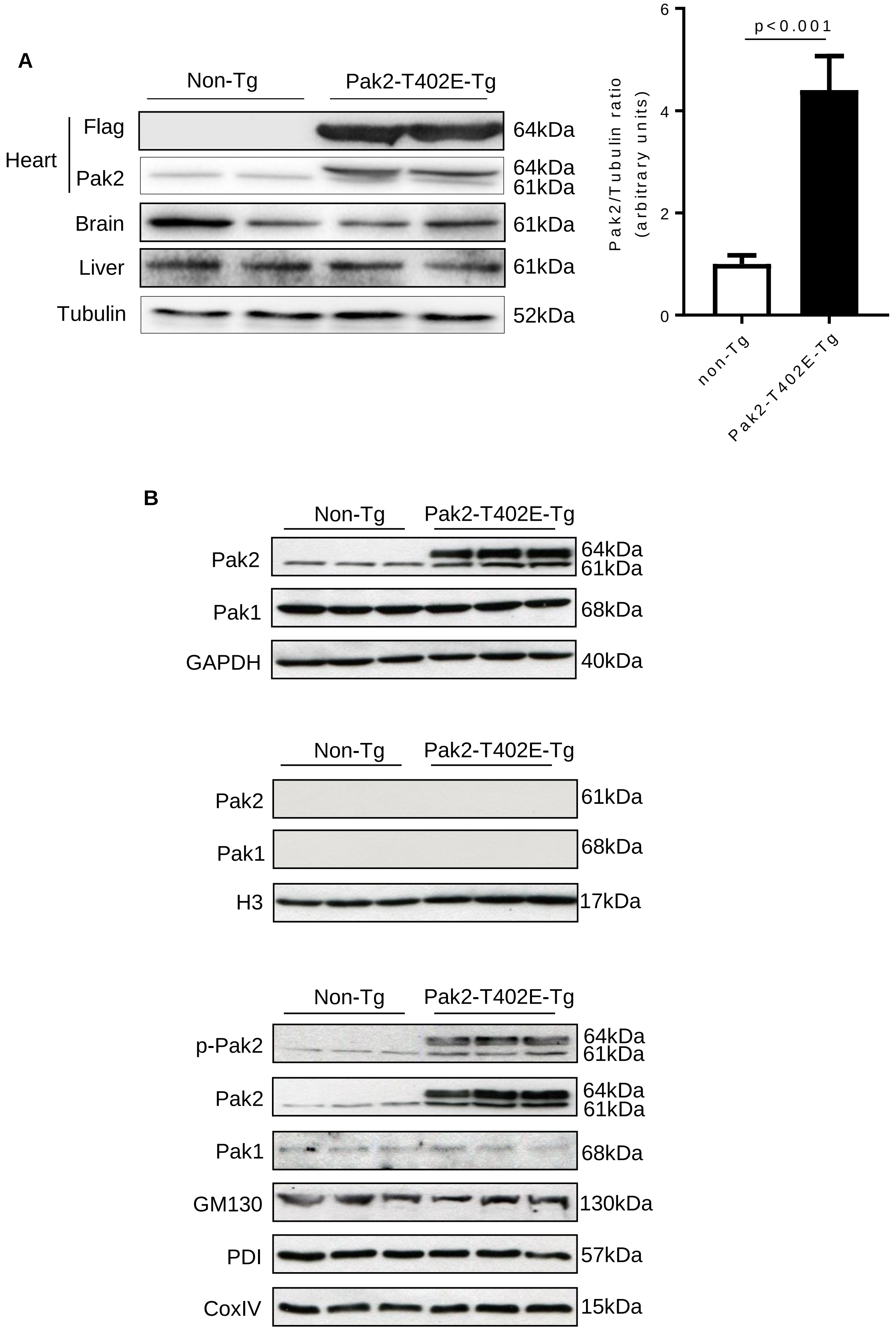
A
Non-Tg
Pak2-T402E-Tg
Heart
Flag
Pak2
Brain
Liver
Tubulin
64kDa
64kDa
61kDa
61kDa
61kDa
52kDa
Pak2/Tubulin ratio (arbitrary units)
p<0.001
0
2
4
6
non-Tg
Pak2-T402E-Tg
B
Non-Tg
Pak2-T402E-Tg
Pak2
Pak1
GAPDH
64kDa
61kDa
68kDa
40kDa
Non-Tg
Pak2-T402E-Tg
Pak2
Pak1
H3
61kDa
68kDa
17kDa
Non-Tg
Pak2-T402E-Tg
p-Pak2
Pak2
Pak1
GM130
PDI
CoxIV
64kDa
61kDa
64kDa
61kDa
68kDa
130kDa
57kDa
15kDa

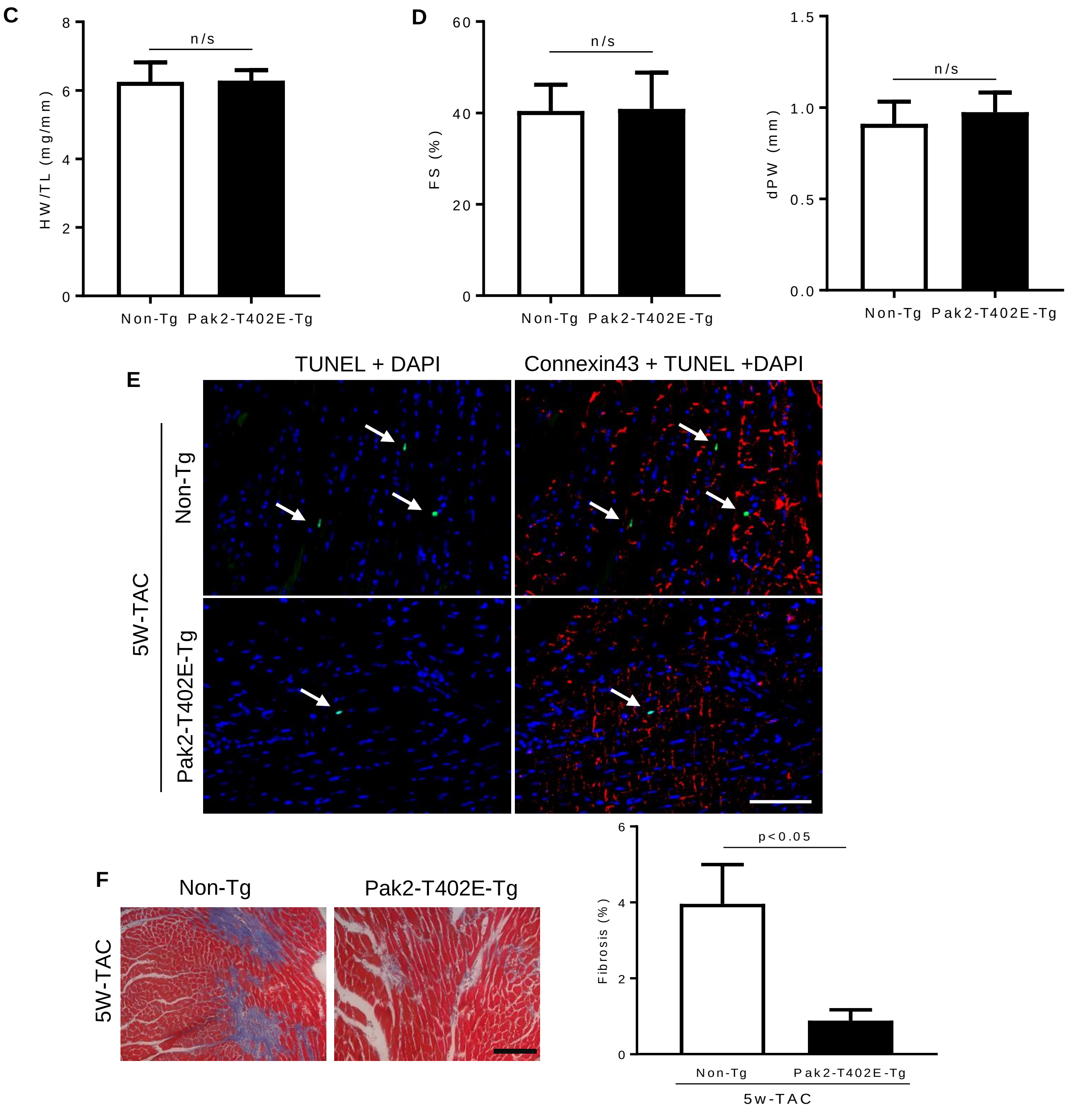


**Supplemental Figure XVIII. Characterization of Pak2-T402E-Tg mice.** (A) Immunoblots and quantification showed that the ectopic Pak2 level in Pak2-T402E-Tg was approximately 4.2 times of that in non-Tg mice. (B) Immunoblots on fractional protein preparation demonstrated that Pak2 was increased in the cytosolic and organelle extracts (GAPDH as a cytosol protein marker, GM130 as a Golgi marker, PDI as an ER marker, a CoxIV as mitochondria marker, histone H3 as nuclear marker) in Pak2-T402E-Tg hearts. Immunoblots detected endogenous Pak2 (61kDa) and Flag-tagged ectopic Pak2 (64kDa). (C) HW/TL, (D) FS% and dPW were comparable between 8 weeks old Non-Tg and Pak2-T402E-Tg mice (n=6-7 mice per group). (E) TUNEL assay by triple staining with DAPI (blue), anti-Connexin43 antibody (red) and TUNEL (green) determined less apoptosis in Pak2-T402E-Tg hearts after 5W-TAC (scale bar: 20μm), arrows indicate TUNEL positive nuclei. (F) Fibrosis content was less in Pak2-T402E-Tg hearts than in Non-Tg hearts after 5 weeks TAC (scale bar: 20μm). n/s not significant. Student’s t-test was used for analyses. Data present as mean ± S.E.M.

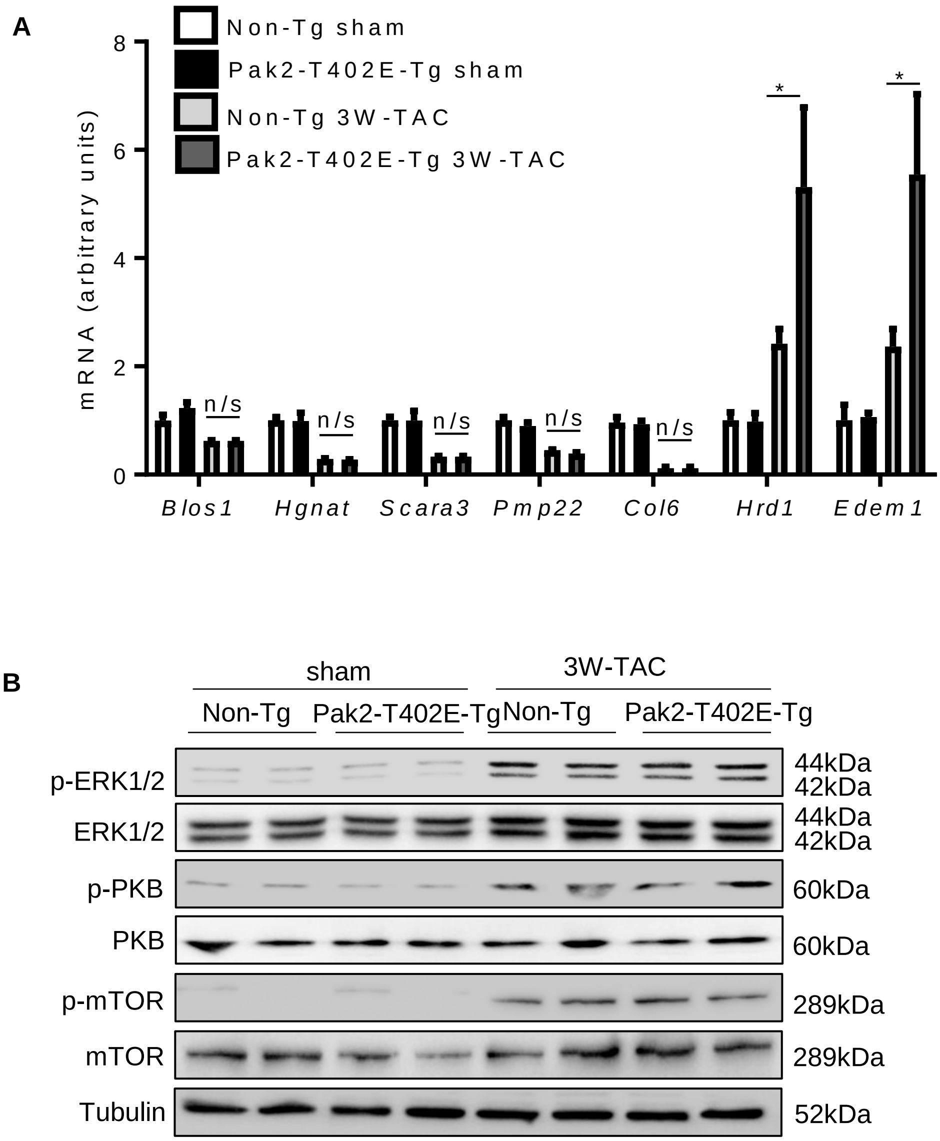


**Supplemental Figure XIX. Screening stress molecules in Pak2-T402E Tg mice.** (A) Transcript levels of RIDD-specific substrates. *Blos1*, *Hgnat*, *Scara3*, *Pmp22*, *Col6, Hrd1* and *Edem1* were measured by qPCR from (n=6 mice per group). * P<0.05 Pak2-T402E-Tg vs. Non-Tg after TAC. n/s not significant. Two-way ANOVA with Bonferroni correction for post-hoc comparisons was used for analyses. Data present as mean ± S.E.M. (B) Immunoblotting analyses showed comparable changes in phosphorylation levels of ERK1/2, PKB and mTOR in Non-Tg and Pak2-T402E-Tg mice after 3 weeks TAC. Tubulin was the protein loading control.

Supplemental Figure XX

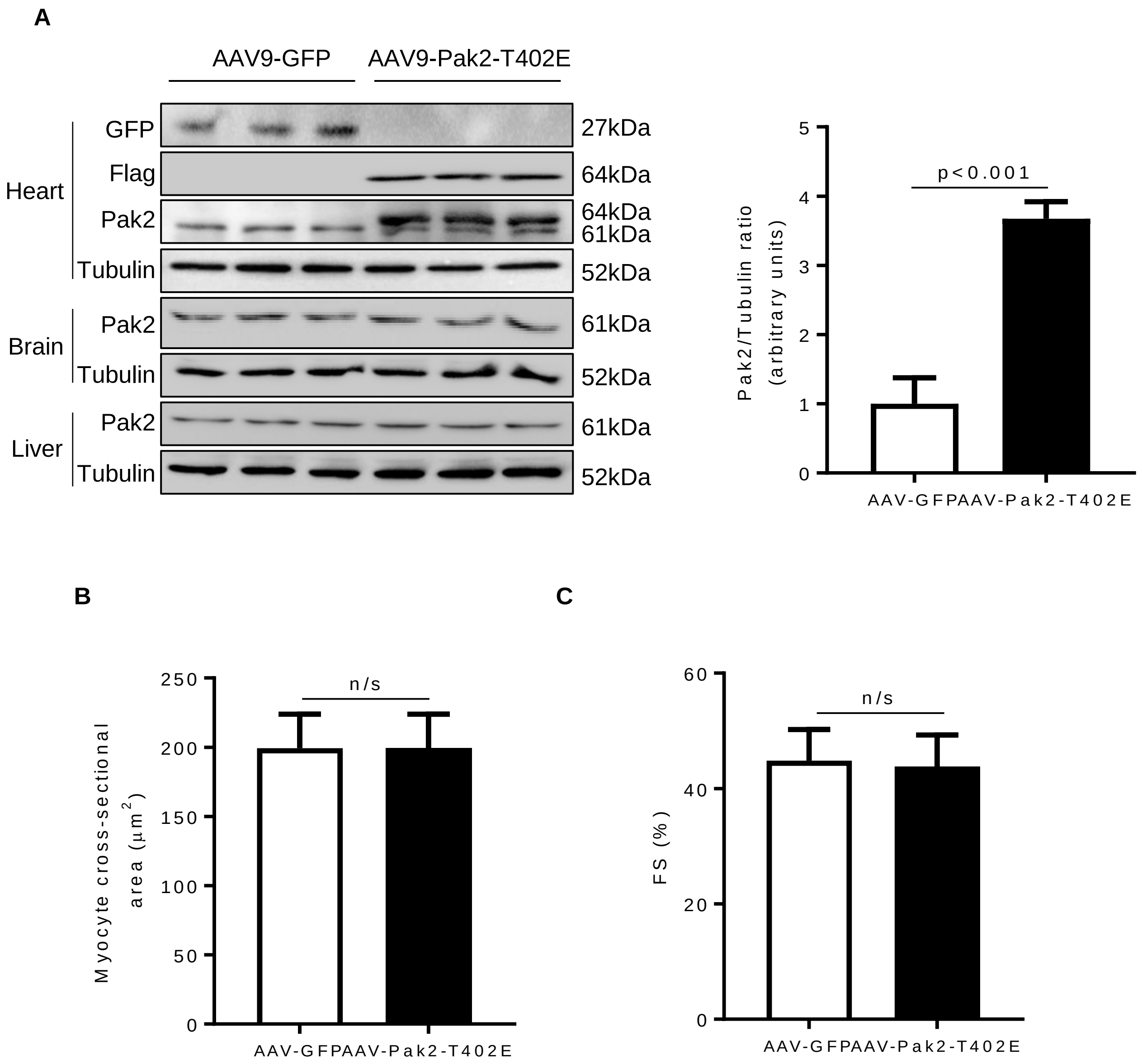


**Supplemental Figure XX. Characterization of AAV9-Pak2-T402E injected mice.** (A) Immunoblots showed that the ectopic Pak2 expression level in AAV9-Pak2-T402E injected C57BL/6N mice was 3.5 times of that in mice injected with AAV9-GFP (control) (quantitative data was shown). (B) Myocyte cross-sectional area and (C) FS% were comparable between AAV9-GFP and AAV9-Pak2-T402E injected C57BL/6N mice 7 days after injection (n=5 mice per group). n/s not significant. Student's t-test was used for analyses. Data present as mean ± S.E.M.

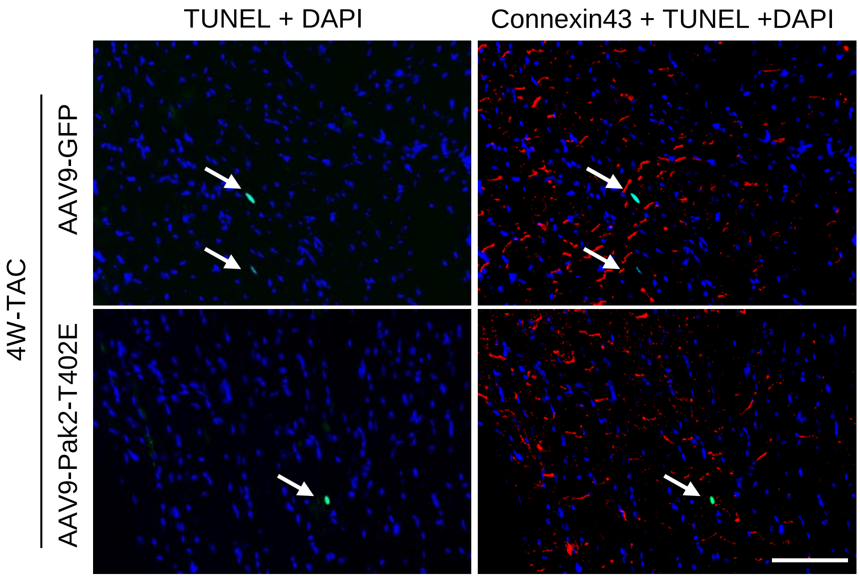


**Supplemental Figure XXI. AAV9-Pak2-T402E reduces apoptosis induced by TAC.** TUNEL assay by triple staining with DAPI (blue), anti-Connexin43 antibody (red) and TUNEL (green) determined less apoptosis in AAV9-Pak2-T402E injected hearts subject to 4 weeks TAC (scale bar: 20µm), arrows indicate TUNEL positive nuclei.

**Echocardiographic parameters of Pak2-Flox and Pak2-CKO mice following TAC**

| | sham | | 1W-TAC | |
|---|---|---|---|---|
| | Pak2-Flox | Pak2-CKO | Pak2-Flox | Pak2-CKO |
| FS (%) | 34.59±2.88 | 37.38±2.71 | 27.65±1.45 | 15.42±1.21$^{\#}$ |
| EF (%) | 65.35 ±2.97 | 60.14±1.39 | 57.39±1.86 | 38.11±2.65$^{\#}$ |
| dPW (mm) | 0.62±0.04 | 0.67±0.03 | 0.89±0.03 | 1.03±0.05* |
| LVEDD (mm) | 3.73±0.05 | 3.64+0.11 | 3.85±0.04 | 4.09±0.08 |
| LVESD (mm) | 2.44±0.12 | 2.30±0.16 | 2.78+0.05 | 3.46+0.11* |
| HR (bpm) | 431.33±6.81 | 432±11.64 | 446.83±11.68 | 418.33±14.66 |

| | sham | | 2W-TAC | |
|---|---|---|---|---|
| | Pak2-Flox | Pak2-CKO | Pak2-Flox | Pak2-CKO |
| FS (%) | 36.99±2.32 | 36.22±2.33 | 22.69±1.63 | 12.75±1.43$^{\#}$ |
| EF (%) | 58.14±4.69 | 62.07±3.55 | 50.41±1.82 | 36.80±2.63* |
| dPW (mm) | 0.79±0.03 | 0.82±0.02 | 1.15±0.04 | 1.30±0.04* |
| LVEDD (mm) | 3.62±0.09 | 3.69±0.12 | 3.94±0.09 | 4.23±0.10 |
| LVESD (mm) | 2.28±0. 12 | 2.37+0.14 | 3.05+0.12 | 3.70+0.15* |
| HR (bpm) | 434.16±13.38 | 442.33+14.15 | 432.60±5.77 | 421.85±10.59 |

FS%, fraction shortening; EF ejection fraction: dPW, end-diastolic left ventricular posterior wall thickness; LVEDD, diastolic left ventricular internal dimension; LVESD, systolic left ventricular internal dimension; HR, heart rate. n=5-9 per group. Two-way ANOVA with Bonferroni correction for post-hoc comparisons was used for analyses. Data are presented as means ± SEM, *P≤0.05, $^{\#}$P≤0.01 versus Pak2-Flox-TAC mice.

**Echocardiographic parameters of non-Tg and Pak2-T402E-Tg mice following TAC**

| | sham | | 3W-TAC | |
|---|---|---|---|---|
| | Non-Tg | Pak2-T402E-Tg | Non-Tg | Pak2-T402E-Tg |
| FS (%) | 40.42±2.58 | 40.87±3.25 | 24.17±1.65 | 36.25±3.43$^{\#}$ |
| EF (%) | 60.64+3.855 | 60.31+5.97 | 42.87+4.10 | 56.85+2.90* |
| dPW (mm) | 0.85±0.04 | 0.91±0.05 | 1.22±0.07 | 1.03±0.04* |
| LVEDD (mm) | 3.57±0.15 | 3.55±0.15 | 3.87±0.05 | 3.59±0.16 |
| LVESD (mm) | 2.14±0.18 | 2.12±0.19 | 2.93±0.08 | 2.31±0.20* |
| HR (bpm) | 422.00±10.94 | 432.83±8.92 | 445.83±7.66 | 427.28±12.50 |

| | sham | | 5W-TAC | |
|---|---|---|---|---|
| | Non-Tg | Pak2-T402E-Tg | Non-Tg | Pak2-T402E-Tg |
| FS (%) | 40.21±3.00 | 40.76±5.02 | 25.34±2.77 | 37.50±2.91* |
| EF (%) | 65.28±4.19 | 63.918±6.98 | 48.28±4.72 | 64.29±2.96* |
| dPW (mm) | 0.89±0.05 | 0.95±0.04 | 1.33±0.04 | 1.08±0.04$^{\#}$ |
| LVEDD (mm) | 3.51±0.14 | 3.64±0.17 | 3.65±0.17 | 3.66+0.10 |
| LVESD (mm) | 2.10±0.13 | 2.18±0.25 | 2.74±0.21 | 2.30+0.16 |
| HR (bpm) | 428.60±5.51 | 429.20±6.23 | 436.50±6.84 | 426.66±9.53 |

FS%, fraction shortening; EF ejection fraction: dPW, end-diastolic left ventricular posterior wall thickness; LVEDD, diastolic left ventricular internal dimension; LVESD, systolic left ventricular internal dimension; HR, heart rate. n=5-7 per group. Two-way ANOVA with Bonferroni correction for post-hoc comparisons was used for analyses. Data are presented as means ± SEM, *P≤0.05, $^{\#}$P≤0.01 versus Non-Tg-TAC mice.

Supplemental Table III

**Echocardiographic parameters of mice injected with AAV9-GFP or AAV9-Pak2-T402E following TAC**

| | sham | | 4W-TAC | |
|---|---|---|---|---|
| | AAV9-GFP | AAV9-Pak2-T402E | AAV9-GFP | AAV9-Pak2-T402E |
| FS (%) | 40.42±2.58 | 41.55±3.89 | 19.79±2.64 | 34.27±4.24* |
| EF (%) | 68.26±3.91 | 73.39±5.81 | 40.00±4.67 | 59.68±5.65* |
| dPW (mm) | 0.68±0.03 | 0.63±0.02 | 0.97±0.06 | 0.69±0.03[#] |
| LVEDD (mm) | 3.56±0.11 | 3.38+0.14 | 3.84±0.09 | 3.67±0.12 |
| LVESD (mm) | 2.13±0.14 | 2.00±0.21 | 3.09±0.15 | 2.44±0.21* |
| HR (bpm) | 440.60±4.19 | 421.80±10.63 | 432.17±4.31 | 426.14±5.67 |

FS%, fraction shortening; EF ejection fraction: dPW, end-diastolic left ventricular posterior wall thickness; LVEDD, diastolic left ventricular internal dimension; LVESD, systolic left ventricular internal dimension; HR, heart rate. n=5-7 per group. Two-way ANOVA with Bonferroni correction for post-hoc comparisons was used for analyses. Data are presented as means ± SEM, *P≤0.05, [#]P≤0.01 versus AAV9-GFP-TAC mice.
